# The infinitesimal model with dominance

**DOI:** 10.1093/genetics/iyad133

**Published:** 2023-07-14

**Authors:** Nicholas H Barton, Alison M Etheridge, Amandine Véber

**Affiliations:** Institute of Science and Technology, Am Campus I, A-3400 Klosterneuberg, Austria; Department of Statistics, University of Oxford, 24–29 St Giles, OX1 3LB Oxford, UK; MAP5, Université Paris Cité, CNRS, 45 rue des Saints-Pères, 75006 Paris, France

**Keywords:** infinitesimal model, dominance

## Abstract

The classical infinitesimal model is a simple and robust model for the inheritance of quantitative traits. In this model, a quantitative trait is expressed as the sum of a genetic and an environmental component, and the genetic component of offspring traits within a family follows a normal distribution around the average of the parents’ trait values, and has a variance that is independent of the parental traits. In previous work, we showed that when trait values are determined by the sum of a large number of additive Mendelian factors, each of small effect, one can justify the infinitesimal model as a limit of Mendelian inheritance. In this paper, we show that this result extends to include dominance. We define the model in terms of classical quantities of quantitative genetics, before justifying it as a limit of Mendelian inheritance as the number, *M*, of underlying loci tends to infinity. As in the additive case, the multivariate normal distribution of trait values across the pedigree can be expressed in terms of variance components in an ancestral population and probabilities of identity by descent determined by the pedigree. Now, with just first-order dominance effects, we require two-, three-, and four-way identities. We also show that, even if we condition on parental trait values, the “shared” and “residual” components of trait values within each family will be asymptotically normally distributed as the number of loci tends to infinity, with an error of order $1/\sqrt{M}$. We illustrate our results with some numerical examples.

## Teaser

The classical infinitesimal model is a simple and robust model for the inheritance of quantitative traits. In previous work, Barton, Etheridge, and Véber showed that—when trait values are determined by the sum of a large number of Mendelian factors, each of small effect—one can justify the infinitesimal model as limit of Mendelian inheritance. Here, the authors extend these results to include a nonlinear dominance effect.

## Introduction

In the classical infinitesimal model, a quantitative trait is expressed as the sum of a genetic and a nongenetic (environmental) component, and the genetic component of offspring traits within a family follows a normal distribution around the average of the parents’ trait values, and has a variance that is independent of the trait values of the parents. With inbreeding, the variance decreases in proportion to relatedness. When trait values are determined by the sum of a large number of Mendelian factors, each of small effect, as we show in Barton *et al.* (2017), one can justify the infinitesimal model as a limit of Mendelian inheritance. Crucially, the results of Barton *et al.* (2017) show that the evolutionary forces such as random drift and population structure are captured by the pedigree; conditioning on that pedigree, and trait values in the population in all generations before the present, the within-family distributions in the present generation will be given by a multivariate normal, with variance determined by that in the ancestral population and probabilities of identity by descent that can be deduced from the pedigree. If some traits in the pedigree are unknown, then averaging with respect to the ancestral distribution, the multivariate normality is preserved. It was also shown that under some forms of epistasis, trait values within a family are still normally distributed, although the mean will no longer be a simple function of the traits in the parents (as there are epistatic components which cannot be observed directly).

We emphasize that as a result of selection, population structure, and so on, the trait distribution *across* the population can be far from normal; the infinitesimal model as we define it only asserts that the *within-family* distributions of the genetic component of the trait are Gaussian, with a variance–covariance matrix that is determined entirely by that in an ancestral population and the probabilities of identity determined by the pedigree. Moreover, as a result of the multivariate normality, conditioning on some of the trait values within that pedigree has predictable effects on the mean and variance within and between families. In other words, knowing the trait values for some individuals in the population does not distort the multivariate normality of the distribution of the unobserved traits, and the mean and covariances of these traits may be derived explicitly (albeit after rather tedious calculations).

In this paper, we show that this extraordinary robustness of the infinitesimal model extends to include dominance. The distribution of the genetic part of the trait will once again be a multivariate normal distribution whose mean and variance is expressed in terms of the variance components in an ancestral population and probabilities of identity by descent determined by the pedigree, but now, with just first-order dominance effects, the identities required will involve up to four genes. As with the case of epistasis, the mean is not a simple function of the trait values in the parents, and there is nontrivial covariance between families. One can think of the genetic component of the trait values within a family as consisting of two parts. Both are normally distributed. In the additive case, the first reduces to the mean of the trait values of the parents; with dominance it will be random (even if we condition on knowing the parental traits), but the same for all individuals in the family. What is at first sight surprising is that even if we condition on knowing the trait values of the parents, this shared quantity is normally distributed. Assuming there is no mutation to ease the presentation [the effect of mutation was studied in Barton *et al.* (2017)], our first contribution is to show how to calculate its mean and variance from knowledge of variance components in the ancestral population and the pedigree, both with and without knowledge of the trait values of the parents. Knowing the trait values of the parents shifts the mean in a predictable way, the variance is independent of the parental trait values. The second part of the trait value, which is independent for each offspring in the family, is independent of the first; it encodes the randomness of Mendelian inheritance. It is a draw from a normally distributed random variable with mean zero and variance again determined by the pedigree and variance components in the ancestral population. It is not affected by conditioning on parental trait values. This segregation of the trait into a shared part and a residual part that is independent for each member of a family is not the classical subdivision into additive and dominance components, but it arises naturally both in the formulation of the infinitesimal model and in its derivation as a limit of Mendelian inheritance for a large number of loci each of small effect. We give a more mathematical description of it in Equation (2).

Our work can be seen as an extension of that of Abney *et al.* (2000), who establish sufficient conditions for a Central Limit Theorem to be applied to the vector of trait values in the presence of dominance and inbreeding. Our second contribution in this work is to establish the magnitude of the error in that normal approximation, verify that in conditioning on the trait values of the parents of an individual we are not (unless those traits are very extreme or the pedigree is very inbred) leaving the domain where the normal approximation is valid, and write down the effect of knowing those parental trait values on the distribution of the individual’s own trait. A careful statement of our results can be found in Theorems 4 and 5. The notation we shall need is rather involved, but in a nutshell, we shall write the trait ${\tilde{Z}}^{i}$ of a given diploid individual *i* in generation *t* as the sum over *M* loci of per-locus allelic effects that are functions of the allelic states $\chi_{l}^{1},\chi_{l}^{2}$ of the two genes of individual *i* at locus *l*, plus an environmental contribution $E^{i}$ (that we shall assume to be Gaussian):


(1) $${\tilde{Z}}^{i}={\bar{z}}_{0}+\sum_{l=1}^{M}\frac{1}{\sqrt{M}}(\eta_{l}(\chi_{l}^{1})+\eta_{l}(\chi_{l}^{2})+\phi_{l}(\chi_{l}^{1},\chi_{l}^{2}))+E^{i}.$$

Here, ${\bar{z}}_{0}$ is the average trait value in the ancestral population (itself a sum of average allelic effects) and the sum encodes the contribution of all loci to the deviation from this average [each per-locus deviation being of order $1/\sqrt{M}$, see Barton *et al.* (2017) and the third section below for a justification]. In this sum, the term $\eta_{l}(\chi_{l}^{1})+\eta_{l}(\chi_{l}^{2})$ models the additive part of the contribution of locus *l* and $\phi_{l}(\chi_{l}^{1},\chi_{l}^{2})$ models the part due to dominance. Assuming Mendelian inheritance and no linkage between the *M* loci, at each locus the allelic state $\chi_{l}^{1}$ is a copy of the allelic state of one of the two genes in the “first” parent of *i*, chosen at random, and $\chi_{l}^{2}$ is a copy of the allelic state of one of the two genes in the “second” parent of *i*, again chosen uniformly at random. Writing $\chi_{l}^{i[1],1},\chi_{l}^{i[1],2}$ for the alleles at locus *l* in the first parent and $\chi_{l}^{i[2],1},\chi_{l}^{i[2],2}$ for the alleles in the second parent, we can then write the sum over all loci in Equation (1) as the sum of an *average* parental contribution (shared by all offspring of these parents), and a residual term of mean zero that encodes the stochasticity of Mendelian inheritance (the *actual* genetic contribution of the parents minus their average contribution). To avoid introducing even more notation, here we simply write $R_{A}^{i}$ and $R_{D}^{i}$ for the parts of the residual due to the additive terms and to the dominance terms respectively. Explicit formulae are given in Equations (24)–(27). Doing so, we obtain


(2) $$\begin{array}{rl} {\tilde{Z}}^{i} & ={\bar{z}}_{0}+\frac{1}{\sqrt{M}}\sum_{l=1}^{M}\{\frac{\eta_{l}(\chi_{l}^{i[1],1})+\eta_{l}(\chi_{l}^{i[1],2})}{2}\\ & \;+\frac{\eta_{l}(\chi_{l}^{i[2],1})+\eta_{l}(\chi_{l}^{i[2],2})}{2}\}\\ & \;+\frac{1}{\sqrt{M}}\sum_{l=1}^{M}\{\frac{\phi_{l}(\chi_{l}^{i[1],1},\chi_{l}^{i[2],1})+\phi_{l}(\chi_{l}^{i[1],2},\chi_{l}^{i[2],1})}{4}\\ & \;+\frac{\phi_{l}(\chi_{l}^{i[1],1},\chi_{l}^{i[2],2})+\phi_{l}(\chi_{l}^{i[1],2},\chi_{l}^{i[2],2})}{4}\}\\ & \;+R_{A}^{i}+R_{D}^{i}+E^{i}\\ & =\;:\;{\bar{z}}_{0}+A^{i}+D^{i}+R_{A}^{i}+R_{D}^{i}+E^{i}. \end{array}$$

The genetic component of the trait can thus be seen either as the sum of an additive part ($A^{i}+R_{A}^{i}$) and a dominance part ($D^{i}+R_{D}^{i}$), or as the sum of a shared part ($A^{i}+D^{i}$) and a residual part ($R_{A}^{i}+R_{D}^{i}$). Following the same strategy as in Barton *et al.* (2017), in Theorem 4 we show that even conditionally on (i.e. knowing) the parental traits ${\tilde{Z}}^{i[1]}$ and ${\tilde{Z}}^{i[2]}$, as *M* tends to infinity the residual part converges in distribution to a Gaussian distribution with mean 0 and a variance depending only on variance components in the ancestral population and on the probability of identity by descent between two parental genes (which is fully determined by the pedigree). Crucially, the limiting variance does not depend on the parental traits. This convergence happens at a rate proportional to $1/\sqrt{M}$. Turning to the shared part, we use a different approach to prove that conditional on ${\tilde{Z}}^{i[1]}$ and ${\tilde{Z}}^{i[2]}$, $A^{i}+D^{i}$ also converges to a Gaussian distribution as *M* tends to infinity. Again, the nonzero mean and the variance of the limiting normal distribution can be fully described, the variance is independent of the parental traits and the convergence happens at a rate proportional to $1/\sqrt{M}$. This is the content of Theorem 5, in the special (and most difficult) case when individual *i* was produced by selfing. For both the shared and the residual parts, the rate of convergence deteriorates when the pedigree is too inbred (leading to probabilities of identity by descent close to 1 between some pairs of parental genes), or when some traits in the population are too extreme (as knowing the trait value then gives us too much information about the unobserved underlying allelic states).

Our derivation of the infinitesimal model as the limit of a finite-locus model has two interesting corollaries. First, as mentioned above, we obtain that the error made by approximating the trait distribution within a family by a Gaussian distribution increases by a quantity of order $1/\sqrt{M}$ in each generation. Consequently, for very large *M*, we expect the infinitesimal model with dominance to be valid for a time of the order of $\sqrt{M}$ generations, provided the population is not too inbred and no too extreme traits appear in the meantime. Second, the set of technical lemmas that are key to the proofs of these results, presented in Appendix E, show that the infinitesimal model leaves essentially no signature on the allele frequencies at any given locus: even knowing the ancestral traits, the distribution of the allelic state at a single locus in a given individual is barely distorted by selection acting on the trait and the result is that, at the population level, the allelic distribution evolves in an essentially neutral way. In particular, its variance only depends on the variance of the allele distribution in the ancestral population and on identities by descent, that are not changed by knowledge of the trait values.

The rest of the paper is organized as follows. In the next section, we define the identity coefficients (that is, probabilities of identity by descent) that we shall need to formulate the model precisely. We show how to compute them knowing the population pedigree in Appendix A and provide the corresponding *Mathematica* code in supplementary material (Barton 2023). Next, we spell out the model in terms of quantities that are familiar from classical quantitative genetics, and we explore its accuracy numerically in a devoted section. Finally, we derive this extension of the infinitesimal model as a limit of a model of Mendelian inheritance on the pedigree. The calculations are somewhat involved, and almost all will be relegated to the appendices. We must modify the strategy of Barton *et al.* (2017), which, although valid for the part of the trait value which is independent for each individual within the family, does not suffice for proving normality of the part of the trait value that is shared by all individuals within a family. To prove that this is normally distributed requires a new approach, based on an extension of Stein’s method of exchangeable pairs. To keep the expressions in our calculations manageable, we satisfy ourselves with presenting the details only in the case in which we condition on knowing the trait values of the parents of an individual, in contrast to the additive case of Barton *et al.* (2017), in which we conditioned on knowing all the trait values in the pedigree right back to the ancestral generation. Our approach could readily be extended to conditioning on knowledge of more trait values, which amounts to conditioning a multivariate normal on some of its marginals. In Appendix H, we present the new ideas that are required to control the way in which errors in the infinitesimal approximation accumulate from knowledge of trait values of more distant relatives in the presence of dominance.

Just as in the additive case, the key will be to show that because many different combinations of allelic states are consistent with the same trait value, knowledge of the pedigree, and the trait values of the parents of an individual in that pedigree, actually gives very little information about the allelic state at a particular locus in that individual, or about correlations between two specific loci. An important consequence of this is that, in practice, it is going to be hard to observe signals of polygenic adaptation, because even a large shift in a trait caused by strong selection does not yield a prediction about alleles at a particular locus.

## Identity coefficients

In the case of an additive trait, the infinitesimal model can be expressed in terms of the variance in the ancestral population (that is, the base population which we shall call *generation zero*) and two-way identity coefficients at a single locus. Recall that two genes at a given locus are identical by descent if their allelic states are identical and were inherited from a common ancestor. Since we assume that individuals are diploid, we need to specify which genes we consider when defining the identity coefficients.

For two distinct individuals *i* and *j* in the same generation, we define $F_{ij}$ to be the probability of identity by descent between two genes (at a given locus), one taken uniformly at random among the two genes of individual *i* and one taken at random among the two genes of individual *j*. When i=j, $F_{ii}$ is defined to be the probability of identity by descent of the two *distinct* genes in the diploid individual *i*.

The definition naturally extends to subsets of three or four genes taken from two distinct individuals (again, at a given locus), for which we shall talk about three- and four-way identities. These quantities will be required to state our results below.

**Fig. 1.**
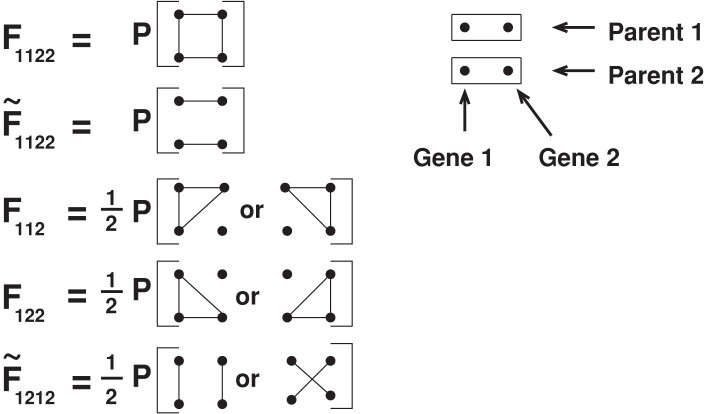
Three- and four-way identities. Lines indicate identity by descent between genes. See the main text for further explanation.

**Fig. B1.**
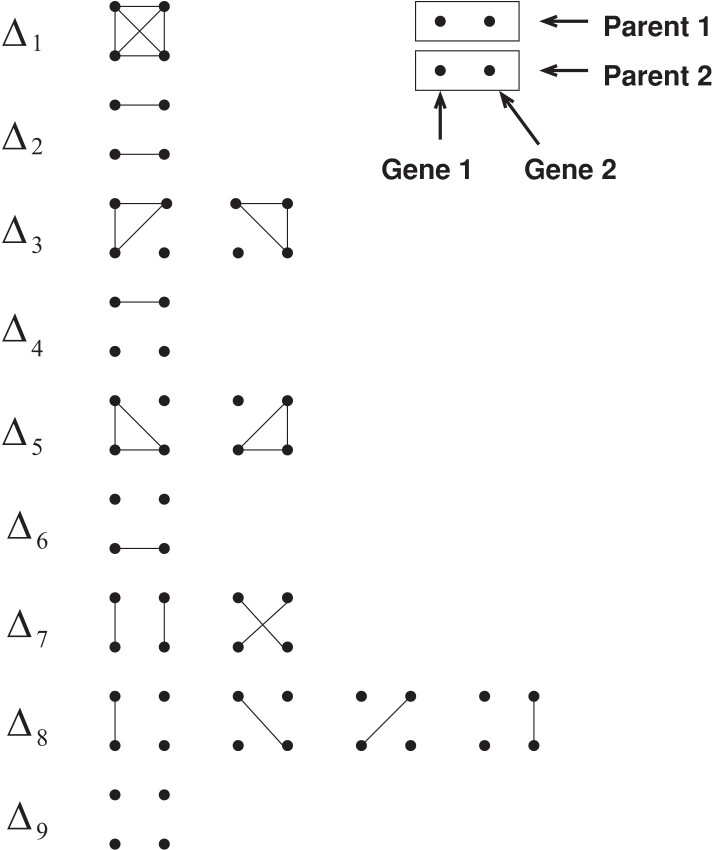
All possible four-way identities. The dots represent the four genes across the two parents (each parent corresponding to a row) and lines indicate identity (c.f. Abney *et al.* 2000).

We use $F_{122}$ for the probability that the two genes in individual 2 are identical by descent *and* they are identical by descent with a gene chosen at random from individual 1. We write $F_{1122}$ for the probability that all four genes across individuals 1 and 2 are identical by descent; this corresponds to the quantity δ in Walsh and Lynch (2018, Chapter 11). We need an expression for the probability that each gene in individual 1 is identical by descent with a *different* gene in individual 2 and all four are not identical. We shall denote this by ${\tilde{F}}_{1212}$. This is denoted by (Δ−δ) in Walsh and Lynch (2018). Finally, we need the probability that the two genes in individual 1 are identical, as are the two genes in individual 2, but the four genes are not *all* identical, which we shall denote by ${\tilde{F}}_{1122}$. We illustrate the three- and four-way identities in Fig. 1. During the course of our mathematical derivations, it will be convenient to express all two-, three-, and four-way identities in terms of the nine possible four-way identities (Walsh and Lynch 2018, Fig. 11.5). This is illustrated in Fig. B1.

In Appendix A, we discuss how to compute these identity coefficients given a pedigree. From now on we simply write “identity” instead of “identity by descent.”

## The infinitesimal model with dominance

For ease of exposition, in this section we leave aside the environmental component of the trait value and we focus on its genetic component, which we denote by *Z* [so that in the notation of Equation (1), $\tilde{Z}=Z+E$]. We first introduce the different quantities that are involved in this component of the trait value in a rigorous way, most of which were already hinted at in the *Introduction*, and then we compute the mean and variance of the shared and residual parts of *Z* with and without knowledge of the parental traits.

The population is diploid and trait values are determined by the allelic states at *M unlinked* loci. Each locus thus corresponds to a pair of genes. We assume that in generation zero (i.e. in the “ancestral” population), the individuals that found the pedigree are unrelated and sampled from an ancestral population in which all loci are in linkage equilibrium and are in Hardy–Weinberg equilibrium (that is, in the ancestral population the two allelic states at each locus in a given individual are sampled independently of each other and therefore the probability that an individual carries a given pair of alleles is given by the product of the probabilities of each allele being sampled).

In order to define the various quantities that enter into our model, we introduce notation to express the trait as a sum of effects over loci. However, we emphasize that once these components, all of which are familiar from classical quantitative genetics, have been calculated for the ancestral population, the model can be defined without reference to the effects of individual loci.

To adhere to the notation of Barton *et al.* (2017), we use $\chi_{l}^{1}$, $\chi_{l}^{2}$ for the allelic states of the two genes at locus *l* in a given individual in the pedigree. When we talk about the distribution of the allelic state of a single gene, we drop the superscript 1 or 2 and simply write $\chi_{l}$. We write ${\bar{z}}_{0}$ for the mean trait value in the ancestral population and express the trait value of an individual as ${\bar{z}}_{0}$ plus a sum of allelic effects. The influence of each locus will scale as $1/\sqrt{M}$, where *M* is the total number of loci (assumed large). We write $\eta_{l}(\chi_{l})$ to denote the (order one) scaled additive effect of the allele $\chi_{l}$ and $\phi_{l}(\chi_{l}^{1},\chi_{l}^{2})$ for the scaled dominance component (where $\phi_{l}$ is assumed to be a symmetric function of the two allelic states $\chi_{l}^{1}$ and $\chi_{l}^{2}$). That is, the total contribution of locus *l* to the trait value will be of the form


$$\frac{1}{\sqrt{M}}(\eta_{l}(\chi_{l}^{1})+\eta_{l}(\chi_{l}^{2}))+\frac{1}{\sqrt{M}}\phi_{l}(\chi_{l}^{1},\chi_{l}^{2}).$$

We shall assume that both $\eta_{l}$ and $\phi_{l}$ are uniformly bounded (i.e. they will all take their values in some finite interval [−B,B]). We also suppose that dominance effects are sufficiently “balanced” that inbreeding depression is finite at least in the ancestral population. More precisely, let ${\hat{\chi }}_{l}$ denote an allele sampled at random from the distribution of alleles at locus *l* in the ancestral population, then ι defined by


(3) $$\iota =\frac{1}{\sqrt{M}}\sum_{l=1}^{M}E[\phi_{l}({\hat{\chi }}_{l},{\hat{\chi }}_{l})]$$

is bounded (as a function of *M*). This condition is crucial to our result. It is not obvious that it can hold, as the number of terms in the sum grows linearly with *M* while the scaling factor $1/\sqrt{M}$ decreases much more slowly. Such a uniform bound is possible for instance if we consider a situation in which the contributions of the different loci compensate each other in a “random-walk-like” way, i.e. each expectation is either positive or negative (by the same amount, say), and the number of positive and negative expectations differ by at most $O(\sqrt{M})$. An example is presented at the beginning of the section on numerics. Note however that the quantity ι may be bounded uniformly in *M* for many other reasons. For simplicity, we do not consider higher order dominance components (that is D×D—or more complex—components) here.

Remark 1 Note that ${\hat{\chi }}_{l}$ is the random variable describing a draw from the distribution of allelic states at locus *l* in the ancestral population (generation 0), while we use $\chi_{l}$ to denote the allelic state at locus *l* in a given individual in the pedigree (living in generation *t*, say). A priori, the law of $\chi_{l}$ is a biased version of the law of ${\hat{\chi }}_{l}$, obtained after letting selection and drift act over *t* generations, but in Appendix E we shall show that, in effect, this distortion is very small for each given locus, and ${\hat{\chi }}_{l}$ and $\chi_{l}$ have the same distribution up to a small error even if we condition on knowing the parental (or ancestral) trait values.

For an individual in the ancestral population, its allelic states at locus *l*, which we denote by ${\hat{\chi }}_{l}^{1},{\hat{\chi }}_{l}^{2}$, are independent draws from a distribution ${\hat{\nu }}_{l}$ on possible allelic states that we assume is known. It is convenient to normalize so that $E[\eta_{l}({\hat{\chi }}_{l})]=0$, $E[\phi_{l}({\hat{\chi }}_{l}^{1},{\hat{\chi }}_{l}^{2})]=0$, and for any value $x^{\prime }$ of the allelic state at locus *l*, the conditional expectations $E[\phi_{l}({\hat{\chi }}_{l},x^{\prime })]=0=E[\phi_{l}(x^{\prime },{\hat{\chi }}_{l})]$. We explain in the section on modeling Mendelian inheritance why these assumptions do not result in a loss of generality. The genetic component of the trait value takes the form [compare with Equation (1), the expression for the observed trait including environmental noise]


(4) $$Z={\bar{z}}_{0}+\frac{1}{\sqrt{M}}\sum_{l=1}^{M}(\eta_{l}(\chi_{l}^{1})+\eta_{l}(\chi_{l}^{2})+\phi_{l}(\chi_{l}^{1},\chi_{l}^{2})).$$

Let us write i[1] and i[2] for the parents of the individual labeled *i*. As advertised in the *Introduction*, the genetic component of an offspring’s trait value has two contributions. The first one is shared by all its siblings, and is a random quantity which is characteristic of the family. The second contribution is unique to the individual and independent of the first one. In our proofs, we shall investigate these two parts separately. We shall use the notation $Z^{i}=(A^{i}+D^{i})+(R_{A}^{i}+R_{D}^{i})$, where the shared part has been further subdivided into the contribution $A^{i}$ from the additive component, and the contribution $D^{i}$ from the dominance component. The residuals $R_{A}^{i}$ and $R_{D}^{i}$ are determined by Mendelian inheritance and correspond to the contributions from the additive and dominance components respectively. Explicit expressions for these quantities are in Equations (24)–(29) below. In this notation, the additive part of the trait value is $A^{i}+R_{A}^{i}$ and the dominance deviation is $D^{i}+R_{D}^{i}$.

### Trait values for a given pedigree

We now define the infinitesimal model in terms of classical quantities of quantitative genetics that can be expressed in terms of expectations in the ancestral population and identities determined by the pedigree. We use the notation of Walsh and Lynch (2018), which we recall in Table 1. Under the infinitesimal model, conditional on the pedigree, the components $\left(A^{i}+D^{i}\right)$ and $\left(R_{A}^{i}+R_{D}^{i}\right)$ of the trait values of individuals in a family follow independent multivariate normal distributions. In Appendix B, the expressions presented in this section will be justified by taking the trait values determined by Equation (4) under a model of Mendelian inheritance. In writing down the infinitesimal model, we shall assume that as the number of loci tends to infinity, the quantities defined in the top part of Table 1 converge to well-defined limits.

**Table 1.** Coefficients of classical quantitative genetics (top) and elements of individual trait decomposition (bottom).

Additive variance	$\sigma_{A}^{2}=\frac{2}{M}\sum_{l=1}^{M}E[\eta_{l}({\hat{\chi }}_{l}{)}^{2}]$
Dominance variance	$\sigma_{D}^{2}=\frac{1}{M}\sum_{l=1}^{M}E[\phi_{l}({\hat{\chi }}_{l}^{1},{\hat{\chi }}_{l}^{2}{)}^{2}]$
Inbreeding depression	$\iota =\frac{1}{\sqrt{M}}\sum_{l=1}^{M}E[\phi_{l}({\hat{\chi }}_{l},{\hat{\chi }}_{l})]$
Sum of squared locus-specific inbreeding depressions	$\iota^{*}=\frac{1}{M}\sum_{l=1}^{M}E[\phi_{l}({\hat{\chi }}_{l},{\hat{\chi }}_{l}){]}^{2}$
Variance of dominance effects in inbred individuals	$\sigma_{DI}^{2}=\frac{1}{M}\sum_{l=1}^{M}(E[\phi_{l}({\hat{\chi }}_{l},{\hat{\chi }}_{l}{)}^{2}]-E[\phi_{l}({\hat{\chi }}_{l},{\hat{\chi }}_{l}){]}^{2})$
Covariance of additive and dominance effects in inbred individuals	$\sigma_{ADI}=\frac{2}{M}\sum_{l=1}^{M}E[\eta_{l}({\hat{\chi }}_{l})\phi_{l}({\hat{\chi }}_{l},{\hat{\chi }}_{l})]$
Additive part of the shared component	$A^{i}$ —defined in Equation (28)
Dominance part of the shared component	$D^{i}$ —defined in Equation (29)
Additive part of the residual	$R_{A}^{i}$ —defined by Equations (24) + (25)
Dominance part of the residual	$R_{D}^{i}$ —defined by Equations (26) + (27)
Genetic component of trait value	$Z^{i}={\bar{z}}_{0}+A^{i}+D^{i}+R_{A}^{i}+R_{D}^{i}$
Observed trait value	${\tilde{Z}}^{i}=Z^{i}+E^{i}$ , $\;E^{i}∼N(0,\sigma_{E}^{2})$

We use ${\hat{\chi }}_{l}$ to denote an allelic state sampled from the distribution ${\hat{\nu }}_{l}$ of possible allelic states at locus *l* in the ancestral population; ${\hat{\chi }}_{l}^{1}$, ${\hat{\chi }}_{l}^{2}$ are independent draws from the same distribution.

To simplify notation, we shall use 1 and 2 in place of i[1] and i[2] in our expressions for identity; thus, for example, $F_{12}\equiv F_{i[1],i[2]}$, and $F_{11}$ will be the probability of identity by descent of the two genes in parent i[1]. The mean and variance of $\left(A^{i}+D^{i}\right)$ are then


(5) $$E[A^{i}+D^{i}]=\iota F_{12},$$

and


(6) $$\begin{array}{rl} & Var(A^{i}+D^{i})\\ & \;=\frac{\sigma_{A}^{2}}{2}\left(1+\frac{F_{11}+F_{22}}{2}+2F_{12}\right)+\sigma_{ADI}\left(F_{12}+\frac{F_{112}+F_{122}}{2}\right)\\ & \;+\frac{\left(\sigma_{DI}^{2}+\iota^{*}\right)}{4}(F_{12}+F_{112}+F_{122}+F_{1122})+\frac{\iota^{*}}{4}{\tilde{F}}_{1212}-\iota^{*}F_{12}^{2}\\ & \;+\frac{\sigma_{D}^{2}}{4}\left(1-F_{12}+F_{22}-F_{122}+F_{11}-F_{112}+{\tilde{F}}_{1122}+\frac{1}{2}{\tilde{F}}_{1212}\right). \end{array}$$

In this expression, the term proportional to $\sigma_{A}^{2}$ is the variance of $A^{i}$, the term proportional to $\sigma_{ADI}$ is twice the covariance of $A^{i}$ and $D^{i}$ and the remaining sum gives the variance of $D^{i}$. Recall that we are assuming here that the ancestral population is in linkage equilibrium. With linkage disequilibrium there is an additional term, c.f. the remark below Equation (11). The components (A+D) are also correlated across families. For individuals labeled *i* and *j*, respectively,


(7) $$\begin{array}{rl} & Cov((A^{i}+D^{i}),(A^{j}+D^{j}))\\ & \;=2F_{ij}\sigma_{A}^{2}+(F_{ijj}+F_{iij})\sigma_{ADI}\\ & \;+{\tilde{F}}_{ijij}\sigma_{D}^{2}+F_{iijj}(\sigma_{DI}^{2}+\iota^{*})-\iota^{2}F_{ii}F_{\;jj}+\iota^{*}{\tilde{F}}_{iijj}. \end{array}$$

Note that, in contrast to our expression for the variance of $Z^{i}$, in this expression, the subscripts *i* and *j* in the identities refer to the individuals themselves, not their parents; for example, the expression $F_{ij}$ is the probability of identity of two genes, one sampled at random from individual *i* and one sampled at random from individual *j*. We reserve letters for individuals in the current generation, and numbers for their parents.

If we combine the components $R_{A}^{i}$ and $R_{D}^{i}$ that segregate within families, we have that the sums $\left(R_{A}^{i}+R_{D}^{i}\right)$ are independent of each other (due to the independence of the variables encoding Mendelian inheritance), mean zero, normally distributed random variables with variance


(8) Var(RAi+RDi)=(1−F11+F222)σA22+14(3F12−F1122−F112−F122)(σDI2+ι*)+14[3(1−F12)−(F11−F112)−(F22−F122)−F~112212−12F~1212]σD2+(F12−F112+F1222)σADI−ι*4F~1212.

Here again, the term proportional to $\sigma_{A}^{2}$ is the variance of $R_{A}^{i}$, the term proportional to $\sigma_{ADI}$ is twice the covariance of $R_{A}^{i}$ and $R_{D}^{i}$, and the remaining sum equals the variance of $R_{D}^{i}$. We calculate the mean, variance, and covariance of these different components in Appendix B. In order to recover the mean and variance of the trait values, we add the contributions of $\left(A^{i}+D^{i}\right)$ and $\left(R_{A}^{i}+R_{D}^{i}\right)$ and observe that the identity $F_{12}$ in our expressions for the variances of these quantities (which we recall was the probability of identity of one gene sampled at random from each of the parents i[1], i[2] of our individual) corresponds to $F_{ii}$. This yields that, conditional on the pedigree,


(9) $$\begin{array}{rl} E[Z^{i}] & ={\bar{z}}_{0}+\iota F_{ii}, \end{array}$$

(10) $$\begin{array}{rl} Cov(Z^{i},Z^{j}) & =2F_{ij}\sigma_{A}^{2}+(F_{ijj}+F_{iij})\sigma_{ADI}+{\tilde{F}}_{ijij}\sigma_{D}^{2}\\ & \;+F_{iijj}\;(\sigma_{DI}^{2}+\iota^{*})-\iota^{2}F_{ii}F_{\;jj}+\iota^{*}{\tilde{F}}_{iijj}, \end{array}$$

and


(11) $$\begin{array}{rl} Var(Z^{i}) & =\sigma_{A}^{2}(1+F_{ii})+\sigma_{D}^{2}(1-F_{ii})+(\sigma_{DI}^{2}+\iota^{*})F_{ii}\\ & \;+2\sigma_{ADI}F_{ii}-\iota^{*}F_{ii}^{2}. \end{array}$$

For a single individual, its trait value can only depend on the two alleles that it carries at each locus, so it is no surprise that this expression depends only on pairwise identities between those two genes. We remark that Equation (11) differs from the corresponding expression [Equation (11.6c) in Walsh and Lynch (2018)]. To recover exactly their expression, one must add $\left(\tilde{\;f}-F_{ii}^{2})(\iota^{2}-\iota^{*}\right)$ to the right-hand side, where $\tilde{f}$ is the probability of identity at two distinct loci in individual *i*. We see how to recover this term in Remark B1, but because we have assumed linkage equilibrium in our base population, for the period over which the infinitesimal model remains a good approximation, under our assumptions we have $\tilde{f}\approx F_{ii}^{2}$. This is not to say that there is not a significant contribution to the trait value from linkage disequilibrium; it is just that for any specific pair of loci it is negligible. We shall see a toy example that reinforces this point at the beginning of the section on modeling Mendelian inheritance.

We emphasize again that our partition of the trait values into a contribution that is shared by all individuals in a family and residuals differs from the conventional split into an additive part and a dominance deviation. The additive part of the trait is $A^{i}=A^{i}+R_{A}^{i}$ and the dominance component is $D^{i}=D^{i}+R_{D}^{i}$. From our calculations in Appendix B, we can read off


(12) $$E[A^{i}]=0,\;E[D^{i}]=\iota F_{ii},$$

(13) $$Var(A^{i})=\sigma_{A}^{2}(1+F_{ii}),\;Cov(A^{i},D^{i})=\sigma_{ADI}F_{ii},$$

and


(14) $$Var(D^{i})=\sigma_{D}^{2}(1-F_{ii})+\sigma_{DI}^{2}F_{ii}+\iota^{*}(F_{ii}-F_{ii}^{2}).$$

Remark 2 Notice that the purely additive case can be simply recovered by taking $\phi_{l}\equiv 0$, so that $D^{i}=0=R_{D}^{i}$, and $\sigma_{A}^{2}$ is the only nonzero variance coefficient. This yields

$$\begin{array}{rl} & E[A^{i}+D^{i}]=0,\;Var(A^{i}+D^{i})=\frac{\sigma_{A}^{2}}{2}\left(1+\frac{F_{11}+F_{22}}{2}+2F_{12}\right),\\ & Cov((A^{i}+D^{i}),(A^{j}+D^{j}))=2F_{ij}\sigma_{A}^{2},\\ & Var(R_{A}^{i}+R_{D}^{i})=\left(1-\frac{F_{11}+F_{22}}{2}\right)\frac{\sigma_{A}^{2}}{2}, \end{array}$$

and finally

$$E[Z^{i}]={\bar{z}}_{0},\;Var(Z^{i})=\sigma_{A}^{2}(1+F_{ii}),\;Cov(Z^{i},Z^{j})=2F_{ij}\sigma_{A}^{2}.$$

### Conditioning on trait values of parents

Under the infinitesimal model, the trait values of individuals across the pedigree are given by a multivariate normal. Therefore, standard results on conditioning multivariate normal random vectors on their marginal values, which for ease of reference we record in Appendix C, allow us to read off the effect on the distribution of $Z^{i}$ of conditioning on $Z^{i[1]}$ and $Z^{i[2]}$. However, a little care is needed; we shall be justifying the normal distribution within families as an approximation as the number of loci tends to infinity, and we must be sure that asymptotic normality is preserved under this conditioning. We shall see that if, for example, parental trait values are too extreme, then the conditioning pushes us to a part of the probability space where the normal approximation breaks down. This is particularly evident in the toy example that we present in the section on modeling Mendelian inheritance. A justification for asymptotic normality even after conditioning is outlined in that section, and details are presented in the appendices.

Just as in the classical infinitesimal model, the mean and variance of the residuals $R_{A}^{i}+R_{D}^{i}$ are unchanged by conditioning on the trait values of the parents [recall that these residuals encode the stochasticity due to Mendelian inheritance at each locus; expressions for $R_{A}^{i}$ and $R_{D}^{i}$ are given in Equations (24)–(27)]. For the shared components, the mean and variance will be distorted by quantities determined by the covariances between $\left(A^{i}+D^{i}\right)$ and $Z^{i[1]}$, $Z^{i[2]}$. Let us write


(15) $$C(i,i[1])\;:\;=Cov((A^{i}+D^{i}),Z^{i[1]}),$$

with a corresponding definition for C(i,i[2]). Then, once again using 1 and 2 in place of i[1] and i[2] in our expressions for identities,


(16) $$\begin{array}{rl} C(i,i[1]) & =\frac{\sigma_{A}^{2}}{2}(1+F_{11}+2F_{12})+\frac{\sigma_{ADI}}{2}(F_{11}+F_{12}+2F_{112})\\ & \;+\sigma_{D}^{2}(F_{12}-F_{112})+(\sigma_{DI}^{2}+\iota^{*})F_{112}-\iota^{2}F_{11}F_{12}, \end{array}$$

with C(i,i[2]) given by the corresponding expression with the roles of the subscripts 1 and 2 interchanged. (A derivation of this expression is provided in Appendix B.) With this notation,


(17) $$\begin{array}{rl} & E[(A^{i}+D^{i})|Z^{i[1]},Z^{i[2]}]\\ & \;=E[(A^{i}+D^{i})]+\frac{1}{Var(Z^{i[1]})\;Var(Z^{i[2]})-Cov(Z^{i[1]},Z^{i[2]}{)}^{2}}\\ & \;\times \left\{(C(i,i[1])\;Var(Z^{i[2]})-C(i,i[2])\;Cov(Z^{i[1]},Z^{i[2]})\right)\\ & \;\times (Z^{i[1]}-E[Z^{i[1]}])\\ & \;+(C(i,i[2])\;Var(Z^{i[1]})-C(i,i[1])\;Cov(Z^{i[1]},Z^{i[2]}))\\ & \;\times (Z^{i[2]}-E[Z^{i[2]}])\}, \end{array}$$

and


(18) $$\begin{array}{rl} & Var((A^{i}+D^{i})|Z^{i[1]},Z^{i[2]})\\ & \;=Var(A^{i}+D^{i})\\ & \;-\frac{Var(Z^{i[1]})C(i,i[2]{)}^{2}+Var(Z^{i[2]})C(i,i[1]{)}^{2}}{Var(Z^{i[1]})\;Var(Z^{i[2]})-Cov(Z^{i[1]},Z^{i[2]}{)}^{2}}\\ & \;+\frac{2Cov(Z^{i[1]},Z^{i[2]})C(i,i[1])C(i,i[2])}{Var(Z^{i[1]})\;Var(Z^{i[2]})-Cov(Z^{i[1]},Z^{i[2]}{)}^{2}}. \end{array}$$

(We have implicitly assumed that i[1]≠i[2]; in the case i[1]=i[2] the expression is simpler as we are then conditioning a bivariate normal on one of its marginals.)

Remark 3 In the purely additive case, things simplify greatly. From the expressions above, before conditioning, the mean of $A^{i}+D^{i}$ is zero (since ι=0), and the variance is

$$\frac{\sigma_{A}^{2}}{2}\left(1+\frac{\left(F_{11}+F_{22}\right)}{2}+2F_{12}\right).$$

Moreover,

$$\begin{array}{rl} & Var(Z^{i[1]})=\sigma_{A}^{2}(1+F_{11}),\;Var(Z^{i[2]})=\sigma_{A}^{2}(1+F_{22}),\\ & Cov(Z^{i[1]},Z^{i[2]})=2\sigma_{A}^{2}F_{12}, \end{array}$$

and

$$\begin{array}{rl} & C(i,i[1])=\frac{1}{2}\sigma_{A}^{2}(1+F_{11}+2F_{12}),\\ & C(i,i[2])=\frac{1}{2}\sigma_{A}^{2}(1+F_{22}+2F_{12}). \end{array}$$

Substituting into Equations (17) and (18), and observing that

$$\begin{array}{rl} & (1+F_{11})(1+F_{22}+2F_{12}{)}^{2}+(1+F_{22})(1+F_{11}+2F_{12}{)}^{2}\\ & \;-4F_{12}(1+F_{11}+2F_{12})(1+F_{22}+2F_{12})\\ & \;=2((1+F_{11})(1+F_{22})-4F_{12}^{2})\left(1+\frac{F_{11}+F_{22}}{2}+2F_{12}\right), \end{array}$$

we find that conditional on the trait values of the parents, the mean and variance of $A^{i}+D^{i}$ reduce to $(Z^{i[1]}+Z^{i[2]})/2$ and zero, respectively, and we recover the classical infinitesimal model.

Although in the presence of dominance the expressions (17) and (18) are rather complicated, we emphasize that they are derived from knowledge of just the ancestral population and the pedigree, and are expressed in terms of familiar quantities from classical quantitative genetics.

## Numerical examples

In this section, we present numerical examples to illustrate the accuracy of the predictions of the infinitesimal model, again disregarding the environmental component of the trait.

We first generated a pedigree for a population of constant size of N=30 diploid individuals over 50 discrete generations. Mating is random, but with no selfing. In order to facilitate comparison of different scenarios, the same pedigree was used for all subsequent simulations. In this way, the identity coefficients are held constant. As expected, the mean probability of identity between pairs of genes sampled from different individuals in generation *t* is close to $1-(1-1/2N{)}^{t}$.

We define a trait, *Z*, which depends on M=1,000 bi-allelic loci. There is no epistasis, so that the trait value is a sum across loci. In the examples here, we assume complete dominance, so that the effects of the three genotypes at each locus are either −α:−α:+α or −α:+α:+α. In order to ensure that the inbreeding depression ι is bounded, we need to have some “balance” and so we choose the effects at each locus according to an independent Bernoulli random variable with parameter *H*; that is, the probability that the effects across the three genotypes at locus *l* is −α:−α:+α is 1−H, independently for each locus. The effect size α is taken to be $1/\sqrt{M}$ for all loci and $H=\frac{1}{2}+\frac{2}{\sqrt{M}}$. With these choices the additive and dominance variances will be O(1).

In the ancestral population, the allele frequencies were generated to mimic neutral allele frequencies with very low mutation rates, but conditioned to segregate at each locus. Thus, allele frequencies at every locus were sampled independently and according to a distribution with density proportional to $(p(1-p){)}^{1-\epsilon }$, with ϵ=0.001, but with those in [0,1/60] and [1−1/60,1] discarded (and the distribution renormalized). Then for each population replicate, these frequencies were used to endow each individual in the base population with an allelic type at every locus.

Variance components are defined with respect to this reference set of allele frequencies. For the population generated for the examples presented here, these values were $\sigma_{A}^{2}=0.269$, $\sigma_{D}^{2}=0.073$, and the inbreeding depression ι=−0.531. The additive and dominance components are uncorrelated in the base population (Cov(A,D)=0). In the numerical experiments that follow, each replicate population is started at time zero from a different collection of genotypes, sampled from this base distribution.

**Fig. 2.**
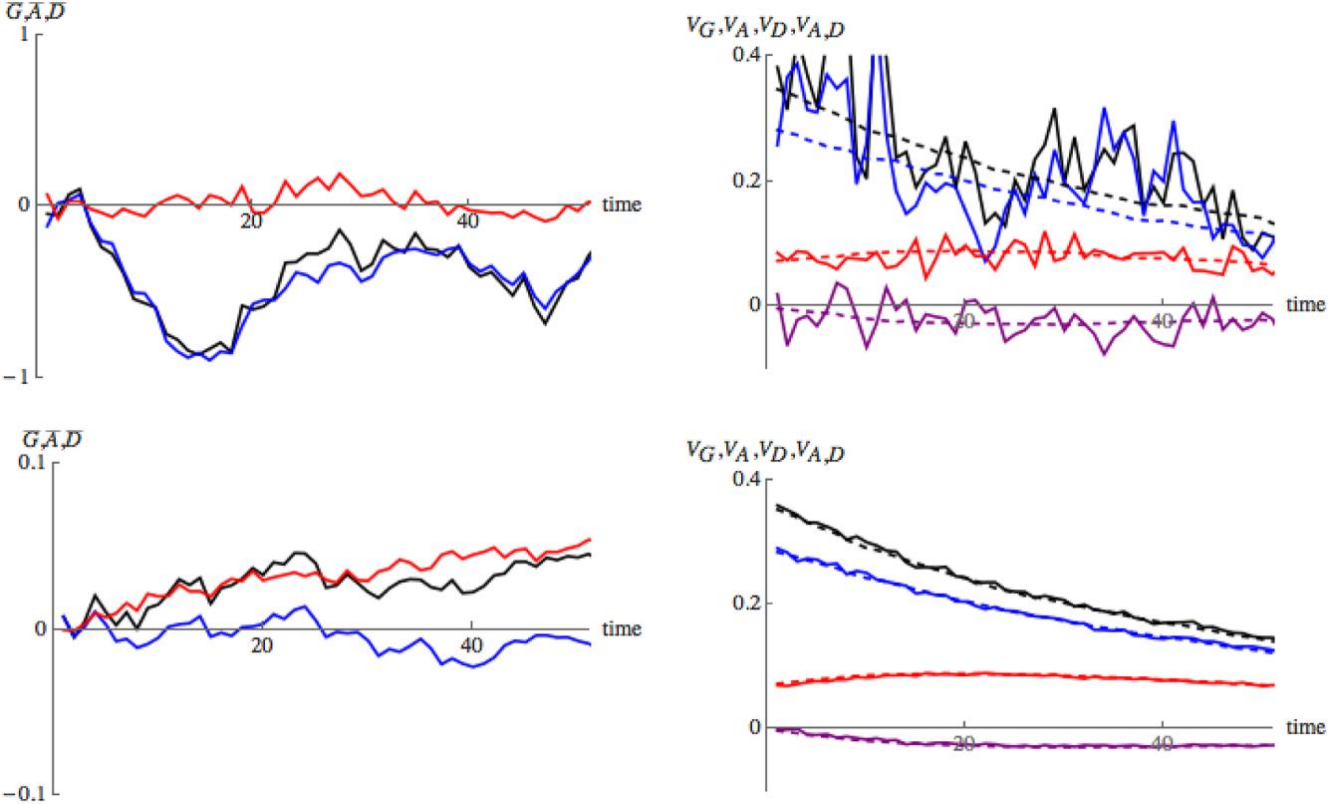
Changes of the mean and variance of the additive part of the trait, the dominance part, and their sum over 50 generations of neutral evolution. The top row shows a single replicate, while the bottom row shows the average over 300 replicates using the same sequence of individuals spanning the 50 generations. The left column shows the means ($\bar{G}=\bar{A}+\bar{D}$, $\bar{A}$, $\bar{D}$; black, or middle curve; blue, or bottom curve; red, or top curve), while the right column shows the variance components ($V_{G}=Var(G)$, $V_{A}=Var(A)$, $V_{D}=Var(D)$, $V_{A,D}=Cov(A,D)$; black, or top curve; blue, or middle top curve; red, or middle bottom curve; purple, or bottom curve). On the right, solid lines show the total variances and covariance, while the dashed lines show the genic component. These differ through the contribution of linkage disequilibrium, which generates substantial variation. The genic component changes smoothly, as expected with a large number (M=1,000) of loci. With M=1,000 loci, we expect the infinitesimal model to be accurate for about $\sqrt{M}∼30$ generations. Simulations are made on a single pedigree with 30 individuals; variance components are measured relative to the ancestral population. The predicted values for these means and variances under the infinitesimal model are given in Equations (12)–(14) (note that the identity coefficients $F_{ii}$ increase through time due to genetic drift).

We first simulated a neutral model. Figure 2 illustrates how the different components of the trait values change over fifty generations of neutral evolution. Recall that we always use the same realization of the pedigree. For each replicate, we take an independent sample of allelic types at time zero. For each individual in the pedigree we evaluate the additive and dominance components *A* and *D* and then in each generation we calculate the mean and variance of these quantities across the 30 individuals in the population. This is only intended to give some feeling for the ways in which the components fluctuate through time. Of course the infinitesimal model is only providing a prediction for the *distribution* of trait values within families; a single realization will see substantial contributions to trait values from linkage disequilibrium (c.f. the toy example in the section on modeling Mendelian inheritance and Theorem 5). In the following figures, we compare these quantities to the detailed predictions of the infinitesimal model. The top row in Fig. 2 is a single replicate, while the bottom is the average over 300 replicates. On the left, we have the mean of the additive and dominance components and their sum; on the right, we have plotted the variance components. For a single replicate, there is indeed a substantial contribution from linkage disequilibrium. When we plot just the genic components (that is the sum over variances at each locus, ignoring the contribution from linkage disequilibrium), as expected, the picture is much smoother and we see that the predictions of the infinitesimal model are close to the values obtained by averaging over 300 replicates. Since linkage disequilibrium will dissipate rapidly, halving in each generation, it is the genic component that determines the long term evolution.

All components are measured relative to the base population. In practice, in natural populations, one does not have access to the ancestral population and so one measures components relative to the current population. This amounts to a change of reference Hill *et al.* (2006). We do not do this in our setting, as it would result in different variance components for every replicate.

**Fig. 3.**
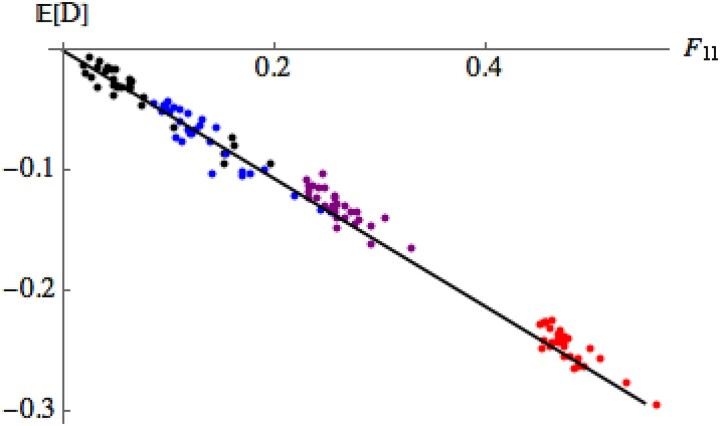
The relation between the dominance deviation and the probability of identity of the two genes within an individual. There is one point for the average over 1,000 replicates for each of the 30 individuals in generations 5, 10, 20, 40 (black, or left-most group of points; blue, or second left-most group; purple, or second right-most group; red, or right-most group). (Recall that the pedigree is fixed, so identities are the same for each replicate.) The mean of *D* decreases as $\iota F_{ii}=-0.53F_{ii}$ (solid line), in accordance with Equation (12).

In Fig. 3, we explore the relationship between the dominance deviation and inbreeding. Since we use the same pedigree for all our experiments, each individual is characterized by a single $F_{ii}$ (the probability of identity of the two genes at a given locus). For each of 1,000 replicates (that is independent samples of allelic types for the individuals in generation zero), we calculated the dominance deviation for each individual in the pedigree. The plot in Fig. 3 shows the dominance deviation averaged over those 1,000 replicates for each individual in the pedigree. Thus, there are 30 points in each generation, one for each individual in the population. As expected, the mean of the dominance component decreases in proportion to $F_{ii}$, $E[D]=-0.53F_{ii}$ (recall that ι=−0.53 for our base population).

**Fig. 4.**
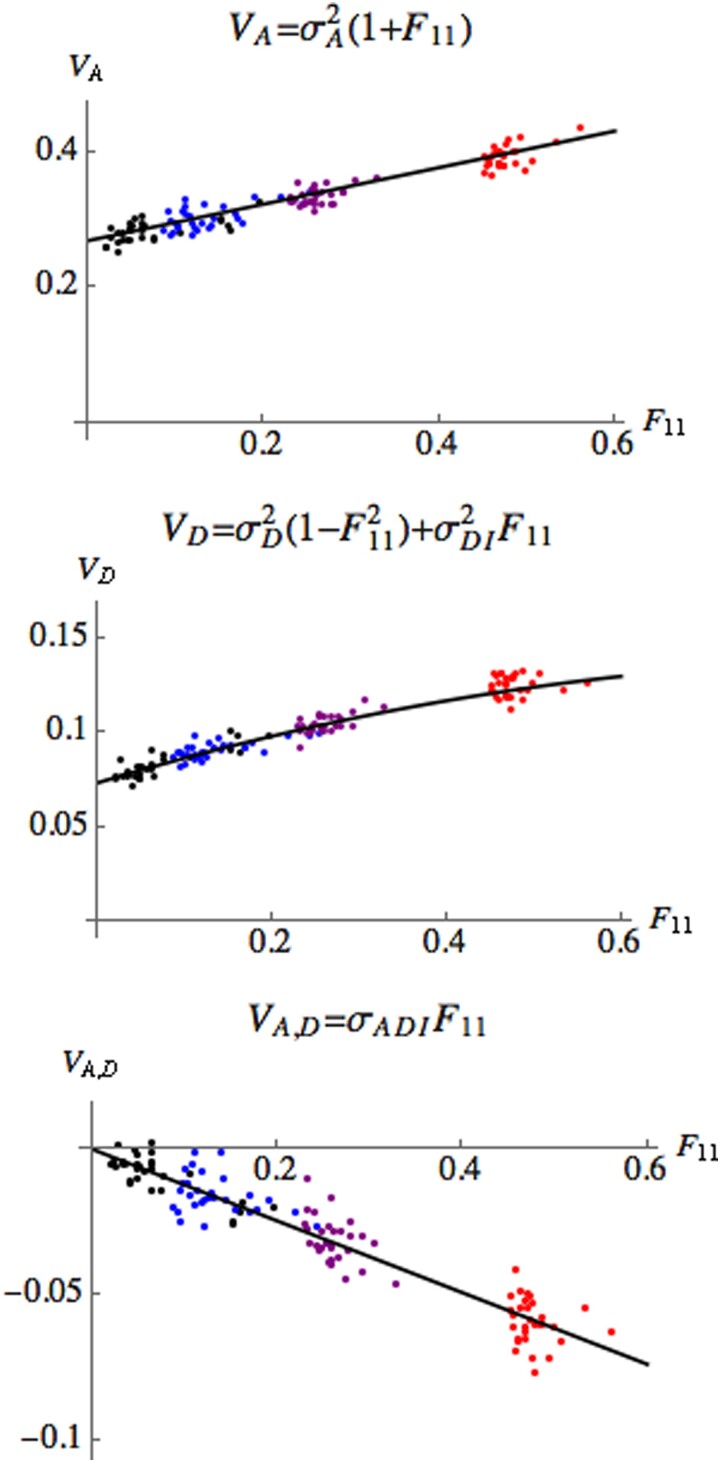
The variance and covariance of *A* and *D* versus identity $F_{ii}$ for individuals in the pedigree. As in Fig. 3, there are 30 points in each generation, one corresponding to each of the 30 individuals in the population. Generations 5, 10, 20, 40 (black, or left-most group of points; blue, or second left-most group; purple, or second right-most group; red, or right-most group). Here, again we use the shorter notation $V_{A}=Var(A)$, $V_{D}=Var(D)$, $V_{A,D}=Cov(A,D)$ and the theoretical predictions were derived in Equations (13) and (14).

Figure 4 shows how the (co)variance of *A* and *D* depends on identity $F_{ii}$ for pairs of individuals in the pedigree. As in Fig. 3, for each individual in the pedigree, *A* and *D* are calculated for each of the 1,000 replicates; Fig. 4 shows the variances and covariances of the resulting values for each of the 30 individuals in generations 5, 10, 20, and 40 and these are compared to the theoretical predictions. Note that since in the bi-allelic case $\sigma_{D}^{2}=\iota^{*}$, the expression (14) for the variance of the dominance component reduces to


$$\sigma_{D}^{2}(1-F_{ii}^{2})+\sigma_{DI}^{2}F_{ii}.$$

**Fig. 5.**
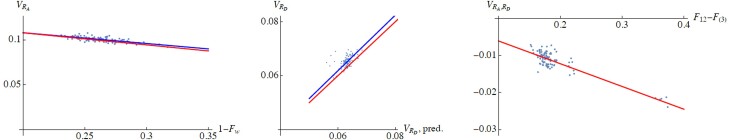
The variance and covariance within families between the residual additive and dominance deviations $R_{A}$ and $R_{D}$ ($V_{R_{A}}=Var(R_{A})$, $V_{R_{D}}=Var(R_{D})$, $V_{R_{A},R_{D}}=Cov(R_{A},R_{D})$). One hundred pairs of parents were chosen at random from the ancestral population and from each one thousand offspring were generated. The within-family variances obtained in this way were averaged over 10 replicates (with the same pedigree and parents). Each of the 100 points in each plot corresponds to one pair of parents. The five outliers are families produced by selfing. The blue lines (or top lines) show a least-squares regression; the red lines (or bottom lines) are the theoretical predictions [see Equation (8)]. The two lines exactly coincide in the plot on the right.

Next, we consider the variances of the residuals $R_{A}$ and $R_{D}$ within families. One hundred pairs of parents were chosen at random from the population, and from each 1,000 offspring were generated. This was repeated for 10 replicates made with the same pedigree and the same set of parents; within-family variances were then averaged over replicates. In Fig. 5, in each plot there are 100 points, one for each pair of parents. The two lines correspond to least square regression (blue) and theoretical predictions (red) which can be read off from Equation (8). For readability, in the figure we use the notation $V_{R_{A}}$, $V_{R_{D}}$, and $V_{R_{A},R_{D}}$ to denote the variance of $R_{A}$, the variance of $R_{D}$ and the covariance between $R_{A}$ and $R_{D}$, respectively. Using Equation (8) and the explanation below, together with the fact that $\sigma_{D}^{2}=\iota^{*}$ in our bi-allelic case, we have $V_{R_{A}}=\sigma_{A}^{2}(1-F_{W})/2$, where $F_{W}=(F_{i[1]i[1]}+F_{i[2]i[2]})/2$ is the within-individual identity averaged over parents 1 and 2;


$$\begin{array}{rl} V_{R_{D}} & =\frac{\sigma_{DI}^{2}}{4}(3F_{12}-F_{1122}-F_{112}-F_{122})\\ & \;+\frac{\sigma_{D}^{2}}{4}\left(3-F_{11}-F_{22}-F_{1122}-{\tilde{F}}_{1122}-\frac{3}{2}{\tilde{F}}_{1212}\right); \end{array}$$

and


$$V_{R_{A},R_{D}}=\frac{\sigma_{ADI}}{2}(F_{12}-F_{\left(3\right)}),$$

where $F_{\left(3\right)}$ is defined as follows:


$$F_{\left(3\right)}=\frac{F_{112}+F_{122}}{2}.$$

**Fig. 6.**
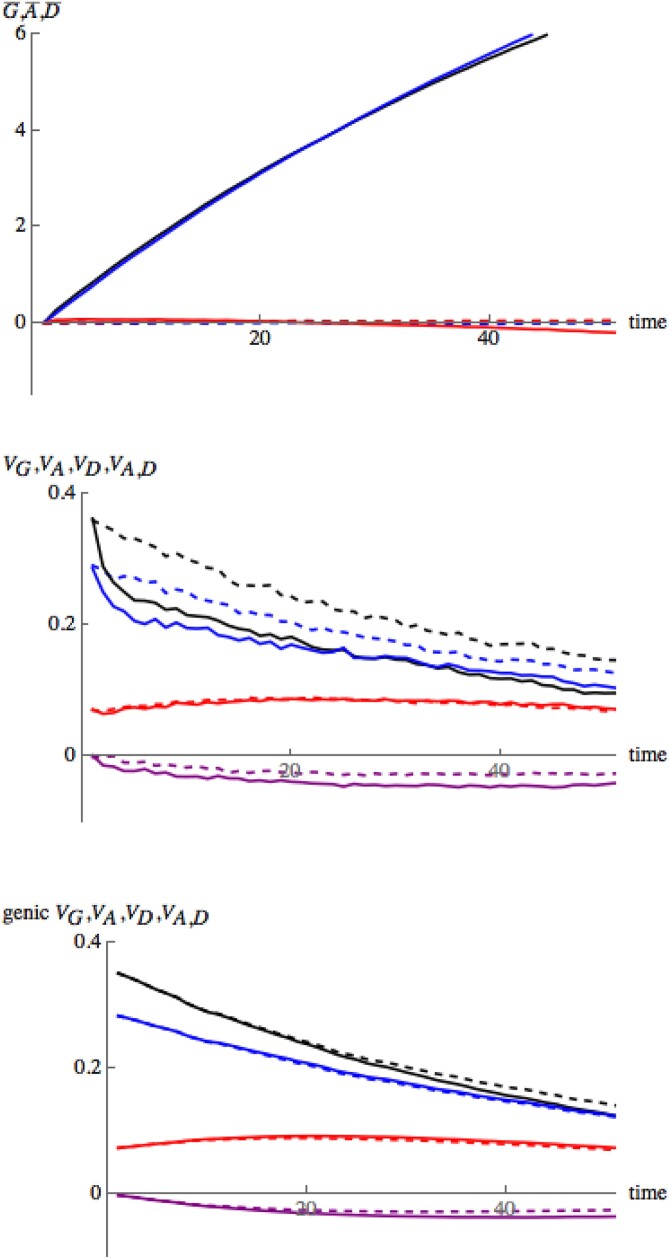
Comparison between a neutral population (dashed lines) and one subject to truncation selection (solid lines). Top row: change in means relative to the initial value ($\bar{G}=\bar{A+D}$, $\bar{A}$,$\bar{D}$; black, or top curves; blue, or middle curves; red, or bottom curves); middle: variances, including linkage disequilibria (top to bottom: $V_{G}=Var(A+D)$, $V_{A}=Var(A)$, $V_{D}=Var(D)$, $V_{A,D}=Cov(A,D)$; black, blue, red, purple). The bottom row is the changes to genic variances with time against predictions of the infinitesimal model. The values are averages over 300 replicates for the neutral case, 1,000 for the selected case, made with the same pedigree. There are M=1,000 loci, and thus we expect the infinitesimal model to be accurate for about $\sqrt{M}∼30$ generations. Selection is made within families; for each offspring, two individuals are generated from the corresponding parents, and the one with the larger trait value retained.

The full force of our theoretical results is that even if we condition on the trait values of parents, the within-family distribution of their offspring will consist of two normally distributed components and, in particular, the variance components will be independent of the trait values of the parents. We test this by imposing strong truncation selection on the population. We retain the same pedigree relatedness, but working down the pedigree, each individual’s genotype is determined by generating two possible offspring from its parents and retaining the one with the larger trait value. In Fig. 6, we compare the results with simulations of the neutral population. Dashed lines are for the neutral simulations, solid ones for the simulation with selection. For the population under selection, we see an immediate drop in the total genetic variation, caused by the strong selection; there is significant negative linkage disequilibrium between individual loci, as predicted by Bulmer (1971). The blue is the additive component. We see that about one-third of the variance is dominance variance. The bottom row shows that the genic components are hardly affected by selection, as predicted by the infinitesimal model. With or without selection, the variance components change as a result of inbreeding.

**Fig. 7.**
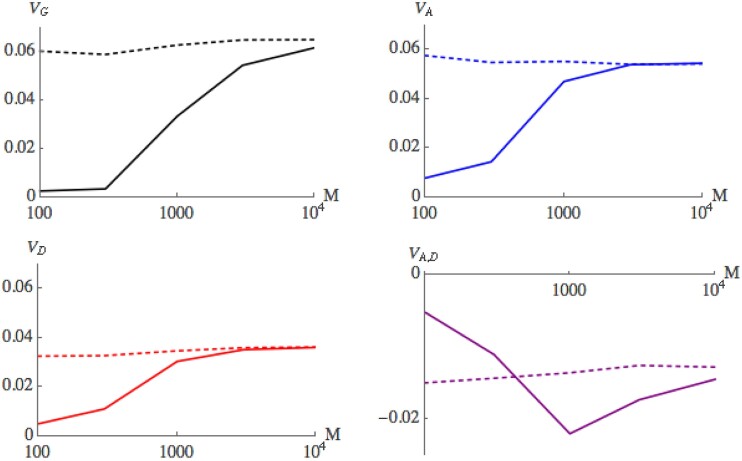
Convergence of the variance components at 50 generations, as the number of loci increases from M=100 to $M=10^{4}$ (same notation as in Fig. 6). Simulations with 50% truncation selection are compared with neutral simulations (solid, dashed lines). The replicate simulations were generated as in Fig. 6 (see main text). Regressions of the log absolute difference between selected and neutral variance components against ln(M) have slopes −0.62, −0.72, −0.70, −0.66 for $V_{G}$, $V_{A}$, $V_{D}$, $V_{A,D}$, respectively (see supplementary material for details). Thus, convergence is somewhat faster than $\sqrt{M}$.

Finally, Fig. 7 compares the variance components at 50 generations for neutral simulations with those with truncation selection as the number of loci increases from M=100 to $M=10^{4}$. Replicate simulations were generated as in Fig. 6. Under the infinitesimal model, these components should take the same values with and without selection. This is reflected in the simulations, with the covariance between the additive and dominance effects being the slowest to settle down to the infinitesimal limit.

## The infinitesimal model with dominance as a limit of Mendelian inheritance

In this section, we turn to the justification of our model as a limit of a model of Mendelian inheritance as the number *M* of loci tends to infinity. Although we shall focus on the distribution of the genetic components of the trait values in the pedigree, in this section we consider the general situation where the *observed* trait of an individual, ${\tilde{Z}}^{i}$, is the sum of a genetic component $Z^{i}$ and an environmental component $E^{i}$. Our mathematical assumptions on $E^{i}$ are detailed in *Main results* below.

Our work is an extension of that of Abney *et al.* (2000), which in turn builds on Lange (1978). The distinctions here are that we explicitly model the component of the trait value that is shared by all individuals in a family separately from the part that segregates within that family; we identify the effect on each of these components of conditioning on knowing the trait values of the parents of the family; and we estimate the error that we are making in taking the normal approximation, thus providing information on when the infinitesimal approximation breaks down.

The fact that the genetic component of trait values within families is normally distributed is a consequence of the Central Limit Theorem. That this remains valid even when we condition on the trait values of the parents stems from the fact that knowing the trait value of an individual actually provides very little information about the allelic state at any particular locus. This in turn is because, typically, there are a large number of different genotypes that are consistent with a given phenotype. In Barton *et al.* (2017), this was illustrated through a simple example which can be found on p. 402 of Fisher (1918), which concerned an additive trait in a haploid population. Here we adapt that example to the model for which we performed our numerical experiments.

Suppose then that we have *M* bi-allelic loci. We denote the alleles at locus *l* by $a_{l}$ and $A_{l}$. The contributions to the trait of the three genotypes $a_{l}a_{l}$, $a_{l}A_{l}$ and $A_{l}A_{l}$ are −α, −α, α respectively with probability $\frac{1}{2}-\frac{2}{\sqrt{M}}$ and they are −α, α, α with probability $\frac{1}{2}+\frac{2}{\sqrt{M}}$. The effect size $\alpha =1/\sqrt{M}$. For simplicity, in contrast to our numerical experiments, we suppose that the probabilities of genotypes $a_{l}a_{l}$, $a_{l}A_{l}$, $A_{l}A_{l}$ are 1/4, 1/2, 1/4 respectively.

Now suppose that we observe the trait value to be $k/\sqrt{M}$. What is the conditional probability that the allelic types at locus *l*, which we denote $\chi_{l}^{1}\chi_{l}^{2}$ are $A_{l}A_{l}$? For definiteness, we take *M* and *k* both to be even and l=1.

First consider the probability that the contribution to the trait value from locus 1 is $+1/\sqrt{M}$. Let us write $p_{+}$ for the (unconditional) probability that the contribution from locus 1 is $1/\sqrt{M}$, that is


$$p_{+}=\frac{1}{4}+\frac{1}{2}\left(\frac{1}{2}+\frac{2}{\sqrt{M}}\right)=\frac{1}{2}\left(1+\frac{1}{\sqrt{M}}\right),$$

and $p_{-}=1-p_{+}$. Let us write $\Psi_{l}/\sqrt{M}$ for the contribution to the trait from locus *l*. We have


P[∑l=1MΨl=k|Ψ1=1]P[∑l=1MΨl=k]=P[∑l=2MΨl=k−1]P[∑l=1MΨl=k]=p+(M+k−2)/2p−(M−k)/2p+(M+k)/2p−(M−k)/2(M−1(M+k−2)/2)(M(M+k)/2)=(1+kM)12p+=(1+kM)1(1+1/M).

An application of Bayes’ rule then gives


$$\begin{array}{rl} & P\left[\chi_{1}^{1}=A_{1},\chi_{1}^{2}=A_{1}|\;\sum_{l=1}^{M}\frac{\Psi_{l}}{\sqrt{M}}=\frac{k}{\sqrt{M}}\right]\\ & \;=\frac{P[\sum_{l=1}^{M}\Psi_{l}=k\;|\;\Psi_{1}=1]}{P[\sum_{l=1}^{M}\Psi_{l}=k]}P[\chi_{1}^{1}=A_{1},\chi_{1}^{2}=A_{1}]\\ & \;=\left(1+\frac{k}{M}\right)\frac{1}{\left(1+1/\sqrt{M}\right)}P[\chi_{1}^{1}=A_{1},\chi_{1}^{2}=A_{1}]. \end{array}$$

Similarly,


$$\begin{array}{rl} & P\left[\chi_{1}^{1}=a_{1},\chi_{1}^{2}=a_{1}|\;\sum_{l=1}^{M}\frac{\Psi_{l}}{\sqrt{M}}=\frac{k}{\sqrt{M}}\right]\\ & \;=\left(1-\frac{k}{M}\right)\frac{1}{\left(1-1/\sqrt{M}\right)}P[\chi_{1}^{1}=a_{1},\chi_{1}^{2}=a_{1}], \end{array}$$

and


$$\begin{array}{rl} & P\left[\chi_{1}^{1}=a_{1},\chi_{1}^{2}=A_{1}|\;\sum_{l=1}^{M}\frac{\Psi_{l}}{\sqrt{M}}=\frac{k}{\sqrt{M}}\right]\\ & \;=\left\{\left(1+\frac{k}{M}\right)\frac{\left(1/2+2/\sqrt{M}\right)}{\left(1+1/\sqrt{M}\right)}+\left(1-\frac{k}{M}\right)\frac{\left(1/2-2/\sqrt{M}\right)}{\left(1-1/\sqrt{M}\right)}\right\}\\ & \;\times P[\chi_{1}^{1}=a_{1},\chi_{1}^{2}=A_{1}]. \end{array}$$

In view of the Central Limit Theorem, we would expect a “typical” value of *k* to be on the order of $\sqrt{M}$; conditioning has only perturbed the probability that $\Psi_{1}=1$ by a factor $k/M+O(1/\sqrt{M})$, which we expect to be of order $1/\sqrt{M}$. In the purely additive case, which corresponds to taking $p_{+}=p_{-}=1/2$, at the extremes of what is possible (k=±M), we recover complete information about the values of $\chi_{1}^{1}$, $\chi_{2}^{1}$; however, with dominance that is no longer true.

Notice that for the difference between the trait value of an individual and the mean over the population to be order one requires order $\sqrt{M}$ of the loci to be “nonrandom,” but observing the trait does not tell us which of the possible *M* loci these are. Similarly, performing the entirely analogous calculation for pairs of loci, and observing that


(M−2(M+k−4)/2)(M(M+k)/2)=14(1+kM)(1+k−1M−1),

we deduce that,


(19) $$\begin{array}{rl} & P\left[\chi_{1}^{1}=A_{1},\chi_{1}^{2}=A_{1};\chi_{2}^{1}=A_{2},\chi_{2}^{2}=A_{2}|\sum_{l=1}^{M}\frac{\Psi_{l}}{\sqrt{M}}=\frac{k}{\sqrt{M}}\right]\\ & \;=\left(1+\frac{k}{M}\right)\left(1+\frac{k-1}{M-1}\right)\frac{1}{(1+1/\sqrt{M}{)}^{2}}\\ & \;\times P[\chi_{1}^{1}=A_{1},\chi_{1}^{2}=A_{1};\chi_{2}^{1}=A_{2},\chi_{2}^{2}=A_{2}]\\ & \;=P\left[\chi_{1}^{1}=A_{1},\chi_{1}^{2}=A_{1}|\;\sum_{l=1}^{M}\frac{\Psi_{l}}{\sqrt{M}}=\frac{k}{\sqrt{M}}\right]\\ & \;\times P\left[\chi_{2}^{1}=A_{2},\chi_{2}^{2}=A_{2}|\;\sum_{l=1}^{M}\frac{\Psi_{l}}{\sqrt{M}}=\frac{k}{\sqrt{M}}\right]\\ & \;+P[\chi_{1}^{1}=A_{1},\chi_{1}^{2}=A_{1};\chi_{2}^{1}=A_{2},\chi_{2}^{2}=A_{2}]\\ & \;\times \left(1+\frac{k}{M}\right)\frac{1}{(1+1/\sqrt{M}{)}^{2}}\left(\frac{k-1}{M-1}-\frac{k}{M}\right). \end{array}$$

For a “typical” trait value the last term in Equation (19) is order 1/M. When we sum over loci, this is enough to give a nontrivial contribution to the trait value coming from the linkage disequilibrium. However, although observing the trait of a typical individual tells us something about linkage disequilibria, it does not tell us enough to identify which of the order $M^{2}$ pairs of loci are in linkage disequilibrium.

Essentially the same argument will apply to the much more general models that we develop below. In particular, for the infinitesimal model to be a good approximation, the observed parental trait values must not contain too much information about the allelic effect at any given locus, which requires that the parental traits must not be too extreme [corresponding to *k* in our toy model being $O(\sqrt{M})$].

In the additive case, it was enough to control the additional information that we gained about any particular locus from knowledge of the trait value in the parents. This is because, in that case, the variance of the shared contribution within a family is zero and independent Mendelian inheritance at each locus ensures that linkage disequilibria do not distort the variance of the residual component that segregates within families. With dominance, we must estimate the (nontrivial) variance of the shared component, and for this we shall see that we need to control the build up of linkage disequilibrium between pairs of loci. It will turn out that since all pairs of loci are in linkage equilibrium in the ancestral population, any *given* pair of loci will be approximately in linkage equilibrium for the order $\sqrt{M}$ generations for which the infinitesimal approximation is valid.

This does not mean that the linkage disequilibria do not affect the trait values, but because of the very many different combinations of alleles in an individual that are consistent with a given trait, observing the trait tells us very little about the allelic state at a particular locus. The allele at that locus can only ever contribute $O(1/\sqrt{M})$ to the overall trait value.

As the population evolves, and we are able to observe more and more traits on the pedigree, we gain more and more information about the allele that an individual carries at a particular locus. In Barton *et al.* (2017), we considered an additive trait in a population of haploid individuals. In that setting, we showed that for a given individual, one does not gain any more information about the state at a given locus from looking at the trait values on the whole of the rest of the pedigree than one does from observing just the parents of that individual. In our model for diploid individuals with dominance, this is no longer the case; observing the trait values of any relatives, no matter how distant, provides some additional information about the allelic state at a locus. The difference arises from the fact that the contribution that a gene makes to the trait value of an individual depends not only on its own allelic state, but also on that of the other copy of the gene at that locus. As a result, we gain information about the allelic state in a focal individual by observing trait values in any other individuals in the pedigree with which it may be identical by descent at that locus. However, the amount of information gleaned about the allelic state of an individual from observing new individuals in the pedigree will decrease in proportion to the probability of identity, and so for distant relatives in the pedigree is very small; provided our pedigree is not too inbred, and trait values are not too extreme, we can still expect the infinitesimal model to be a good approximation for order $\sqrt{M}$ generations.

### Environmental noise

Our derivations will depend on two approaches to proving asymptotic normality. The first, which we apply to the portion $R_{A}^{i}+R_{D}^{i}$ of the trait values, uses a generalized Central Limit theorem (which allows for the summands to have different distributions), which provides control over the rate of convergence as M→∞. (It is this control that tells us for how many generations we can expect the infinitesimal model to be valid.) However, the Central Limit Theorem guarantees only the rate of convergence of the cumulative distribution function of the normalized sum of effects at different loci. Our proofs exploit convergence to the corresponding probability density function, which may not even be defined. To get around this, we can follow the approach of Barton *et al.* (2017) and make the (realistic) assumption that rather than observing the genetic component of a trait directly, the observed trait has an environmental component with a smooth density. This results in the trait distribution having a smooth density which is enough to guarantee the faster rate of convergence. In addition to the benefit in terms of regularity of the trait distribution, an environmental noise with a smooth distribution also reinforces the property that observing the trait value gives us very little information on the allelic state at a given locus: a continuum of combinations of genetic and environmental components may have led to the observed trait, in which each given locus contributes an infinitesimal amount. (To ensure sufficient regularity of the trait density, we could instead make the assumption that the distribution of allelic effects at every locus has a smooth probability density function.) The approach to proving asymptotic normality of the shared component uses an extension of Stein’s method of exchangeable pairs. Once again in the presence of environmental noise (to ensure that the trait distribution has a smooth density) we recover convergence with an error of order $1/\sqrt{M}$.

If the environmental component is taken to be normally distributed, then exactly as in Barton *et al.* (2017), we can adapt our application of Theorem C1 in Appendix C to write down the conditional distribution of the genetic components given *observed* traits; i.e. traits distorted by a small environmental noise, c.f. Remark F2.

### Assumptions and notation

Recall that we assume that in generation zero, the individuals that found the pedigree are unrelated and sampled from an ancestral population in which all loci are assumed to be in linkage equilibrium. The allelic states at locus *l* on the two chromosomes drawn from the ancestral population will be denoted ${\hat{\chi }}_{l}^{1},{\hat{\chi }}_{l}^{2}$. They are independent draws from a distribution on possible allelic states that we denote by ${\hat{\nu }}_{l}(dx)$. Without loss of generality, by replacing $\phi_{l}({\hat{\chi }}_{l}^{1},{\hat{\chi }}_{l}^{2})$ by


$$\phi_{l}({\hat{\chi }}_{l}^{1},{\hat{\chi }}_{l}^{2})-E[\phi_{l}({\hat{\chi }}_{l}^{1},{\hat{\chi }}_{l}^{2})|{\hat{\chi }}_{l}^{1}]-E[\phi_{l}({\hat{\chi }}_{l}^{1},{\hat{\chi }}_{l}^{2})|{\hat{\chi }}_{l}^{2}]+E[\phi_{l}({\hat{\chi }}_{l}^{1},{\hat{\chi }}_{l}^{2})],$$

and observing that the second and third terms on the right-hand side are functions of ${\hat{\chi }}_{l}^{1}$ and ${\hat{\chi }}_{l}^{2}$, respectively, which we may therefore subsume into $\eta_{l}({\hat{\chi }}_{l})$, we may assume that for any value $x^{\prime }$ of the allelic state at locus *l*, the conditional expectation


(20) $$E[\phi_{l}({\hat{\chi }}_{l},x^{\prime })]=\int \phi_{l}(x,x^{\prime }){\hat{\nu }}_{l}(dx)=0=E[\phi_{l}(x^{\prime },{\hat{\chi }}_{l})].$$

As a consequence, partitioning over the possible values of ${\hat{\chi }}_{l}^{2}$, we have that the cross variation term


(21) $$\begin{array}{rl} E[\eta_{l}({\hat{\chi }}_{l}^{1})\phi_{l}({\hat{\chi }}_{l}^{1},{\hat{\chi }}_{l}^{2})] & =\int E[\eta_{l}(x^{\prime })\phi_{l}(x^{\prime },{\hat{\chi }}_{l}^{2})]{\hat{\nu }}_{l}(dx^{\prime })\\ & =\int \eta_{l}(x^{\prime })E[\phi_{l}(x^{\prime },{\hat{\chi }}_{l}^{2})]{\hat{\nu }}_{l}(dx^{\prime })=0. \end{array}$$

With this modification of $\phi_{l}(x,x^{\prime })$,


(22) $$E[\phi_{l}({\hat{\chi }}_{l}^{1},{\hat{\chi }}_{l}^{2})]=0.$$

Moreover, still without loss of generality, by absorbing the mean into ${\bar{z}}_{0}$, we may assume that


(23) $$E[\eta_{l}({\hat{\chi }}_{l})]=\int \eta_{l}(x){\hat{\nu }}_{l}(dx)=0.$$

In this notation, the genetic component of the trait of an individual in the ancestral population (which we denote by $\hat{Z}$ to make it clear that the following property is specific to individuals in generation 0) is


$$\hat{Z}={\bar{z}}_{0}+\frac{1}{\sqrt{M}}\sum_{l=1}^{M}(\eta_{l}({\hat{\chi }}_{l}^{1})+\eta_{l}({\hat{\chi }}_{l}^{2})+\phi_{l}({\hat{\chi }}_{l}^{1},{\hat{\chi }}_{l}^{2})),$$

and by Equations (22) and (23), we have $E[\hat{Z}]={\bar{z}}_{0}$.

We assume that the scaled allelic effects $\eta_{l}$, $\phi_{l}$ are bounded; $\left|\eta_{l}\right|$, $|\phi_{l}|\leq B$, for all *l*. We also assume that all the quantities in the top part of Table 1 exist in the limit as M→∞.

### Inheritance

We now need some notation for Mendelian inheritance. Recall that i[1] and i[2] are the labels of the parents of individual *i* in our pedigree, each of which contributes exactly one gene at each locus in a given offspring. Mendelian inheritance translates into the property that the gene passed on by parent i[1] was the one inherited from its own “first” parent (i[1])[1] with probability 1/2, or from its “second” parent (i[1])[2] with probability 1/2. Even though we do not distinguish between males and females, it is convenient to think of the chromosomes in individual *i* as being labeled 1 and 2, according to whether they are inherited from i[1] or i[2]. In particular, $\chi_{l}^{i[1],1}$ and $\chi_{l}^{i[1],2}$ will denote the allelic states of the two genes at locus *l* in parent i[1], respectively inherited from its own “first” and “second” parent. Again following the conventions of Barton *et al.* (2017), extended to account for the fact that we are now considering diploid individuals, we use independent Bernoulli(1/2) random variables, $X_{l}^{i}$, $Y_{l}^{i}$ to determine the inheritance of genes 1 and 2, respectively, at locus *l* in individual *i*. Thus, $X_{l}^{i}=1$ if the allelic state of gene 1 at locus *l* in individual *i* is inherited from gene 1 in i[1], and $X_{l}^{i}=0$ if it is inherited from gene 2 in i[1]. Likewise, $Y_{l}^{i}=1$ if the allelic state of gene 2 at locus *l* in individual *i* is inherited from gene 1 in i[2], and $Y_{l}^{i}=0$ if it is inherited from gene 2 in i[2].

In this notation, the trait of individual *i* in generation *t* is given by


(24) $$\begin{array}{rl} Z^{i} & ={\bar{z}}_{0}+A^{i}+D^{i}\\ & \;+\frac{1}{\sqrt{M}}\sum_{l=1}^{M}\left\{\left(X_{l}^{i}-\frac{1}{2}\right)\eta_{l}(\chi_{l}^{i[1],1})+\left(\frac{1}{2}-X_{l}^{i}\right)\eta_{l}(\chi_{l}^{i[1],2}\right) \end{array}$$

(25) (25)+(Yi−12)ηl(χli[2],1)+(12−Yi)ηl(χli[2],2)}+1M∑l=1M{(XliYli−14)ϕl(χli[1],1,χli[2],1)(26)+(Xli(1−Yli)−14)ϕl(χli[1],1,χli[2],2)+((1−Xli)Yli−14)ϕl(χli[1],2,χli[2],1)

(27) $$\;+\left((1-X_{l}^{i})(1-Y_{l}^{i})-\frac{1}{4}\right)\phi_{l}(\chi_{l}^{i[1],2},\chi_{l}^{i[2],2})\},$$

where


(28) $$\begin{array}{rl} A^{i} & =\frac{1}{2\sqrt{M}}\sum_{l=1}^{M}(\eta_{l}(\chi_{l}^{i[1],1})+\eta_{l}(\chi_{l}^{i[1],2})\\ & \;+\eta_{l}(\chi_{l}^{i[2],1})+\eta_{l}(\chi_{l}^{i[2],2})) \end{array}$$

and


(29) $$\begin{array}{rl} D^{i} & =\frac{1}{4\sqrt{M}}\sum_{l=1}^{M}\{\phi_{l}(\chi_{l}^{i[1],1},\chi_{l}^{i[2],1})+\phi_{l}(\chi_{l}^{i[1],1},\chi_{l}^{i[2],2})\\ & \;+\phi_{l}(\chi_{l}^{i[1],2},\chi_{l}^{i[2],1})+\phi_{l}(\chi_{l}^{i[1],2},\chi_{l}^{i[2],2})\}. \end{array}$$

The terms $A^{i}$ and $D^{i}$ are shared by all descendants of the parents i[1] and i[2]. In the third section of this paper, we presented the mean and variance of their sum, conditional on the pedigree P(t). The sums (24)+(25) and (26)+(27) comprise what we previously called $R_{A}^{i}$ and $R_{D}^{i}$, respectively; each has mean zero. They capture the randomness of Mendelian inheritance. They are uncorrelated with $A^{i}+D^{i}$. Again, in a previous section we gave expressions for the variances and covariance of $R_{A}^{i}$ and $R_{D}^{i}$ in terms of the ancestral population and identities generated by the pedigree. These calculations allowed us to identify the mean and variance of the parts $A^{i}+D^{i}$ and $R_{A}^{i}+R_{D}^{i}$ in terms of the classical quantities of quantitative genetics in Table 1. Since we are assuming unlinked loci, the asymptotic normality of these quantities when we condition on the pedigree, but not on the trait values within that pedigree, is an elementary application of Theorem D2 in Appendix D, a generalized Central Limit Theorem which allows for nonidentically distributed summands.

In Barton *et al.* (2017), we showed that in the purely additive case, the vector $(R_{A}^{i}{)}_{i=1}^{N_{t}}$, which determines the joint distribution of the trait values within families in generation *t* (recalling that in the additive case $R_{D}^{i}=0$), is asymptotically a multivariate normal, even when we condition not just on the pedigree relatedness of the individuals in generation *t*, but also on knowing the observed trait values of all individuals in the pedigree up to generation t−1, which we denote by $\tilde{Z}(t-1)$ (notice the difference between this notation and the notation ${\tilde{Z}}_{t}$ for the observed trait of an individual living in generation *t*). Our main result extends this to include dominance, at least under the assumption that the ancestral population was in linkage equilibrium.

With dominance, the expression for the distribution of the mean and variance–covariance matrix of the multivariate normal $Z^{1},\ldots ,Z^{N_{t}}$ conditioned on the pedigree up to generation *t* and some collection of the observed trait values of individuals in that pedigree up to generation t−1 is a sum of the quantities of classical quantitative genetics in Table 1, weighted by four-way identities and deviations of trait values from the mean. In principle, they can be read off from Theorem C1 in Appendix C.

We will focus on proving that conditional on knowing just the trait values of the parents of individual *i* and the pedigree, the components $\left(A^{i}+D^{i}\right)$ and $\left(R_{A}^{i}+R_{D}^{i}\right)$ are both asymptotically normal, but we explain why our proof allows us to extend to the case in which we also know trait values of other individuals. The importance (and surprise) is that given the pedigree relationships between the parents and classical coefficients of quantitative genetics for a base population (assumed to be in linkage equilibrium), knowing the traits of the parents distorts the distribution of their offspring in an entirely predictable way. In particular, this is what we mean when we say that the infinitesimal model continues to hold even with dominance.

The extra challenge compared to the additive case is that, in contrast to the part $R_{A}^{i}+R_{D}^{i}$, where Mendelian inheritance ensures independence of the summands corresponding to different loci even after conditioning on trait values, when we condition on trait values the terms in $A^{i}+D^{i}$ will be (weakly) dependent and proving a Central Limit Theorem becomes more involved.

### Main results

Recall that the trait values that we observe, and therefore on which we condition, are the sum of a genetic component and an independent environmental component; that is, the observed trait value is


$${\tilde{Z}}^{i}\;:\;=Z^{i}+E^{i},$$

where, for convenience, the $\left\{E^{i}\right\}$ are independent $N(0,\sigma_{E}^{2})$-valued random variables. We suppose that the environmental noise is shared by individuals in a family (so we can think of it as part of the component $A^{i}+D^{i}$ of the trait value, whose distribution therefore also has a smooth density).

We write $N_{t}$ for the number of individuals in the population in generation *t*, $\left(Z_{t}^{1},\ldots ,Z_{t}^{N_{t}}\right)$ for the corresponding vector of trait values, and P(t) for the pedigree up to and including generation *t*. A simple application of the Central Limit Theorem gives that


$$\left(Z_{t}^{1},\ldots ,Z_{t}^{N_{t}})\;|\;P(t\right)$$

is asymptotically distributed as a multivariate normal random variable as M→∞. More precisely, let $(\beta_{1},\beta_{2},\ldots ,\beta_{N_{t}})\in R^{N_{t}}$, and write $Z_{\beta }=\sum_{i=1}^{N_{t}}\beta_{i}Z_{t}^{i}$, then using Theorem D2,


$$\left|P\left[\frac{Z_{\beta }-E[Z_{\beta }]}{\sqrt{Var(Z_{\beta })}}\leq z\right]-N(z)\right|\leq \frac{C}{\sqrt{M}\sqrt{Var(Z_{\beta })}}\left(1+\frac{\tilde{C}}{Var(Z_{\beta })}\right),$$

for suitable constants $C,\;\tilde{C}$ (which can be made explicit), where N(z) is the cumulative distribution function for a standard normal random variable. The mean and variance of $Z_{\beta }$ can be read off from Equations (9), (10), and (11).

Our main results concern the components of the trait values of offspring when we condition on the observed trait values of their parents. The following result follows in essentially the same way as the additive case of Barton *et al.* (2017).

Theorem 4 The conditioned residuals $(R_{A}^{i}+R_{D}^{i})|P(t),{\tilde{Z}}^{i[1]}$, ${\tilde{Z}}^{i[2]}$ are asymptotically normally distributed, with an error of order $1/\sqrt{M}$. More precisely, for all z∈R,

$$\begin{array}{rl} & \left|P\left[\frac{R_{A}^{i}+R_{D}^{i}}{\sqrt{Var(R_{A}^{i}+R_{D}^{i})}}\leq z|\;P(t),{\tilde{Z}}^{i[1]},{\tilde{Z}}^{i[2]}\right]-N(z)\right|\\ & \;\leq \frac{1}{\sqrt{M}}\frac{C^{\prime }}{\sqrt{Var(R_{A}^{i}+R_{D}^{i})}}\left(1+\frac{\tilde{C^{\prime }}}{Var(R_{A}^{i}+R_{D}^{i})}\right)\\ & \;\times (1+C(i[1],i[2])) \end{array}$$

where

$$\begin{array}{rl} & C(i[1],i[2])\\ & \;=C^{\prime \prime }\frac{\left|{\tilde{Z}}^{i[1]}-E[{\tilde{Z}}^{i[1]}|P(t-1)]\right|}{\sqrt{Var({\tilde{Z}}^{i[1]})}}+C^{\prime \prime }\frac{\left|{\tilde{Z}}^{i[2]}-E[{\tilde{Z}}^{i[2]}|P(t-1)]\right|}{\sqrt{Var({\tilde{Z}}^{i[2]})}}\\ & \;+C^{\prime \prime \prime }\frac{1}{\sqrt{Var({\tilde{Z}}^{i[1]})}\;p(Var({\tilde{Z}}^{i[1]}),|Z^{i[1]}-E[Z^{i[1]}|P(t-1)]|)}\\ & \;\times \left(1+\frac{1}{Var({\tilde{Z}}^{i[1]})}\right)\\ & \;+C^{\prime \prime \prime }\frac{1}{\sqrt{Var({\tilde{Z}}^{i[2]})}\;p(Var({\tilde{Z}}^{i[2]}),|Z^{i[2]}-E[Z^{i[2]}|P(t-1)]|)}\\ & \;\times \left(1+\frac{1}{Var({\tilde{Z}}^{i[2]})}\right), \end{array}$$

and we have used $p(\sigma^{2},x)$ to denote the density at *x* of a mean zero normal random variable with variance $\sigma^{2}$. The constants $C^{\prime }$, $\tilde{C^{\prime }}$, $C^{\prime \prime }$, $C^{\prime \prime \prime }$ depend only on the bound *B* on the scaled allelic effects. The variances in the expressions above are all calculated conditional on P(t−1), but not on observed parental trait values.

Put simply, the normal approximation is good to an error of order $1/\sqrt{M}$; the constant in the error term will be large, meaning that the approximation will be poor, if the within-family variance somewhere in the pedigree is small or if the observed trait values are very different from their expected values. Just as in the additive case, we could prove an entirely analogous result when we condition on any number of observed trait values in the pedigree, except that with dominance this is at the expense of picking up an extra term in the error for each observed trait value on which we condition. The justification required for this is provided by Appendix H.

What is at first sight more surprising is that the shared component of the trait value within a family, i.e. the random variable A+D+E, is also asymptotically normally distributed, even when we condition on observed parental trait values. Note that the randomness of the shared component comes from the fact that the allelic states underlying the parental traits are still random (they are unobserved). In the case of a purely additive trait, it turns out that the shared component can be simply expressed as the average of the two parental traits and therefore conditioning on these traits renders the shared contribution totally deterministic, but such a simplification no longer occurs when we add dominance, due to the nonlinearity of the allelic contributions in D [see Equation (29)]. Our proof of normality uses the fact that we consider the environmental noise to be shared by individuals within the family; in this way we can guarantee that the shared component of the observed trait value also has a smooth density.

We are only going to prove the result for the shared component of a family in generation one that was produced by selfing (i[1]=i[2]). In what follows, for a given function *h* we write ‖h‖ for the supremum norm of *h*, and $N_{\mu ,\sigma^{2}}(h)$ for the integral of *h* with respect to the distribution of an $N(\mu ,\sigma^{2})$ random variable (whenever this quantity makes sense):


$$N_{\mu ,\sigma^{2}}(h)=\frac{1}{\sqrt{2\pi \sigma^{2}}}\int_{-\infty }^{+\infty }h(z)e^{-(z-\mu {)}^{2}/(2\sigma^{2})}\;dz.$$

Theorem 5 Let W=A+D+E denote the shared component of the trait value in a family in generation one. Let *h* be an absolutely continuous function with $\|h^{\prime }\|<\infty$, then

(30) $$|E[h(W)|i[1]=i[2],{\tilde{Z}}^{i[1]}]-N_{\mu_{W},\sigma_{W}^{2}}(h)|\leq \frac{C\|h^{\prime }\|}{\sqrt{M}},$$

where $\mu_{W}$ is given by Equation (F5), and $\sigma_{W}^{2}$ is the sum of the variance of the environmental noise and the expression in Equation (F21).

Remark 6

1. Although we only prove that $A^{i}+D^{i}+E^{i}$ is asymptotically normal in this special case of an individual in generation one that is produced by selfing, the same arguments will apply in general. However, the expressions involved become extremely cumbersome. By considering selfing, we capture all the complications that arise in later generations (when distinct parents may nonetheless be related).
2. We do not record the exact bound on the constant *C*. It takes the same form as the error function C in Theorem 4, except that the constants $C^{\prime }$, $\tilde{C^{\prime }}$, $C^{\prime \prime }$, $C^{\prime \prime \prime }$ depend on the inbreeding depression ι, as well as the bound *B* on the scaled allelic effects. In particular, just as there, the asymptotic normality will break down if the trait value of the parent is too extreme, or if the variance of the trait values among offspring is too small.
3. Since we are assuming that the environmental noise has a smooth density, convergence in the sense of Equation (30) is sufficient to deduce that the cumulative distribution of $A^{i}+D^{i}+E^{i}$ converges.

**Fig. 8.**
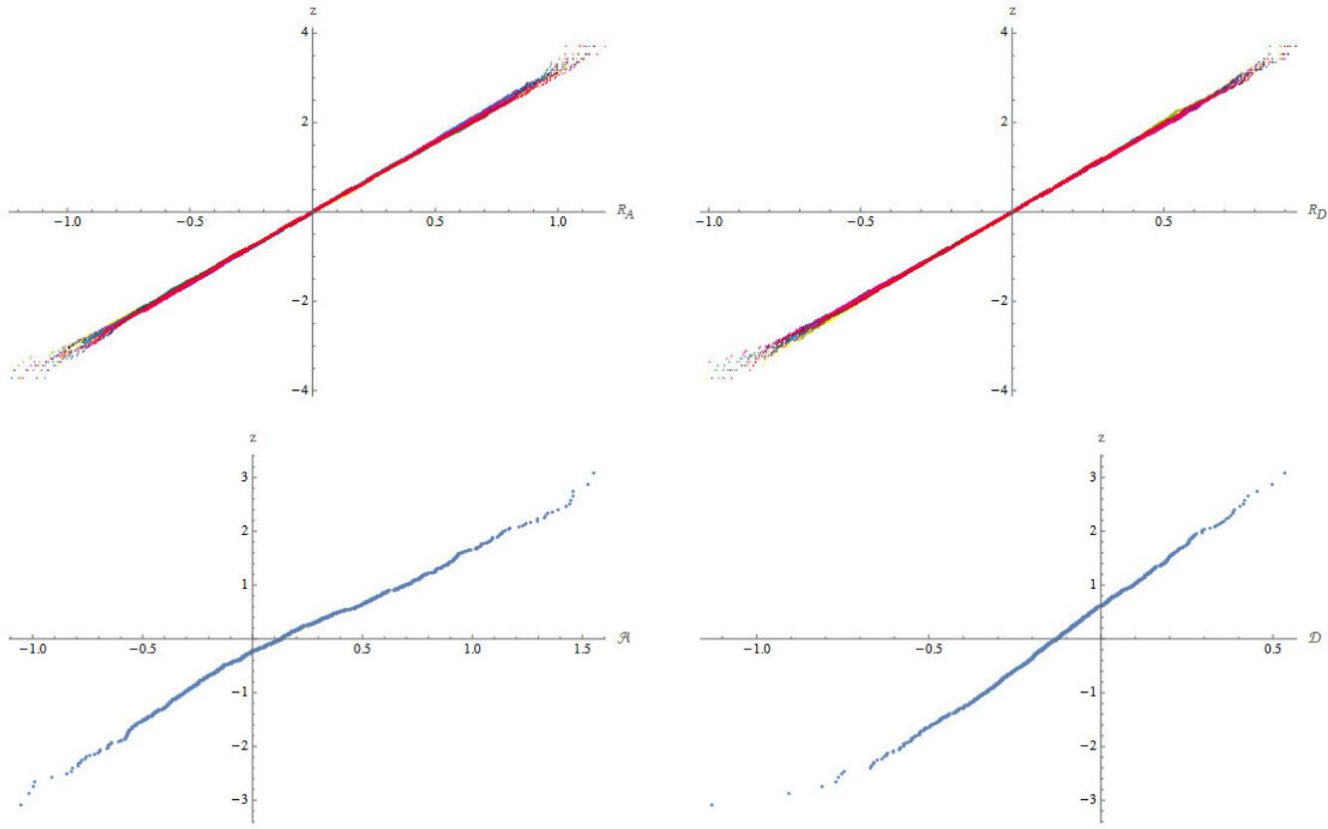
The distributions of the residual (top row: $R_{A}$ , $R_{D}$) and shared (bottom row: A, D) components of phenotype (M=1,000 loci); for each, the cumulative distribution function is plotted as standard deviations of a Gaussian, *z*, so that a normal distribution appears as a straight line. These are calculated from families of 1,000 offspring, from multiple pairs of parents, each replicated 10 times, drawn after 20 generations without selection. The residuals are calculated by subtracting values from the family mean, and pooling across the 10 replicates. Thus, for each family there are 10,000 values; the cumulative distribution function is shown for 10 pairs of parents, in 10 colors. The shared component is calculated by taking the mean of each family, and pooling across 100 pairs of parents and across the 10 replicates. Thus, for each plot there are 1,000 points. There is now some deviation from a Gaussian.

In Fig. 8, we show the cumulative distribution functions of the additive and dominance parts of the shared and residual components of trait within 10 families after 20 generations of neutral evolution, with M=1,000 loci. All 10 within-family distributions of $R_{A}$, $R_{D}$ are close to Gaussian; they vary somewhat in slope, since families vary in identity coefficients (see Fig. 5), but this is not apparent in these plots. The normal approximation is better for the residual components than for the shared component. This may be due to the fact that the random variables encoding Mendelian inheritance at different loci are independent and identically distributed, which makes the summands in the expressions for $R_{A}$ and $R_{D}$ more weakly dependent than the summands in A and D, leading to faster convergence to a Gaussian distribution. This also explains why we need a more elaborate approach to show convergence of the shared parts to Gaussians.

### Strategy of the derivation

Our first task will be to show that conditional on the pedigree, the distribution of the trait values in generation *t* is approximately multivariate normal (with an appropriate error bound). Since Mendelian inheritance ensures that (before we condition on knowing any of the previous trait values in the pedigree) the allelic states at different loci are independent, this is a straightforward application of a generalized Central Limit Theorem (generalized because the summands are not required to all have the same distribution). Just as in Barton *et al.* (2017), we can keep track of the error that we are making in assuming a normal approximation at each generation. In this way we see that, under our assumptions, the infinitesimal model can be expected to be a good approximation for order $\sqrt{M}$ generations.

The same Central Limit Theorem guarantees that the joint distribution of $\left(Z^{i[1]},Z^{i[2]},A^{i}+D^{i}\right)$ is asymptotically normally distributed as the number of loci tends to infinity. This certainly suggests that the conditional distribution of $A^{i}+D^{i}$ given $Z^{i[1]}$, $Z^{i[2]}$ should be (approximately) normal with mean and variance predicted by standard results on conditioning a multivariate normal distribution on some of its marginals (which we recall in Theorem C1). However, this is not immediate. It is possible that the conditioning forces the distribution on to the part of our probability space where the normal approximation breaks down.

To verify that the conditional distribution is asymptotically normal, we shall show that observing the trait value of an individual provides very little information about their allelic state at any particular locus, or any particular pair of loci, and consequently conditioning on parental trait values provides very little information about allelic states in their offspring. This is (essentially) achieved through an application of Bayes’ rule, although some care is needed to control the cumulative error across loci. We use this to calculate the first and second moments of $A^{i}+D^{i}$ conditional on ${\tilde{Z}}^{i[1]}$, ${\tilde{Z}}^{i[2]}$. The fact that they agree with the predictions of Theorem C1 depends crucially on the assumption that dominance is “balanced,” in the sense that the inbreeding depression ι is well defined. This quantity enters not just in the expression for the expected trait value of inbred individuals, but also in our error bounds, c.f. Remark F4.

Of course checking that the first two moments of the conditional distribution of $A^{i}+D^{i}$ are (approximately) consistent with asymptotic normality is not enough to prove that the conditioned random variable is indeed (approximately) normal. Moreover, we cannot apply our generalized Central Limit Theorem to this term. Instead we use a generalization of Stein’s method of “exchangeable pairs” (outlined in Appendix D), which relies on our ability to control the (weak) dependence between the contributions to $A^{i}+D^{i}$ from different loci that is induced by the conditioning. We present the details in the case of identical parents (which is the case in which normality is most surprising) in Appendix G.

We only present our results in the case in which we condition on the parental traits of a single individual in generation *t*. Just as in the additive case, this can be extended to conditioning on any combination of traits in the pedigree up to generation t−1, but the expressions involved become unpleasantly complex. Instead of writing them out, we content ourselves with explaining the only step that requires a new argument. We must show that knowing the traits of all individuals up to generation t−1 does not provide enough information about the allelic states at any particular locus in an individual in generation *t* to destroy the asymptotic normality of its trait value. This is justified in Appendix H using the fact that, because of Mendelian inheritance, the amount of information gleaned about an allele carried by individual *i* from looking at the trait value of one its relatives, is proportional to the probability of identity with that individual as dictated by the pedigree.

### Asymptotic normality conditional on the pedigree

We first illustrate the application of the generalized Central Limit Theorem by showing that in the ancestral population, the distribution of $\left(Z_{0}^{1},\ldots ,Z_{0}^{N_{0}}\right)$ is multivariate normal with mean vector $\left({\bar{z}}_{0},\ldots ,{\bar{z}}_{0}\right)$ and variance–covariance matrix $(\sigma_{A}^{2}+\sigma_{D}^{2})\;\mathrm{Id}$, where Id is the identity matrix and $\sigma_{A}^{2}$ and $\sigma_{D}^{2}$ were defined in Table 1.

To prove this, it is enough to show that for any choice of $\beta =(\beta_{1},\ldots ,\beta_{N_{0}})\in R^{N_{0}}$,


$$\sum_{\;j=1}^{N_{0}}\beta_{j}Z^{j}\to Z_{\beta },$$

where $Z_{\beta }$ is normally distributed with mean ${\bar{z}}_{0}\sum_{\;j=1}^{N_{0}}\beta_{j}$ and variance $(\sigma_{A}^{2}+\sigma_{D}^{2})\sum_{\;j=1}^{N_{0}}\beta_{j}^{2}$. We apply Theorem D2, due to Rinott (1994), which provides control of the rate of convergence as M→∞. It is convenient to write $\left\|\beta {\|}_{1}=\sum_{\;j=1}^{N_{0}}|\beta_{j}\right|$ and $\|\beta {\|}_{2}^{2}=\sum_{\;j=1}^{N_{0}}\beta_{j}^{2}$. Let us write


$$\Psi_{l}=(\eta_{l}({\hat{\chi }}_{l}^{1})+\eta_{l}({\hat{\chi }}_{l}^{2})+\phi_{l}({\hat{\chi }}_{l}^{1},{\hat{\chi }}_{l}^{2})),$$

and we abuse notation by writing $\Psi_{l}^{j}$ for this quantity in the *j*th individual in generation zero. Set $E_{l}=\sum_{\;j=1}^{N_{0}}\beta_{j}\Psi_{l}^{j}$. Recalling our assumption that all $\eta_{l}$ and $\phi_{l}$ are bounded by some constant *B*, so that the sum of the scaled effects at each locus is bounded by 3B, we have that $\left|E_{l}\right|$ is bounded by $3B\|\beta {\|}_{1}$ for all *l*. Moreover, since the individuals that found the pedigree are assumed to be unrelated and sampled from an ancestral population in which all loci are in linkage equilibrium, using Equations (22) and (23), we find that


$$E\left[\sum_{l=1}^{M}E_{l}\right]=0,\;Var\left(\sum_{l=1}^{M}E_{l}\right)=M\|\beta {\|}_{2}^{2}(\sigma_{A}^{2}+\sigma_{D}^{2}).$$

Theorem D2 then yields


$$\begin{array}{rl} & \left|P\left[\frac{\sum_{i=1}^{N_{0}}\beta_{i}(Z^{i}-{\bar{z}}_{0})}{\|\beta {\|}_{2}\sqrt{\sigma_{A}^{2}+\sigma_{D}^{2}}}\leq z\right]-N(z)\right|\\ & \;\leq \frac{1}{\sqrt{M}\|\beta {\|}_{2}\sqrt{\sigma_{A}^{2}+\sigma_{D}^{2}}}\{\sqrt{\frac{1}{2\pi }}3B\|\beta {\|}_{1}\\ & \;+\frac{16}{\|\beta {\|}_{2}\sqrt{\sigma_{A}^{2}+\sigma_{D}^{2}}}(3B{)}^{2}\|\beta {\|}_{1}^{2}\\ & \;+10\left(\frac{1}{\left\|\beta {\|}_{2}^{2}(\sigma_{A}^{2}+\sigma_{D}^{2}\right)}\right)(3B\|\beta {\|}_{1}{)}^{3}\}. \end{array}$$

Here, N is the cumulative distribution function of a standard normal random variable. The right-hand side can be bounded above by


(31) $$\frac{C(\|\beta {\|}_{1})}{\|\beta {\|}_{2}\sqrt{M}\sqrt{\sigma_{A}^{2}+\sigma_{D}^{2}}}\left(1+\frac{1}{\|\beta {\|}_{2}^{2}\left(\sigma_{A}^{2}+\sigma_{D}^{2}\right)}\right),$$

for a suitable constant *C*. In particular, taking $\beta_{k}=0$ for k≠j and $\beta_{j}=1$, we read off that the rate of convergence to the normal distribution of $Z_{0}^{j}$ as the number of loci tends to infinity is order $1/\sqrt{M}$. Note that the normal approximation is poor if the variance $\sigma_{A}^{2}+\sigma_{D}^{2}$ is small.

Exactly the same argument shows that the distribution of $\left(Z^{1},\ldots ,Z^{N_{t}}\right)$ of the individuals in generation *t* converges to that of a multivariate normal, with mean vector $\left({\bar{z}}_{0}+\iota F_{11},\ldots ,{\bar{z}}_{0}+\iota F_{N_{t}N_{t}}\right)$ and variance–covariance matrix determined by Equations (10) and (11).

Our proof of asymptotic normality of $A^{i}+D^{i}$ conditional on the observed trait values of parents will exploit that the joint distribution of $\left(A^{i}+D^{i},Z^{i[1]},Z^{i[2]}\right)$ is asymptotically normal, also with an error of order $1/\sqrt{M}$. This time we show that $\beta_{1}Z^{i[1]}+\beta_{2}Z^{i[2]}+\beta_{3}(A^{i}+D^{i})$ is asymptotically normal for every choice of the vector $(\beta_{1},\beta_{2},\beta_{3})\in R^{3}$. We apply Theorem D2 with


$${\tilde{E}}_{l}=\beta_{1}\Psi_{l}(i[1])+\beta_{2}\Psi_{l}(i[2])+\beta_{3}\Phi_{l}^{i},$$

where


$$\Psi_{l}(i[1])=\eta_{l}(\chi_{l}^{i[1],1})+\eta_{l}(\chi_{l}^{i[1],2})+\phi_{l}(\chi_{l}^{i[1],1},\chi_{l}^{i[1],2}),$$

with a symmetric expression for $\Psi_{l}(i[2])$, and


$$\begin{array}{rl} \Phi_{l}^{i} & =\frac{1}{2}(\eta_{l}(\chi_{l}^{i[1],1})+\eta_{l}(\chi_{l}^{i[1],2})+\eta_{l}(\chi_{l}^{i[2],1})+\eta_{l}(\chi_{l}^{i[2],2}))\\ & \;+\frac{1}{4}(\phi_{l}(\chi_{l}^{i[1],1},\chi_{l}^{i[2],1})+\phi_{l}(\chi_{l}^{i[1],1},\chi_{l}^{i[2],2})\\ & \;+\phi_{l}(\chi_{l}^{i[1],2},\chi_{l}^{i[2],1})+\phi_{l}(\chi_{l}^{i[1],2},\chi_{l}^{i[2],2})). \end{array}$$

Theorem D2 then shows that the difference between the cumulative distribution function of $\beta_{1}Z^{i[1]}+\beta_{2}Z^{i[2]}+\beta_{3}(A^{i}+D^{i})$ and that of a normal random variable with the corresponding mean and variance can be bounded by Equation (31) with $\left\|\beta {\|}_{2}^{2}(\sigma_{A}^{2}+\sigma_{D}^{2}\right)$ replaced by $Var(\beta_{1}Z^{i[1]}+\beta_{2}Z^{i[2]}+\beta_{3}(A^{i}+D^{i}))$, which can be deduced from the expressions for the variance and covariance of $\Psi_{l}^{i[1]}$, $\Psi_{l}^{i[2]}$ and $\Phi_{l}^{i}$ that are calculated in Appendix B and recorded in Equations (10), (11), and (16).

### Conditioning on trait values of the parents

We suppose that for each *i*, we know the parents of the individual *i* and their trait values $Z^{i[1]}$ and $Z^{i[2]}$. We shall treat the shared components $\left(A^{i}+D^{i}\right)$ and the residuals $\left(R_{A}^{i}+R_{D}^{i}\right)$ separately. Both will converge to multivariate normal distributions which are independent of one another.

Mendelian inheritance ensures that the contributions to $R_{A}^{i}+R_{D}^{i}$ from different loci are independent and so normality becomes an easy consequence of Theorem D2 once we have shown that the information gleaned from knowing the trait values only perturbs the distribution by order $1/\sqrt{M}$. This is checked in Equation (F7) and the proof then closely resembles the proof in the additive setting of Barton *et al.* (2017) and so we omit the details.

The proof that $\left(A^{i}+D^{i}\right)$ is normal is more involved as once we condition on the trait values in the parents, the contributions $\Phi_{l}^{i}$ for l=1,…,M will all be (weakly) correlated. Our approach uses an extension of Stein’s method of exchangeable pairs which we recall in Appendix D and apply to our setting in Appendix G. This calculation is more delicate, but the key is that our conditioning induces very weak dependence between loci. The deviation from normality is controlled by


$$\begin{array}{rl} & \frac{1}{P[{\tilde{Z}}^{i[1]}=z_{1},{\tilde{Z}}^{i[2]}=z_{2},A^{i}+D^{i}+E^{i}=w]}\\ & \;\times \frac{\partial }{\partial z_{1}}P[{\tilde{Z}}^{i[1]}=z_{1},{\tilde{Z}}^{i[2]}=z_{2},A^{i}+D^{i}+E^{i}=w], \end{array}$$

and the corresponding quantity for the partial derivative with respect to $z_{2}$ (both to be interpreted as ratios of densities) evaluated at ${\tilde{Z}}^{i[1]}$, ${\tilde{Z}}^{i[2]}$ respectively. (We recall that $\tilde{Z}$ denotes observed trait value.) The normal approximation will break down if the trait values are too extreme or if the pedigree is too inbred.

## Discussion

The essence of the infinitesimal model is that the distribution of a polygenic trait across a pedigree is multivariate normal. Necessarily, if some individuals are selected (that is, if we condition on their trait values), there can be an arbitrary distortion away from Gaussian *across the population*. However, conditional on parental values and on the pedigree, offspring within each family still follow a Gaussian distribution. This was shown in Barton *et al.* (2017) in the purely additive case, and is extended here to the case with dominance; the only difference being that with dominance, the part of the trait shared by all siblings, A+D, is now still random even when conditioning on the parental traits (observing the parental traits does not give us full information on the contribution of the parental alleles to the average offspring trait as it did in the purely additive case), and the most difficult part of our analysis consists in showing that this shared contribution is also Gaussian. Our results strongly rely on our assumption that inbreeding depression, ι, is finite (it is zero in the purely additive case). Armed with these results, the classic theory for neutral evolution of quantitative traits can be used to predict evolution, even under selection. Theorems 4 and 5 show that this infinitesimal limit holds with dominance, at least over timescales of order square root of the number of loci. Indeed, they show that conditional on the parental traits, the distance between the distributions of the components of the offspring trait and a normal distribution is of the order of $1/\sqrt{M}$. Hence, the distance between the trait distribution of an individual and the infinitesimal approximation increases in every generation by a factor of order $1/\sqrt{M}$, and the error bound becomes macroscopic (i.e. order 1) after of the order of $\sqrt{M}$ generations.

Our work provides some mathematical justification for the ubiquity of the Gaussian, and the empirical success of quantitative genetics—a success which is remarkable, given the complex interactions that underlie most traits. The limit is not universal: a nonlinear transformation of a Gaussian trait leads to a non-Gaussian distribution, and failure of the infinitesimal model. This is because epistatic and dominance interactions then have a systematic direction, which violates the terms of the Central Limit Theorem. (Recall that in our toy example in the section on modeling Mendelian inheritance, we needed a “balance” in the dominance component, which we see reflected in our main results in the requirement that ι be well defined.) Nevertheless, if the population is restricted to a range that is narrow relative to the extremes that are genetically possible, then the infinitesimal model may be accurate, even if the genotype-phenotype map is not linear. This links to another way to understand our results: if very many genotypes can generate the same phenotype, then knowing the trait value gives us negligible information about individual allele frequencies. To put this another way, the infinitesimal limit implies that selection on individual alleles is weak relative to random drift ($N_{e}s∼1$), so that neutral evolution at the genetic level is barely perturbed by selection on the trait (Robertson 1960).

If traits truly evolve in this infinitesimal regime, then it will be impossible to find any genomic trace of their response to selection. This extreme view is contradicted by finding an excess of “signatures” of selection in candidate genes, though it might nevertheless be that these signals are generated by alleles with modest $N_{e}s$, such that the infinitesimal model remains accurate for the trait. Indeed, Boyle *et al.* (2017) argue that the very large numbers of single nucleotide polymorphisms that are typically implicated in genome-wide association studies for complex traits implies an “omnigenic” view, in which trait variance is largely due to genes with no obvious functional relation to the trait. Frequencies of nonsynonymous and synonymous mutations suggest that selection on deleterious alleles is typically much stronger than drift ($N_{e}s\gg 1$; Charlesworth 2015). However, it might still be that selection on the focal trait is comparable with drift, even if the total selection on alleles is much stronger. Whether the infinitesimal model accurately describes trait evolution under such a pleiotropic model is an interesting open question.

In principle, we can simulate the infinitesimal model exactly, by generating offspring from the appropriate Gaussian distributions. For the additive case, this is straightforward, since we only need follow the breeding value of each individual, and the matrix of relationships amongst individuals (e.g. Barton and Etheridge 2011, 2018). However, to simulate the infinitesimal model with dominance, we need to track four-way identities, which is only feasible for small populations (<30, say).

We have not set out the extension of the infinitesimal model to structured populations in detail. In principle, this just requires that we track the identities within and between the various classes of individual. One motivation for the present theoretical work was to extend our infinitesimal model of “evolutionary rescue” (Barton and Etheridge 2018) to include inbreeding depression and partial selfing. This should be feasible, provided that we do not need to track identities between specific individuals, but instead, group individuals according to the time since their most recent outcrossed ancestor—an approach applied successfully by Sachdeva (2019). Already, Lande and Porcher (2015) applied the infinitesimal model to a deterministic model of partial selfing, while Roze (2016) analyzed an explicit multilocus model of partial selfing, allowing for dominance and drift, assuming that all loci are equivalent, and that linkage disequilibria are weak.

One of the most obviously unreasonable assumptions of the classical infinitesimal model, and the extension described here, is that there are an infinite number of unlinked loci. Santiago (1998) showed how loose linkage could be approximated by averaging over pairwise linkage disequilibria. In the additive case, the infinitesimal model can be defined precisely for a linear genome, by assuming that very many genes are spread uniformly over the genome (Sachdeva and Barton 2018). The techniques used in our approach are not robust to (even moderately) high levels of linkage, as groups of genes passed on together will decrease the number of “independent” units of heritable contributions to the trait value, leading to an effective number of loci $M_{\mathrm{eff}}$ too low for the Gaussian approximation to be valid (or more precisely, for the bound between the trait distribution and the appropriate Gaussian distribution in Theorems 4 and 5 to be small). In this case, one needs to consider explicit models of recombination that are out of the scope of this work.

The main value of the infinitesimal model may be to show that trait evolution depends on only a few macroscopic parameters; even if we still make explicit multilocus simulations, this focuses attention on those key parameters, and gives confidence in the generality of our results. Quantitative genetics has developed quite separately from population genetics. Although the theoretical synthesis half a century ago (Robertson 1960; Bulmer 1971; Lande 1975) stimulated much subsequent work (empirical as well as theoretical), the failure to find a practicable approximation for the evolution of the genetic variance (e.g. Turelli and Barton 1994) was an obstacle to further progress. The infinitesimal model provides a justification for neglecting the intractable effects of selection on the variance components, and treating them as evolving solely due to drift and migration. This approach may be helpful for understanding evolution in the short and even medium term.

## Data Availability

The code and data produced for this work and used in this article can be found in the public repository (Barton 2023).

## Appendices

The appendices are organized as follows. Appendix A discusses a simple algorithm to compute identity coefficients. In Appendix B, we derive the mean and covariances of the shared and residual parts of the offspring trait knowing the pedigree (but not the parental traits). In Appendix C, we recall a standard result for conditioning multivariate normal random vectors on their marginal values, while in Appendix D we recall the generalized Central Limit Theorems that will be needed to obtain the normal distribution of the offspring trait components conditional on the parental traits. In Appendix E, we prove some key lemmas on conditional allelic distributions that we use in Appendix F to compute the mean and variance of trait values conditional on the pedigree and on parental traits. The convergence of the shared component of the trait to a Gaussian random variable, as the number of loci tends to infinity, is obtained in Appendix G. Finally, in Appendix H we investigate how information accumulates when we condition on knowing more ancestral traits than those of the parents.

## Appendix A: Calculating identity coefficients

### Recursions for pairwise identity by descent

Two-way identities are readily expressed as solutions to a recurrence. The recursion for *F* can be written in terms of a *pedigree matrix*, $P_{i,k}(t)$, which gives the probability that a gene in individual *i* in generation *t* came from parent *k* in generation (t−1); each row has two nonzero entries each with value 1/2 (the entries corresponding to the indices of the two parents, since the gene may have been inherited from either parent with the same probability), unless the individual is produced by selfing, in which case there is a single entry with value 1 (that corresponding to the index of the single parent). Observe that the matrices P(t) are totally determined by knowledge of the pedigree. In contrast to Barton *et al.* (2017), where we focused on haploids, here we necessarily have to deal with diploids. For diploids, the recursion for *F* is

(A1)
$$F_{ij}(t)=\sum_{k,l}P_{i,k}(t)P_{\;j,l}(t)F_{kl}^{*}(t-1),$$

where

$$F_{kl}^{*}=F_{kl}\;\text{if }k\neq l,\;F_{kk}^{*}=\frac{1}{2}(1+F_{kk}).$$

The quantity $F_{kl}^{*}$ is the probability of identity of two genes drawn *independently* from individuals *k* and *l* (this independent drawing corresponds to Mendelian inheritance); if k=l, then we may either pick the same gene twice, which happens with probability 1/2 (and since the two genes are identical, they are also identical by descent), or pick the two genes of individual *k*, again with probability 1/2, and their probability of identity by descent is then $F_{kk}$ by definition. Restating (A1) in words, the probability that a gene taken in individual *i* and a gene taken in individual *j*, both in generation *t*, are identical by descent is equal to the sum over all potential pairs (k,l) of parents in the previous generation (t−1) of the probability that the gene in *i* descends from *k*, the gene in *j* descends from *l* and that the “parental” genes in *k* and *l* are themselves identical by descent.

### Calculating two-, three-, and four-way identities

Several papers have developed algorithms for calculating identity coefficients, given a pedigree (Karigl 1981; Abney 2009; García-Cortés 2015; Kirkpatrick *et al.* 2019). These assume a single genetic locus, and primarily consider the nine condensed identity coefficients of Fig. B1 that describe the relationship between two diploid individuals. This body of work has developed algorithms that can efficiently calculate identity coefficients involving two individuals, across large pedigrees. Karigl (1982) considers (but does not implement) calculation of identities amongst more than two individuals.

Here, we define and implement a (fairly) simple algorithm that deals with multiple sets of genes across multiple individuals. The corresponding code in *Mathematica* can be found in supplementary material (Barton 2023). This is unlikely to be as efficient as existing algorithms for identities amongst one set of genes across two individuals; it is limited by the need to calculate and store identities amongst very many sets of ancestral genes, corresponding to the very many routes by which genes may descend through the pedigree.

First, we establish our notation. The two genes in each individual each receive a separate label. Thus, a gene in individual *i* will have label i={i,1} or i={i,2}. Sets of genes will be generically denoted by $S=\{i_{1},\ldots ,i_{k}\}$. We define $F[S_{1},S_{2},\ldots ,S_{n}]$ to be the probability that the genes contained in each set $S_{1}$, $S_{2}$, … , $S_{n}$ are identical by descent, tracing back to *n* distinct founders in the ancestral population. For example, $F[\{i_{1}\},\{i_{2},i_{3}\},\{i_{4},i_{5}\}]$ is the probability that these three sets of genes, $S_{1}=\{i_{1}\}$, $S_{2}=\{i_{2},i_{3}\}$ and $S_{3}=\{i_{4},i_{5}\}$, each trace back to three distinct founders: one ancestral to $i_{1}$, another one ancestral to $i_{2}$ and $i_{3}$, and a last one ancestral to $i_{4}$ and $i_{5}$. Necessarily, F[{i}]=1 (a single gene traces back to a unique founder), and the probability of identity of genes $i_{1}$ and $i_{2}$ satisfies $F[\{i_{1},i_{2}\}]=1-F[\{i_{1}\},\{i_{2}\}]$. Identities in generation *t* are denoted $F_{t}$.

Given the pedigree, the identities are defined recursively; $F_{t}$ is a linear combination of identities $F_{t-1}$ in the previous generation. Here, we simply outline the algorithm. A detailed explanation in terms of the *Mathematica* code is in the supplementary material (Barton 2023).

In generation t=0 all individuals are assumed unrelated and so $F_{0}[S_{1},\ldots ,S_{n}]$ is set to be 1 if each $S_{k}$ comprises a single gene and these *n* genes are all distinct. Otherwise it is set to zero.

The algorithm proceeds in two steps, first identifying the possible parents from which each gene is descended and then the possible genes within that parent. In this way, a list of all possible scenarios is generated, with each scenario having equal probability. A slight twist here is that if a set contains a single gene in a given individual, that gene traces back to one or other parent of the individual, with equal probability; two genes in the same individual must trace back to the two parents, although those may be the same individual if there is selfing. This list contains many permutations that are equivalent, differing only by order; these are tallied to reduce the number of configurations that need to be stored, resulting in a weighted list. This gives a recursion back to the founder generation. The number of generations and size of pedigree is limited by the amount of memory needed to store the intermediate lists.

## Appendix B: Conditioning on the pedigree

In this section, we illustrate how to recover the expressions for the mean and variance of the two parts $\left(A^{i}+D^{i}\right)$ and $\left(R_{A}^{i}+R_{D}^{i}\right)$ of the trait of individual *i* from identity coefficients of its parents i[1] and i[2] and the classical coefficients of Table 1. Covariances between families are calculated in the same way. We also calculate the covariance between $\left(A^{i}+D^{i}\right)$ and $Z^{i[1]}$ and $Z^{i[2]}$ (given the pedigree) which will be important for establishing the effect of conditioning on the trait values of the parents. Although these expressions are well known, it seems to be hard to find an explicit derivation such as that presented here. Note that at this stage we are only conditioning on the pedigree, not on the observed trait values and the results in this section do not require us to assume the presence of an environmental noise term.

### Notation

Throughout this section, we are going to be calculating quantities conditional on the pedigree. We shall suppress that in our notation.

### Mean and variance of $A^{i}+D^{i}$

The contribution to the trait $Z^{i}$ from the *l*th locus is determined by the four alleles $\chi_{l}^{i[1],1}$, $\chi_{l}^{i[1],2}$,$\chi_{l}^{i[2],1}$, and $\chi_{l}^{i[2],2}$ and the independent Bernoulli random variables $X_{l}^{i}$ and $Y_{l}^{i}$. The mean and variance of $\left(A^{i}+D^{i}\right)$ and $\left(R_{A}^{i}+R_{D}^{i}\right)$ will depend on which combinations of these alleles are identical. First, we introduce some notation for the nine possible identity classes. In Fig. B1, the two copies of each gene in each individual are represented by two (horizontally adjacent) dots. Lines between dots represent identity by descent. It is convenient to think of the genes within an individual as being ordered.

Let us define

(B1)
$$\begin{array}{rl} \Phi (l) & =\frac{1}{2}(\eta_{l}(\chi_{l}^{i[1],1})+\eta_{l}(\chi_{l}^{i[1],2})+\eta_{l}(\chi_{l}^{i[2],1})+\eta_{l}(\chi_{l}^{i[2],2}))\\ & \;+\frac{1}{4}(\phi_{l}(\chi_{l}^{i[1],1},\chi_{l}^{i[2],1})+\phi_{l}(\chi_{l}^{i[1],1},\chi_{l}^{i[2],2})\\ & \;+\phi_{l}(\chi_{l}^{i[1],2},\chi_{l}^{i[2],1})+\phi_{l}(\chi_{l}^{i[1],2},\chi_{l}^{i[2],2})),\\ \Psi_{l}(i[1]) & =\eta_{l}(\chi_{l}^{i[1],1})+\eta_{l}(\chi_{l}^{i[1],2})+\phi_{l}(\chi_{l}^{i[1],1},\chi_{l}^{i[1],2}), \end{array}$$

and

$$\Psi_{l}(i[2])=\eta_{l}(\chi_{l}^{i[2],1})+\eta_{l}(\chi_{l}^{i[2],2})+\phi_{l}(\chi_{l}^{i[2],1},\chi_{l}^{i[2],2}).$$

For each of the nine possible identity classes between i[1] and i[2], we calculate two quantities from which the mean and variance of $\left(A^{i}+D^{i}\right)$ will readily follow.

$$\begin{array}{ccc} \text{id. state} & E[\frac{1}{M}\sum_{l=1}^{M}\Phi {\left(l\right)}^{2}|\Delta_{\cdot }] & E[\frac{1}{\sqrt{M}}\sum_{l=1}^{M}\Phi (l)|\Delta_{\cdot }]\\ -\;\;\;-\;\;\;-\;\;\;-\;\;\;-\;\;\;-\;\;\;-\;\;\;-\;\;\;-\;\;\;-\;\;\;-\;\;\;-\;\;\;-\;\;\;\;\;\;\;\;\;\;\;\;\;\;\;\;\;\;- & -\;\;\;-\;\;\;-\;\;\;-\;\;\;-\;\;\;-\;\;\;-\;\;\;-\;\;\;-\;\;\;-\;\;\;-\;\;\;-\;\;\;-\;\;\;-\;\;\;-\;\;\;-\;\;\;-\;\;\;-\;\;\;-\;\;\;-\;\;\;-\;\;\;-\;\;\;\;\;\;\;\;\;\;\; & -\;\;\;-\;\;\;-\;\;\;-\;\;\;-\;\;\;-\;\;\;-\;\;\;-\;\;\;-\;\;\;-\;\;\;-\;\;\;-\;\;\;-\;\;\;-\;\;\;-\;\;\;-\;\;\;-\;\;\\ \Delta_{1} & 2\sigma_{A}^{2}+2\sigma_{ADI}+\sigma_{DI}^{2}+\iota^{*} & \iota \\ \Delta_{2} & \sigma_{A}^{2}+\sigma_{D}^{2} & 0\\ \Delta_{3} & \frac{5}{4}\sigma_{A}^{2}+\frac{3}{4}\sigma_{ADI}+\frac{\sigma_{DI}^{2}+\iota^{*}}{4}+\frac{\sigma_{D}^{2}}{4} & \frac{\iota }{2}\\ \Delta_{4} & \frac{3}{4}\sigma_{A}^{2}+\frac{1}{2}\sigma_{D}^{2} & 0\\ \Delta_{5} & \frac{5}{4}\sigma_{A}^{2}+\frac{3}{4}\sigma_{ADI}+\frac{\sigma_{DI}^{2}+\iota^{*}}{4}+\frac{\sigma_{D}^{2}}{4} & \frac{\iota }{2}\\ \Delta_{6} & \frac{3}{4}\sigma_{A}^{2}+\frac{1}{2}\sigma_{D}^{2} & 0\\ \Delta_{7} & \sigma_{A}^{2}+\frac{\sigma_{DI}^{2}+\iota^{*}}{8}+\frac{\sigma_{ADI}}{2}+\frac{\sigma_{D}^{2}}{4} & \frac{1}{4}\iota^{*}\\ \Delta_{8} & \frac{3}{4}\sigma_{A}^{2}+\frac{\sigma_{ADI}}{4}+\frac{\sigma_{DI}^{2}+\iota^{*}}{16}+\frac{3}{16}\sigma_{D}^{2} & \frac{1}{4}\iota \\ \Delta_{9} & \frac{\sigma_{A}^{2}}{2}+\frac{\sigma_{D}^{2}}{4} & 0 \end{array}$$

To see where these expressions come from, consider for example identity state $\Delta_{3}$, with, say, $\chi_{l}^{1}\;:\;=\chi_{l}^{i[1],1}=\chi_{l}^{i[1],2}=\chi_{l}^{i[2],1}\neq \chi_{l}^{i[2],2}=\;:\;\chi_{l}^{2}$, where “=” here means identical by descent. Then, using Equations (21)–(23),

$$\begin{array}{rl} & E\left[\frac{1}{M}\sum_{l=1}^{M}\Phi {\left(l\right)}^{2}\;|\Delta_{3}\right]\\ & \;=\frac{1}{M}\sum_{l=1}^{M}E\left[{\left(\frac{3\eta (\chi_{l}^{1})+\eta (\chi_{l}^{2})}{2}+\frac{2\phi (\chi_{l}^{1},\chi_{l}^{1})+2\phi (\chi_{l}^{1},\chi_{l}^{2})}{4}\right)}^{2}\right]\\ & \;=\frac{5}{4}\sigma_{A}^{2}+\frac{3}{4}\sigma_{ADI}+\frac{1}{4}(\sigma_{DI}^{2}+\iota^{*})+\frac{1}{4}\sigma_{D}^{2}. \end{array}$$

The following quantities can be calculated in the same way. They are important for calculating the covariance between the trait values of parent and offspring (in particular the covariance between $\left(A^{i}+D^{i}\right)$ and $Z^{i[1]}$ and $Z^{i[2]}$) which will dictate the change in distribution of the trait values within families arising from conditioning on knowing the traits of the parents. We record them here for later reference.

$$\begin{array}{ccc} \text{id. state} & E[\frac{1}{M}\sum_{l=1}^{M}\Phi (l)\Psi_{l}(i[1])|\Delta_{\cdot }] & E[\frac{1}{M}\sum_{l=1}^{M}\Phi (l)\Psi_{l}(i[2])|\Delta_{\cdot }]\\ -\;\;\;-\;\;\;-\;\;\;-\;\;\;-\;\;\;-\;\;\;-\;\;\;-\;\;\;-\;\;\;-\;\;\;-\;\;\;-\;\;\;\;\;\;\;\;\;\;\; & -\;\;\;-\;\;\;-\;\;\;-\;\;\;-\;\;\;-\;\;\;-\;\;\;-\;\;\;-\;\;\;-\;\;\;-\;\;\;-\;\;\;-\;\;\;-\;\;\;-\;\;\;-\;\;\;-\;\;\;-\;\;\;-\;\;\;-\;\;\;-\;\;\;-\;\;\;\;\;\;\;\;\;\;\; & -\;\;\;-\;\;\;-\;\;\;-\;\;\;-\;\;\;-\;\;\;-\;\;\;-\;\;\;-\;\;\;-\;\;\;-\;\;\;-\;\;\;-\;\;\;-\;\;\;-\;\;\;-\;\;\;-\;\;\;-\;\;\;-\;\;\;-\;\;\;-\;\;\\ \Delta_{1} & 2\sigma_{A}^{2}+2\sigma_{ADI}+\sigma_{DI}^{2}+\iota^{*} & 2\sigma_{A}^{2}+2\sigma_{ADI}+\sigma_{DI}^{2}+\iota^{*}\\ \Delta_{2} & \sigma_{A}^{2}+\frac{\sigma_{ADI}}{2} & \sigma_{A}^{2}+\frac{\sigma_{ADI}}{2}\\ \Delta_{3} & \frac{3}{2}\sigma_{A}^{2}+\frac{\sigma_{DI}^{2}+\iota^{*}}{2}+\frac{5}{4}\sigma_{ADI} & \sigma_{A}^{2}+\frac{\sigma_{ADI}}{4}+\frac{\sigma_{D}^{2}}{2}\\ \Delta_{4} & \sigma_{A}^{2}+\frac{1}{2}\sigma_{ADI} & \frac{\sigma_{A}^{2}}{2}\\ \Delta_{5} & \sigma_{A}^{2}+\frac{\sigma_{ADI}}{4}+\frac{\sigma_{D}^{2}}{2} & \frac{3}{2}\sigma_{A}^{2}+\frac{\sigma_{DI}^{2}+\iota^{*}}{2}+\frac{5}{4}\sigma_{ADI}\\ \Delta_{6} & \frac{\sigma_{A}^{2}}{2} & \sigma_{A}^{2}+\frac{1}{2}\sigma_{ADI}\\ \Delta_{7} & \sigma_{A}^{2}+\frac{\sigma_{ADI}}{4}+\frac{\sigma_{D}^{2}}{2} & \sigma_{A}^{2}+\frac{\sigma_{ADI}}{4}+\frac{\sigma_{D}^{2}}{2}\\ \Delta_{8} & \frac{3}{4}\sigma_{A}^{2}+\frac{1}{8}\sigma_{ADI}+\frac{1}{4}\sigma_{D}^{2} & \frac{3}{4}\sigma_{A}^{2}+\frac{1}{8}\sigma_{ADI}+\frac{1}{4}\sigma_{D}^{2}\\ \Delta_{9} & \frac{1}{2}\sigma_{A}^{2} & \frac{1}{2}\sigma_{A}^{2} \end{array}$$

We can express two- and three-way identities between the parents in terms of the four-way identities $\Delta_{1},\ldots ,\Delta_{9}$. Recall that we write, for example, $F_{11}$ for the probability of identity of the two genes in i[1] and $F_{12}$ for the probability of identity of two genes, one selected at random from i[1] and one from i[2]. In terms of the nine identity states, we have

$$\begin{array}{rl} F_{11} & =P[\Delta_{1}]+P[\Delta_{2}]+P[\Delta_{3}]+P[\Delta_{4}]\\ F_{22} & =P[\Delta_{1}]+P[\Delta_{2}]+P[\Delta_{5}]+P[\Delta_{6}]\\ F_{12} & =P[\Delta_{1}]+\frac{1}{2}(P[\Delta_{3}]+P[\Delta_{5}]+P[\Delta_{7}])+\frac{1}{4}P[\Delta_{8}]\\ F_{112} & =P[\Delta_{1}]+\frac{1}{2}P[\Delta_{3}]\\ F_{122} & =P[\Delta_{1}]+\frac{1}{2}P[\Delta_{5}]\\ F_{1122} & =P[\Delta_{1}]\\ {\tilde{F}}_{1122} & =P[\Delta_{2}]\\ {\tilde{F}}_{1212} & =P[\Delta_{7}]. \end{array}$$

Combining the above, we find

$$\begin{array}{rl} & E\left[\frac{1}{M}\sum_{l=1}^{M}\Phi (l)\Psi_{l}(i[1])\right]\\ & \;=\frac{\sigma_{A}^{2}}{2}(1+F_{11}+2F_{12})+\frac{\sigma_{ADI}}{2}(F_{11}+F_{12}+2F_{122})\\ & \;+\sigma_{D}^{2}(F_{12}-F_{112})+(\sigma_{DI}^{2}+\iota^{*})F_{112}, \end{array}$$

with a symmetric expression for $\frac{1}{M}\sum_{l=1}^{M}E[\Phi (l)\Psi_{l}(i[2])]$. Similarly,

$$E[(A^{i}+D^{i})]=\frac{1}{\sqrt{M}}\sum_{l=1}^{M}E[\Phi (l)]=\iota F_{12}$$

and

$$\begin{array}{rl} & \frac{1}{M}\sum_{l=1}^{M}E[\Phi {\left(l\right)}^{2}]\\ & \;=\frac{\sigma_{A}^{2}}{2}\left(1+\frac{F_{11}+F_{22}}{2}+2F_{12}\right)+\sigma_{ADI}\left(F_{12}+\frac{F_{112}+F_{122}}{2}\right)\\ & \;+\frac{\sigma_{DI}^{2}+\iota^{*}}{4}\left(F_{12}+F_{112}+F_{122}+F_{1122}\right)\\ & \;+\frac{\sigma_{D}^{2}}{4}\left(1-F_{12}+F_{11}-F_{112}+F_{22}-F_{122}+{\tilde{F}}_{1122}+\frac{1}{2}{\tilde{F}}_{1212}\right)\\ & \;+\frac{1}{4}\iota^{*}{\tilde{F}}_{1212}, \end{array}$$

from which, since for l≠m we are assuming E[Φ(l)Φ(m)]=E[Φ(l)]E[Φ(m)],

$$\frac{1}{M}E[\underset{l\neq m}{\sum_{l=1}^{M}\sum_{m=1}^{M}}\;\Phi (l)\Phi (m)]=(\iota F_{12}{)}^{2}-\iota^{*}F_{12}^{2},$$

and the expression (6) for the variance of $\left(A^{i}+D^{i}\right)$ follows.

Remark B1
Walsh and Lynch (2018) give an expression for the variance when there is linkage disequilibrium. In their notation, $\tilde{f}$ is the probability of identity at two distinct loci. Then for l≠m,

$$E[\Phi (l)\Phi (m)]=\tilde{\;f}E[\Phi_{l}({\hat{\chi }}_{l},{\hat{\chi }}_{l})]E[\Phi_{m}({\hat{\chi }}_{m},{\hat{\chi }}_{m})],$$

so that our expression for

$$\frac{1}{M}E\left[\underset{l\neq m}{\sum_{l=1}^{M}\sum_{m=1}^{M}}\;\Phi (l)\Phi (m)\right]$$

will be multiplied by $\left(\;\tilde{f}/F_{12}^{2}\right)$, resulting (when we subtract $E[A^{i}+D^{i}{]}^{2}$) in an overall expression of $(\;\tilde{f}-F_{12}^{2})\iota^{2}-\tilde{f}\iota^{*}$ in place of $-\iota^{*}F_{12}^{2}$. Correcting for this by adding $\left(\;\tilde{f}-F_{12}^{2})(\iota^{2}-\iota^{*}\right)$ to our expression (11) for the variance of $Z^{i}$ (for which we recall that $F_{12}$ becomes $F_{ii}$), we recover the expression of Walsh and Lynch (2018).

### The covariance between $A^{i}+D^{i}$ and $A^{j}+D^{j}$

To understand the expression (7) for the covariance between $A^{i}+D^{i}$ and $A^{j}+D^{j}$ for i≠j, consider

$$\begin{array}{rl} & E[\{\frac{\eta_{l}(\chi_{l}^{i[1],1})+\eta_{l}(\chi_{l}^{i[1],2})+\eta_{l}(\chi_{l}^{i[2],1})+\eta_{l}(\chi_{l}^{i[2],2})}{2}\\ & \;+\frac{\phi_{l}(\chi_{l}^{i[1],1},\chi_{l}^{i[2],1})+\phi_{l}(\chi_{l}^{i[1],1},\chi_{l}^{i[2],2})}{4}\\ & \;+\frac{\phi_{l}(\chi_{l}^{i[1],2},\chi_{l}^{i[2],1})+\phi_{l}(\chi_{l}^{i[1],2},\chi_{l}^{i[2],2})}{4}\}\\ & \;\times \{\frac{\eta_{l}(\chi_{l}^{\;j[1],1})+\eta_{l}(\chi_{l}^{\;j[1],2})+\eta_{l}(\chi_{l}^{\;j[2],1})+\eta_{l}(\chi_{l}^{\;j[2],2})}{2}\\ & \;+\frac{\phi_{l}(\chi_{l}^{\;j[1],1},\chi_{l}^{\;j[2],1})+\phi_{l}(\chi_{l}^{\;j[1],1},\chi_{l}^{\;j[2],2})}{4}\\ & \;+\frac{\phi_{l}(\chi_{l}^{\;j[1],2},\chi_{l}^{\;j[2],1})+\phi_{l}(\chi_{l}^{\;j[1],2},\chi_{l}^{\;j[2],2})}{4}\}]. \end{array}$$

The 16 terms corresponding to products of additive effects correspond to the 16 different possibilities for the allelic types at locus *l* if we choose one allele at random from individual *i* and one from individual *j*, and the contribution to the expectation will be nonzero precisely if the chosen alleles are identical, in which case they contribute $E[\eta_{l}({\hat{\chi }}_{l}{)}^{2}]$. Summing over *l*, the overall contribution of such terms to the covariance will therefore be $2\sigma_{A}^{2}F_{ij}$.

Similarly, terms involving one factor of $\eta_{l}$ and one $\phi_{l}$ will only be nonzero if all evaluated on the same allelic type, hence the terms multiplied by $F_{iij}$ and $F_{ijj}$ in Equation (7).

Continuing in this way and using that $E[A^{i}+D^{i}]=\iota F_{ii}$, we recover Equation (7).

### The residuals $R_{A}^{i}+R_{D}^{i}$

The corresponding calculations for the mean and variance of the residuals, $R_{A}^{i}+R_{D}^{i}$ follow exactly the same pattern. It is convenient to consider $R_{A}^{i}$ and $R_{D}^{i}$ separately, and then calculate the covariance. The first of these, corresponding to the additive part is very straightforward since it is only going to depend on pairwise identities.

Recall first that

$$\begin{array}{rl} R_{A}^{i} & =\frac{1}{\sqrt{M}}\sum_{l=1}^{M}\left\{\left(X_{i}-\frac{1}{2}\right)\eta_{l}(\chi_{l}^{i[1],1})+\left(\frac{1}{2}-X_{l}^{i}\right)\eta_{l}(\chi_{l}^{i[1],2}\right)\\ & \;+\left(Y_{i}-\frac{1}{2}\right)\eta_{l}(\chi_{l}^{i[2],1})+\left(\frac{1}{2}-Y_{i}\right)\eta_{l}(\chi_{l}^{i[2],2})\}. \end{array}$$

Since the Mendelian inheritance is independent of the allelic states, $R_{A}^{i}$ has mean zero; to establish the variance, we must calculate its square. Since inheritance is independent at distinct loci, only the diagonal terms contribute and we find

$$\begin{array}{rl} & E[(R_{A}^{i}{)}^{2}]\\ & \;=\frac{1}{M}\sum_{l=1}^{M}E[\left\{\left(X_{i}-\frac{1}{2}\right)\eta_{l}(\chi_{l}^{i[1],1})+\left(\frac{1}{2}-X_{l}^{i}\right)\eta_{l}(\chi_{l}^{i[1],2}\right)\\ & \;{+\left(Y_{i}-\frac{1}{2}\right)\eta_{l}(\chi_{l}^{i[2],1})+\left(\frac{1}{2}-Y_{i}\right)\eta_{l}(\chi_{l}^{i[2],2})\}}^{2}]\\ & \;=\frac{1}{4M}\sum_{l=1}^{M}E[(\eta_{l}(\chi_{l}^{i[1],1}){)}^{2}+(\eta_{l}(\chi_{l}^{i[1],2}){)}^{2}\\ & \;+(\eta_{l}(\chi_{l}^{i[2],1}){)}^{2}+(\eta_{l}(\chi_{l}^{i[2],2}){)}^{2}]\\ & \;-\frac{1}{2M}\sum_{l=1}^{M}E[\eta_{l}(\chi_{l}^{i[1],1})\eta_{l}(\chi_{l}^{i[1],2})+\eta_{l}(\chi_{l}^{i[2],1})\eta_{l}(\chi_{l}^{i[2],2})]\\ & \;=\frac{1}{M}\sum_{l=1}^{M}Var(\eta_{l}({\hat{\chi }}_{l}))-\frac{1}{2M}\sum_{l=1}^{M}(F_{11}+F_{22})\;Var(\eta ({\hat{\chi }}_{l}))\\ & \;=\left(1-\frac{F_{11}+F_{22}}{2}\right)\frac{\sigma_{A}^{2}}{2}. \end{array}$$

This is, of course, exactly the expression we would obtain in the purely additive case.

The second residual, $R_{D}^{i}$, also has mean zero, but its variance will now involve higher order identities. Recall that

$$\begin{array}{rl} R_{D}^{i} & =\frac{1}{\sqrt{M}}\sum_{l=1}^{M}\left\{\left(X_{l}^{i}Y_{l}^{i}-\frac{1}{4}\right)\phi_{l}(\chi_{l}^{i[1],1},\chi_{l}^{i[2],1}\right)\\ & \;+\left(X_{l}^{i}(1-Y_{l}^{i})-\frac{1}{4}\right)\phi_{l}(\chi_{l}^{i[1],1},\chi_{l}^{i[2],2})\\ & \;+\left((1-X_{l}^{i})Y_{l}^{i}-\frac{1}{4}\right)\phi_{l}(\chi_{l}^{i[1],2},\chi_{l}^{i[2],1})\\ & \;+\left((1-X_{l}^{i})(1-Y_{l}^{i})-\frac{1}{4}\right)\phi_{l}(\chi_{l}^{i[1],2},\chi_{l}^{i[2],2})\}. \end{array}$$

Once again, since Mendelian inheritance is independent at different loci, $E[(R_{D}^{i}{)}^{2}]$ will be entirely determined by the diagonal terms. Note that for independent Bernoulli (parameter 1/2) random variables *X* and *Y*,

$$E\left[{\left(XY-\frac{1}{4}\right)}^{2}\right]=E\left[\frac{1}{2}XY+\frac{1}{16}\right]=\frac{3}{16},$$

and

$$E\left[\left(XY-\frac{1}{4}\right)\left(X(1-Y)-\frac{1}{4}\right)\right]=-\frac{1}{16}.$$

So, taking expectations over the variables $X_{l}^{i}$ and $Y_{l}^{i}$, we find

(B2)
$$\begin{array}{rl} & E[(R_{D}^{i}{)}^{2}]\\ & \;=\frac{3}{16M}\sum_{l=1}^{M}E[\phi_{l}(\chi_{l}^{i[1],1},\chi_{l}^{i[2],1}{)}^{2}+\phi_{l}(\chi_{l}^{i[1],1},\chi_{l}^{i[2],2}{)}^{2}\\ & \;+\phi_{l}(\chi_{l}^{i[1],2},\chi_{l}^{i[2],1}{)}^{2}+\phi_{l}(\chi_{l}^{i[1],2},\chi_{l}^{i[2],2}{)}^{2}]\\ & \;-\frac{2}{16M}\sum_{l=1}^{M}E[\phi_{l}(\chi_{l}^{i[1],1},\chi_{l}^{i[2],1})\phi_{l}(\chi_{l}^{i[1],1},\chi_{l}^{i[2],2})\\ & \;+\phi_{l}(\chi_{l}^{i[1],1},\chi_{l}^{i[2],1})\phi_{l}(\chi_{l}^{i[1],2},\chi_{l}^{i[2],1})\\ & \;+\phi_{l}(\chi_{l}^{i[1],1},\chi_{l}^{i[2],1})\phi_{l}(\chi_{l}^{i[1],2},\chi_{l}^{i[2],2})\\ & \;+\phi_{l}(\chi_{l}^{i[1],1},\chi_{l}^{i[2],2})\phi_{l}(\chi_{l}^{i[1],2},\chi_{l}^{i[2],1})\\ & \;+\phi_{l}(\chi_{l}^{i[1],1},\chi_{l}^{i[2],2})\phi_{l}(\chi_{l}^{i[1],2},\chi_{l}^{i[2],2})\\ & \;+\phi_{l}(\chi_{l}^{i[1],2},\chi_{l}^{i[2],1})\phi_{l}(\chi_{l}^{i[1],2},\chi_{l}^{i[2],2})]. \end{array}$$

The first term depends only on pairwise identities and we see immediately that it is

$$\begin{array}{rl} & \frac{3}{4M}F_{12}\sum_{l=1}^{M}E[\phi_{l}({\hat{\chi }}_{l},{\hat{\chi }}_{l}{)}^{2}]+\frac{3}{4M}(1-F_{12})\sum_{l=1}^{M}E[\phi_{l}({\hat{\chi }}_{l}^{1},{\hat{\chi }}_{l}^{2}{)}^{2}]\\ & \;=\frac{3}{4}F_{12}(\sigma_{DI}^{2}+\iota^{*})+\frac{3}{4}(1-F_{12})\sigma_{D}^{2}. \end{array}$$

The second term in Equation (B2) is most easily calculated conditional on identity class. Let us write Ξ(l) for the summand corresponding to locus *l*.

$$\begin{array}{cc} \text{identity state} & E[\frac{1}{M}\sum_{l=1}^{M}\Xi (l)|\Delta_{\cdot }]\\ -\;\;\;-\;\;\;-\;\;\;-\;\;\;-\;\;\;-\;\;\;-\;\;\;-\;\;\;-\;\;\;-\;\;\;-\;\;\;-\;\;\;-\;\;\;-\;\;\;-\;\;\;-\;\;\;-\;\;\;-\;\;\;\;\;\;\;\;\;\;\;\;\;\; & -\;\;\;-\;\;\;-\;\;\;-\;\;\;-\;\;\;-\;\;\;-\;\;\;-\;\;\;-\;\;\;-\;\;\;-\;\;\;-\;\;\;-\;\;\;-\;\;\;-\;\;\;-\;\;\\ \Delta_{1} & \frac{3}{4}(\sigma_{DI}^{2}+\iota^{*})\\ \Delta_{2} & \frac{3}{4}\sigma_{D}^{2}\\ \Delta_{3} & \frac{1}{8}(\sigma_{D}^{2}+\sigma_{DI}^{2}+\iota^{*})\\ \Delta_{4} & \frac{1}{4}\sigma_{D}^{2}\\ \Delta_{5} & \frac{1}{8}(\sigma_{D}^{2}+\sigma_{DI}^{2}+\iota^{*})\\ \Delta_{6} & \frac{1}{4}\sigma_{D}^{2}\\ \Delta_{7} & \frac{1}{8}(\sigma_{D}^{2}+\iota^{*})\\ \Delta_{8} & 0\\ \Delta_{9} & 0 \end{array}$$

Using our notation for identities, this becomes

$$\begin{array}{rl} & -\frac{1}{4}(F_{1122}+F_{122}+F_{112})(\sigma_{DI}^{2}+\iota^{*})-\frac{1}{4}\iota^{*}{\tilde{F}}_{1212}\\ & -\frac{1}{4}(F_{11}-F_{112}+F_{22}-F_{122}+{\tilde{F}}_{1122}+\frac{1}{2}{\tilde{F}}_{1212})\sigma_{D}^{2}. \end{array}$$

Thus,

$$\begin{array}{rl} & E[(R_{D}^{i}{)}^{2}]\\ & \;=\frac{1}{4}(3F_{12}-F_{1122}-F_{122}-F_{112})(\sigma_{DI}^{2}+\iota^{*})-\frac{1}{4}\iota^{*}{\tilde{F}}_{1212}\\ & \;+\frac{1}{4}(3(1-F_{12})-(F_{22}-F_{122})-(F_{11}-F_{112})\\ & \;-{\tilde{F}}_{1122}-\frac{1}{2}{\tilde{F}}_{1212})\sigma_{D}^{2}. \end{array}$$

### The covariance of $R_{A}^{i}$ and $R_{D}^{i}$

Since $R_{A}^{i}$ has mean zero, it suffices to calculate $E[R_{A}^{i}R_{D}^{i}]$. We need to establish the mean of

(B3)
$$\begin{array}{rl} & \left\{\left(X-\frac{1}{2}\right)\eta_{l}(\chi_{l}^{i[1],1})+\left(\frac{1}{2}-X\right)\eta_{l}(\chi_{l}^{i[1],2}\right)\\ & \;+\left(Y-\frac{1}{2}\right)\eta_{l}(\chi^{i[2],1})+\left(\frac{1}{2}-Y\right)\eta_{l}(\chi_{l}^{i[2],2})\}\\ & \;\times \left\{XY\phi_{l}(\chi_{l}^{i[1],1},\chi_{l}^{i[2],1})+X(1-Y)\phi_{l}(\chi_{l}^{i[1],1},\chi_{l}^{i[2],2}\right)\\ & \;+(1-X)Y\phi_{l}(\chi_{l}^{i[1],2},\chi_{l}^{i[2],1})\\ & \;+(1-X)(1-Y)\phi_{l}(\chi_{l}^{i[1],2},\chi_{l}^{i[2],2})\}. \end{array}$$

We have been able to drop the “−1/4” terms in the second bracket since $E[R_{A}^{i}]=0$.

Now

$$\begin{array}{rl} E\left[\left(X-\frac{1}{2}\right)XY\right] & =\frac{1}{2}E[XY]=\frac{1}{8},\\ E\left[\left(X-\frac{1}{2}\right)(1-X)Y\right] & =-\frac{1}{2}E[(1-X)Y]=-\frac{1}{8}, \end{array}$$

and so the mean of Equation (B3) is that of

$$\begin{array}{rl} & \frac{1}{8}\{(\eta_{l}(\chi_{l}^{i[1],1})-\eta_{l}(\chi^{i[1],2}))\phi_{l}(\chi_{l}^{i[1],1},\chi_{l}^{i[2],1})\\ & \;+(\eta_{l}(\chi_{l}^{i[2],1})-\eta_{l}(\chi_{l}^{i[2],2}))\phi_{l}(\chi_{l}^{i[1],1},\chi_{l}^{i[2],1})\\ & \;+(\eta_{l}(\chi_{l}^{i[1],1})-\eta_{l}(\chi_{l}^{i[1],2}))\phi_{l}(\chi_{l}^{i[1],1},\chi_{l}^{i[2],2})\\ & \;+(\eta_{l}(\chi_{l}^{i[2],2})-\eta_{l}(\chi_{l}^{i[2],1}))\phi_{l}(\chi_{l}^{i[1],1},\chi_{l}^{i[2],2})\\ & \;+(\eta_{l}(\chi_{l}^{i[1],2})-\eta_{l}(\chi_{l}^{i[1],1})\phi_{l}(\chi_{l}^{i[1],2},\chi_{l}^{i[2],1})\\ & \;+(\eta_{l}(\chi_{l}^{i[2],1})-\eta_{l}(\chi_{l}^{i[2],2}))\phi_{l}(\chi_{l}^{i[1],2},\chi_{l}^{i[2],1})\\ & \;+(\eta_{l}(\chi_{l}^{i[1],2})-\eta_{l}(\chi^{i[1],1}))\phi_{l}(\chi_{l}^{i[1],2},\chi_{l}^{i[2],2})\\ & \;+(\eta_{l}(\chi_{l}^{i[2],2})-\eta_{l}(\chi_{l}^{i[2],1}))\phi_{l}(\chi_{l}^{i[1],2},\chi_{l}^{i[2],2})\}. \end{array}$$

Taking expectations (conditional on the pedigree) and summing over loci, we find

$$E[R_{A}^{i}R_{D}^{i}\;|\;P(t)]=\left(F_{12}-\frac{F_{112}+F_{122}}{2}\right)\frac{\sigma_{ADI}}{2}.$$

Finally, for two distinct parents, we have found that in generation *t*, conditional on the pedigree up to time *t*,

$$\begin{array}{rl} & Var(R_{A}^{i}+R_{D}^{i})\\ & \;=\left(1-\frac{F_{11}+F_{22}}{2}\right)\frac{\sigma_{A}^{2}}{2}\\ & \;+\frac{1}{4}(3F_{12}-F_{112}-F_{122}-F_{1122})(\sigma_{DI}^{2}+\iota^{*})\\ & \;+\frac{1}{4}\left(3(1-F_{12})-(F_{11}-F_{112})-(F_{22}-F_{122}\right)\\ & \;-{\tilde{F}}_{1122}-\frac{1}{2}{\tilde{F}}_{1212})\sigma_{D}^{2}\\ & \;+\left(F_{12}-\frac{F_{112}+F_{122}}{2}\right)\sigma_{ADI}-\frac{1}{4}\iota^{*}{\tilde{F}}_{1212}. \end{array}$$

We can also read off the result for when the two parents are the same from this formula. In that case

$$\begin{array}{rl} & F_{1122}=F_{11}=F_{22}=F_{112}=F_{122},\;F_{12}=\frac{1}{2}(1+F_{11}),\\ & \text{and}\;{\tilde{F}}_{1212}=1-F_{11}. \end{array}$$

Thus, $Var(R_{A}^{i}+R_{D}^{i})$ reduces to

$$(1-F_{11})\left(\frac{\sigma_{A}^{2}}{2}+\frac{3}{8}(\sigma_{DI}^{2}+\iota^{*})+\frac{1}{4}\sigma_{D}^{2}+\frac{1}{2}\sigma_{ADI}\right)-\frac{1}{4}\iota^{*}.$$

## Appendix C: Conditioning multivariate Gaussian vectors

For ease of reference, we record here a standard result for conditioning multivariate normal random vectors on their marginal values.

Theorem C1 Suppose that

$$\left[\begin{array}{c} x_{A}\\ x_{B} \end{array}\right]∼N\left(\left[\begin{array}{c} \mu_{A}\\ \mu_{B} \end{array}\right],\left[\begin{array}{cc} \Sigma_{AA} & \Sigma_{AB}\\ \Sigma_{BA} & \Sigma_{BB} \end{array}\right]\right).$$

Then

$$x_{A}|x_{B}∼N(\mu_{A}+\Sigma_{AB}\Sigma_{BB}^{-1}(x_{B}-\mu_{B}),\Sigma_{AA}-\Sigma_{AB}\Sigma_{BB}^{-1}\Sigma_{BA}).$$

The proof can be found, for example, in Brockwell (1996, Proposition 1.3.1 in Appendix A).

## Appendix D: Generalized central limit theorems

We shall exploit known techniques for proving both convergence to a normal distribution, and for establishing the rate of convergence, in situations which go beyond the classical setting of independent identically distributed random variables. For convenience we recall the key results that we need here.

We begin with a result of Rinott (1994) on the rate of convergence in a generalized Central Limit Theorem; generalized because the summands are not identically distributed and it allows some dependence between elements in the sum. We do not use this second feature here, but it would be needed to extend our results to include effects that depend on more than one locus, and so for completeness we include it in the statement of the result. It also gives an idea of how quickly the rate of convergence deteriorates if one includes epistasis or higher order dominance effects. This result can be used both to prove asymptotic normality when we condition only on the pedigree (and not on any observed trait values), and to prove asymptotic normality of the residuals (that is the part of the trait distribution within families that is not shared among offspring) conditional on the observed traits of ancestors in the pedigree.

The dependence is captured by a *dependency graph*.

Definition D1 Let $\left\{X_{l};l\in V\right\}$ be a collection of random variables. The graph G=(V,E), where V and E denote the vertex set and edge set respectively, is said to be a *dependency graph* for the collection if for any pair of disjoint subsets $A_{1}$ and $A_{2}$ of V such that no edge in E has one endpoint in $A_{1}$ and the other in $A_{2}$, the sets of random variables $\left\{X_{l};l\in A_{1}\right\}$ and $\left\{X_{l};l\in A_{2}\right\}$ are independent.

The degree of a vertex in the graph is the number of edges connected to it and the maximal degree of the graph is just the maximum of the degrees of the vertices in it.

Theorem D2
Rinott 1994, Theorem 2.2) Let $E_{1},\ldots ,E_{M}$ be random variables having a dependency graph whose maximal degree is strictly less than *D*, satisfying $|E_{l}-E[E_{l}]|\leq B$ a.s., l=1,…,M, $E[\sum_{l=1}^{M}E_{l}]=\lambda$ and $Var(\sum_{l=1}^{M}E_{l})=\sigma^{2}>0$. Then, for every w∈R,

$$\begin{array}{rl} & \left|P\left[\frac{\sum_{l=1}^{M}E_{l}-\lambda }{\sigma }\leq w\right]-N(w)\right|\\ & \;\leq \frac{1}{\sigma }\left\{\sqrt{\frac{1}{2\pi }}DB+16{\left(\frac{M}{\sigma^{2}}\right)}^{1/2}D^{3/2}B^{2}+10\left(\frac{M}{\sigma^{2}}\right)D^{2}B^{3}\right\}, \end{array}$$

where N is the distribution function of a standard normal random variable.

In particular, when *D* and *B* are order one and $\sigma^{2}$ is of order *M*, the bound is of order $1/\sqrt{M}$.

Since we are only allowing for dominance effects that depend on allelic states at a single locus, and we have no epistasis, our dependency graphs will have no edges and so the maximal degree of any vertex will be zero and we may take D=1. Epistasis or higher order dominance effects, will increase the degree. This bound on the accuracy of the normal approximation will decrease rapidly as the number of combinations through which the allelic state at a single locus can influence the trait grows.

### Exchangeable pairs

In order to prove the asymptotic normality of the part of the trait value that is shared by all the offspring in a family conditional on parental traits, we require a different approach. Because we are conditioning on the trait values of the parents, there will be weak dependence between all the pairs of loci within the sums defining $A^{i}+D^{i}$ (and so the dependency graph for the summands would be the complete graph). To check that nonetheless the limit is Gaussian we shall use a variant of Stein’s method of exchangeable pairs, originally introduced in Stein (1986).

Recall that the pair of random variables $\left(W,W^{\prime }\right)$ is called an exchangeable pair if their joint distribution is symmetric. Suppose that E[W]=0, $E[W^{2}]=1$, $\left(W,W^{\prime }\right)$ is an exchangeable pair and

$$E[W-W^{\prime }|W]=\lambda (W-R),$$

for some 0<λ<1, where *R* is a random variable of small order.

Let us write $\Delta =W-W^{\prime }$ and define

$$\hat{K}(t)=\frac{\Delta }{2\lambda }(1_{\left\{-\Delta \leq t\leq 0\right\}}-1_{\left\{0\leq t\leq -\Delta \right\}}).$$

Note that $\int_{-\infty }^{\infty }\hat{K}(t)\;dt=\Delta^{2}/(2\lambda )$. In this case, one can show (see Chen *et al.* 2011, §2.3) that

(D1)
$$E[Wf(W)]=E\left[\int_{-\infty }^{\infty }f^{\prime }(W+t)\hat{K}(t)\;dt\right]+E[Rf(W)].$$

Proposition D3
Chen *et al.* 2011, Proposition 2.4i) Let *h* be an absolutely continuous function with $\|h^{\prime }\|<\infty$, and F any σ-algebra containing σ(W). If Equation (D1) holds, then

$$|E[h(W)]-N(h)|\leq \|h^{\prime }\|\left(\sqrt{\frac{2}{\pi }}E[|1-{\hat{K}}_{1}|]+2E[{\hat{K}}_{2}]+2E[|R|]\right),$$

where

$$\begin{array}{rl} & {\hat{K}}_{1}=E\left[\int_{-\infty }^{\infty }\hat{K}(t)\;dt\;|F\right]=E\left[\frac{\Delta^{2}}{2\lambda }\;|F\right],\\ \text{and} & {\hat{K}}_{2}=\int_{-\infty }^{\infty }|t\hat{K}(t)|\;dt=\frac{|\Delta {|}^{3}}{4\lambda }. \end{array}$$

Corollary D4 Suppose that $\left(W,W^{\prime }\right)$ is an exchangeable pair with $E[W]=\mu_{W}$ and $Var(W)=\sigma_{W}^{2}$ with

(D2) $$E[W^{\prime }|W]=(1-\lambda )W+\lambda E[W]-\lambda R$$

where *R* is a random variable of small order. Then defining ${\hat{K}}_{1}$, ${\hat{K}}_{2}$, *h* and F as in Proposoition D3,

(D3) $$\begin{array}{rl} \left|E[h(W)]-N_{\mu_{W},\sigma_{W}^{2}}(h)\right| & \leq \|h^{\prime }\|\left(\sqrt{\frac{2}{\pi }}\frac{1}{\sigma_{W}}E[|\sigma_{W}^{2}-{\hat{K}}_{1}|\right]\\ & \;+\frac{2}{\sigma_{W}^{2}}E[{\hat{K}}_{2}]+2E[|R|]), \end{array}$$

where $N_{\mu_{W},\sigma_{W}^{2}}$ denotes the distribution of a normal random variable with mean $\mu_{W}$ and variance $\sigma_{W}^{2}$.

Remark D5 Although this result is enough to guarantee that *W* is asymptotically normal, because we require $\|h^{\prime }\|<\infty$, it is not enough to bound even the distance between the cumulative distribution function of *W* and that of a standard normal random variable with an error of order $1/\sqrt{M}$. To propagate our argument from one generation to the next requires convergence of the density function of the observed trait value, and once again it is our assumption that there is some environmental noise (with a smooth density) that allows us to guarantee this convergence based on the result proved here.

## Appendix E: Key lemmas

Notation E1 Throughout the rest of the appendices, to ease the notation we shall assume that the (Gaussian) environmental noise is subsumed into the trait value *Z*, so that its distribution can be assumed to have a smooth density. That is, what we call *Z* below is the observed trait $\tilde{Z}$ discussed in the main text. Moreover, when we write P[Z=z], we actually mean the density function of the distribution of $\tilde{Z}$ evaluated at the value *z* (in formula, $P[Z=z]\;:\;=\varphi_{\tilde{Z}}(z)$ with $\varphi_{\tilde{Z}}$ the density of $\tilde{Z}$). This notation allows us to cover both the case when the allelic distributions are general (potentially concentrated on a finite number of values) and the environmental component is smooth enough that the distribution of their sum is also smooth, and the case when there is no environmental noise but the scaled allelic distributions have a smooth density over [-B,B] (in which case the distribution of the genetic component *Z* is itself smooth enough for the method below to be employed).

In this section, we prove two key lemmas which will underpin our proof. They will allow us to estimate the effect on the distribution of the allelic types at a particular locus, or particular pair of loci, of knowing the trait value. We shall be using Bayes’ rule. With a slight abuse of notation

$$\begin{array}{rl} & P[(\chi_{l}^{1},\chi_{l}^{2})=(x,x^{\prime })|Z=z]\\ & \;=\frac{P[Z=z|(\chi_{l}^{1},\chi_{l}^{2})=(x,x^{\prime })]}{P[Z=z]}P[(\chi_{l}^{1},\chi_{l}^{2})=(x,x^{\prime })]. \end{array}$$

Let us write $\Psi_{l}(x,x^{\prime })=\eta_{l}(x)+\eta_{l}(x^{\prime })+\phi_{l}(x,x^{\prime })$ and $Z_{-l}$ for the trait value of an individual with the effect of locus *l* removed, then the ratio in this expression becomes

$$\frac{P[Z_{-l}=z-\Psi_{l}(x,x^{\prime })]}{P[Z=z]}.$$

Of course, this ratio of probabilities should be interpreted as a ratio of density functions. Moreover, bearing in mind our remarks on environmental noise, we are going to suppose that these density functions are sufficiently smooth that we can justify an application of Taylor’s Theorem. Of course, we know that $Z_{-l}$ is approximately normally distributed, using exactly the same argument as for *Z*, and it is no surprise that the ratio differs from one by something of order $1/\sqrt{M}$. The importance of the next lemma will become evident when we sum conditional expectations over loci; c.f. Remark E5.

Lemma E2 In the notation above,

$$\begin{array}{rl} & P[Z_{-l}=z]\\ & \;=P[Z=z]+\frac{1}{\sqrt{M}}E[\Psi_{l}(\chi_{l}^{1},\chi_{l}^{2})]\frac{d}{dz}P[Z=z]\\ & \;+\frac{1}{M}E[\Psi_{l}(\chi_{l}^{1},\chi_{l}^{2}){]}^{2}\frac{d^{2}}{dz^{2}}P[Z=z]\\ & \;-\frac{1}{2M}E[\Psi_{l}(\chi_{l}^{1},\chi_{l}^{2}{)}^{2}]\frac{d^{2}}{dz^{2}}P[Z=z]+C_{l}(z)\frac{1}{M^{3/2}}, \end{array}$$

where the function $C_{l}(z)$ in the error term can be bounded independent of *l* and *z*.

Remark E3 Conditioning on the pedigree) Although we have suppressed it in the notation, this lemma holds in any generation, but the expressions $E[\Psi (\chi_{l}^{1},\chi_{l}^{2}){]}^{2}$ and $E[\Psi (\chi_{l}^{1},\chi_{l}^{2}{)}^{2}]$ should be interpreted as being calculated conditional on the pedigree (which will determine the probability of identity of $\chi_{l}^{1}$, $\chi_{l}^{2}$).

Proof of Lemma E2 We are going to abuse notation (still further) and imagine that $P[\chi_{l}^{1}=x,\chi_{l}^{2}=x^{\prime },Z=z]$ has a density with respect to *x*, $x^{\prime }$. Of course, we do not expect that to be true (even with environmental noise), but it makes our expressions easier to parse than using a more mathematically accurate notation. We begin with an application of Taylor’s Theorem (with respect to *z*):

(E1) $$\begin{array}{rl} & P[Z_{-l}=z]\\ & \;=\int \int P\left[\chi_{l}^{1}=x,\chi_{l}^{2}=x^{\prime },Z=z+\frac{1}{\sqrt{M}}\Psi_{l}(x,x^{\prime })\right]dx\;dx^{\prime } \end{array}$$

(E2) $$\;=\int \int P[\chi_{l}^{1}=x,\chi_{l}^{2}=x^{\prime },Z=z]\;dx\;dx^{\prime }$$

(E3) $$\begin{array}{rl} & \;+\frac{1}{\sqrt{M}}\int \int \Psi_{l}(x,x^{\prime })\frac{\partial }{\partial z}P[\chi_{l}^{1}=x,\chi_{l}^{2}=x^{\prime },Z=z]\;dx\;dx^{\prime }\\ & \;+\frac{1}{2M}\int \int \Psi_{l}(x,x^{\prime }{)}^{2}\frac{\partial^{2}}{\partial z^{2}}P[\chi_{l}^{1}=x,\chi_{l}^{2}=x^{\prime },Z=z]\;dx\;dx^{\prime } \end{array}$$

(E4) $$\;+{\hat{C}}_{l}(z)\frac{1}{M^{3/2}}.$$

Provided that P[Z=z] has a uniformly bounded third derivative, our assumption that the terms that make up $\Psi_{l}$ are uniformly bounded allows us to deduce that ${\hat{C}}_{l}$ is uniformly bounded in *l* and *z*. Notice that the expression in Equation (E2) is just P[Z=z]. Since we are not conditioning on any trait values in the pedigree, and the ancestral population is assumed to be in linkage equilibrium, $\left(\chi_{l}^{1},\chi_{l}^{2}\right)$ and $Z_{-l}$ are independent. Combining this observation with Equation (E1), and, once again applying Taylor’s Theorem, we find

P[χl1=x,χl2=x′,Z=z]=P[χl1=x,χl2=x′]P[Z−l=z−1MΨl(x,x′)]=P[χl1=x,χl2=x′]∫∫P[χl1=y,χl2=y′,1MZ=z−1MΨl(x,x′)+1MΨl(y,y′)]dydy′=P[χl1=x,χl2=x′]{P[Z=z]1M+1M∫∫(Ψ(y,y′)−Ψ(x,x′))×∂∂zP[χl1=y,χl2=y′,Z=z]dydy′+C~l(x,x′,z)1M},

where the function ${\tilde{C}}_{l}$ in the last line is uniformly bounded independent of *l* and $\left(x,x^{\prime },z\right)$. (To justify this last statement, recall that we are abusing notation and implicitly subsuming the environmental noise into the distribution of *Z*. The density function here is actually a convolution of that of the environmental noise, which is smooth, and the true distribution of *Z*, and is therefore smooth.) Still assuming sufficient regularity, differentiating the previous equation we find

(E5) $$\begin{array}{rl} & \frac{\partial }{\partial z}P[\chi_{l}^{1}=x,\chi_{l}^{2}=x^{\prime },Z=z]\\ & \;=P[\chi_{l}^{1}=x,\chi_{l}^{2}=x^{\prime }]\left\{\frac{d}{dz}P[Z=z]+\frac{1}{\sqrt{M}}\int \int (\Psi (y,y^{\prime }\right)\\ & \;-\Psi (x,x^{\prime }))\frac{\partial^{2}}{\partial z^{2}}P[\chi_{l}^{1}=y,\chi_{l}^{2}=y^{\prime },Z=z]\;dy\;dy^{\prime }\\ & \;+\frac{\partial }{\partial z}{\tilde{C}}_{l}(x,x^{\prime },z)\frac{1}{M}\}, \end{array}$$

and

(E6) $$\begin{array}{rl} & \frac{\partial^{2}}{\partial z^{2}}P[\chi_{l}^{1}=x,\chi_{l}^{2}=x^{\prime },Z=z]\\ & \;=P[\chi_{l}^{1}=x,\chi_{l}^{2}=x^{\prime }]\left\{\frac{d^{2}}{dz^{2}}P[Z=z]+\frac{1}{\sqrt{M}}\frac{\partial^{2}}{\partial z^{2}}{\tilde{\tilde{C}}}_{l}(x,x^{\prime },z)\right\}, \end{array}$$

with $\frac{\partial }{\partial z}{\tilde{C}}_{l}$ and $\frac{\partial^{2}}{\partial z^{2}}{\tilde{\tilde{C}}}_{l}$ uniformly bounded. Finally, substituting Equations (E5) and (E6) in Equations (E3) and (E4), we obtain

$$\begin{array}{rl} & P[Z_{-l}=z]=P[Z=z]\\ & \;+\frac{1}{\sqrt{M}}\frac{d}{dz}P[Z=z]\int \int \Psi_{l}(x,x^{\prime })P[\chi_{l}^{1}=x,\chi_{l}^{2}=x^{\prime }]\;dx\;dx^{\prime }\\ & \;+\frac{1}{M}\frac{d^{2}}{dz^{2}}P[Z=z]\int \int \int \int (\Psi_{l}(y,y^{\prime })-\Psi_{l}(x,x^{\prime }))\Psi_{l}(x,x^{\prime })\\ & \;\times P[\chi_{l}^{1}=y,\chi_{l}^{2}=y^{\prime }]P[\chi_{l}^{1}=x,\chi_{l}^{2}=x^{\prime }]\;dy\;dy^{\prime }\;dx\;dx^{\prime }\\ & \;+\frac{1}{2M}\frac{d^{2}}{dz^{2}}P[Z=z]\int \int \Psi_{l}(x,x^{\prime }{)}^{2}P[\chi_{l}^{1}=x,\chi_{l}^{2}=x^{\prime }]\;dx\;dx^{\prime }\\ & \;+{\hat{C}}_{l}(z)\frac{1}{M^{3/2}}\\ & \;=P[Z=z]+\frac{1}{\sqrt{M}}E[\Psi_{l}(\chi_{l}^{1},\chi_{l}^{2})]\frac{d}{dz}P[Z=z]\\ & \;+\frac{1}{M}E[\Psi_{l}(\chi_{l}^{1},\chi_{l}^{2}){]}^{2}\frac{d^{2}}{dz^{2}}P[Z=z]\\ & \;-\frac{1}{2M}E[\Psi_{l}(\chi_{l}^{1},\chi_{l}^{2}{)}^{2}]\frac{d^{2}}{dz^{2}}P[Z=z]+{\hat{C}}_{l}(z)\frac{1}{M^{3/2}}, \end{array}$$

as required.  □

We also require an analog of Lemma E2 with which to control the effect of conditioning on the trait value on the distribution of the allelic values at pairs of loci. We write $Z_{-l-m}=Z-\frac{1}{\sqrt{M}}(\Psi_{l}(\chi_{l}^{1},\chi_{l}^{2})+\Psi_{m}(\chi_{m}^{1},\chi_{m}^{2}))$ for the trait value with the contributions from loci *l* and *m* removed. The following lemma follows on iterating the argument that gave us Lemma E2.

Lemma E4 In the notation above,

$$\begin{array}{rl} & P[Z_{-l-m}=z]=P[Z=z]\\ & \;+\frac{1}{\sqrt{M}}(E[\Psi_{l}(\chi_{l}^{1},\chi_{l}^{2})]+E[\Psi_{m}(\chi_{m}^{1},\chi_{m}^{2})])\frac{d}{dz}P[Z=z]\\ & \;+\left\{\frac{1}{M}E[\Psi_{l}(\chi_{l}^{1},\chi_{l}^{2}){]}^{2}-\frac{1}{2M}E[\Psi_{l}(\chi_{l}^{1},\chi_{l}^{2}{)}^{2}\right]\\ & \;+\frac{1}{M}E[\Psi_{l}(\chi_{l}^{1},\chi_{l}^{2})]E[\Psi_{m}(\chi_{m}^{1},\chi_{m}^{2})]\\ & \;+\frac{1}{M}E[\Psi_{m}(\chi_{m}^{1},\chi_{m}^{2}){]}^{2}-\frac{1}{2M}E[\Psi_{m}(\chi_{m}^{1},\chi_{m}^{2}{)}^{2}]\}\frac{d^{2}}{dz^{2}}P[Z=z]\\ & \;+C_{l,m}(z)\frac{1}{M^{3/2}}, \end{array}$$

where the functions $C_{l,m}(z)$ are uniformly bounded in *l*, *m*, *z*.

Proof of Lemma E4 We iterate the previous result:

$$\begin{array}{rl} & P[Z_{-l-m}=z]=P[Z_{-l}=z]\\ & \;+\frac{1}{\sqrt{M}}E[\Psi_{m}(\chi_{m}^{1},\chi_{m}^{2})]\frac{d}{dz}P[Z_{-l}=z]\\ & \;+\frac{1}{M}E[\Psi_{m}(\chi_{m}^{1},\chi_{m}^{2}){]}^{2}\frac{d^{2}}{dz^{2}}P[Z_{-l}=z]\\ & \;-\frac{1}{2M}E[\Psi_{m}(\chi_{m}^{1},\chi_{m}^{2}{)}^{2}]\frac{d^{2}}{dz^{2}}P[Z_{-l}=z]+C_{m}(z)\frac{1}{M^{3/2}}; \end{array}$$

now substitute for $P[Z_{-l}=z]$ and its derivatives.  □

Remark E5 Just as for Lemma E2, the proof of Lemma E4 applies in any generation as long as one interprets the expectations as being taken conditional on the pedigree. We have assumed that our base population is in linkage equilibrium to write $E[\Psi_{l}(y,y^{\prime })\Psi_{m}(x,x^{\prime })]=E[\Psi_{l}(y,y^{\prime })]E[\Psi_{m}(x,x^{\prime })]$.

We shall only be presenting the detailed proofs for individuals in generation one. To extend to the general case requires an analog of Lemma E2 when we consider the trait values of the two parents of an individual. For completeness, we record that lemma here.

Lemma E6 Let us use $P[z_{1},z_{2}]$ to denote $P[Z^{i[1]}=z_{1},Z^{i[2]}=z_{2}]$. In the following expression, all expectations should be interpreted as taken conditional on the pedigree:

$$\begin{array}{rl} & P[Z_{-l}^{i[1]}=z_{1},Z_{-l}^{i[2]}=z_{2}]-P[z_{1},z_{2}]\\ & \;=\frac{1}{\sqrt{M}}E[\Psi_{l}(\chi_{l}^{i[1],1},\chi_{l}^{i[1],2})]\frac{\partial }{\partial z_{1}}P[z_{1},z_{2}]\\ & \;+\frac{1}{\sqrt{M}}E[\Psi_{l}(\chi_{l}^{i[2],1},\chi_{l}^{i[2],2})]\frac{\partial }{\partial z_{2}}P[z_{1},z_{2}]\\ & \;+\left(\frac{1}{M}E[\Psi_{l}(\chi_{l}^{i[1],1},\chi_{l}^{i[1],2}){]}^{2}-\frac{1}{2M}E[\Psi_{l}(\chi_{l}^{i[1],1},\chi_{l}^{i[1],2}{)}^{2}]\right)\\ & \;\times \frac{\partial^{2}}{\partial z_{1}^{2}}P[z_{1},z_{2}] \end{array}$$

$$\begin{array}{rl} & \;+\left(\frac{1}{M}E[\Psi_{l}(\chi_{l}^{i[2],1},\chi_{l}^{i[2],2}){]}^{2}-\frac{1}{2M}E[\Psi_{l}(\chi_{l}^{i[2],1},\chi_{l}^{i[2],2}{)}^{2}]\right)\\ & \;\times \frac{\partial^{2}}{\partial z_{2}^{2}}P[z_{1},z_{2}]\\ & \;+\left(\frac{2}{M}E[\Psi_{l}(\chi_{l}^{i[1],1},\chi_{l}^{i[1],2})]E[\Psi_{l}(\chi_{l}^{i[2],1},\chi_{l}^{i[2],2})\right]\\ & \;-\frac{1}{M}E[\Psi_{l}(\chi_{l}^{i[1],1},\chi_{l}^{i[1],2})\Psi_{l}(\chi_{l}^{i[2],1},\chi_{l}^{i[2],2})])\\ & \;\times \frac{\partial^{2}}{\partial z_{1}\partial z_{2}}P[z_{1},z_{2}]+O\left(\frac{1}{M^{3/2}}\right). \end{array}$$

## Appendix F: Mean and variance of trait values conditional on parental traits

We remind the reader that Notation E1 remains in force.

We now turn to calculating the conditional distribution of the trait values, conditional not just on the pedigree, as we did in Appendix B, but also on the (observed) trait values in the parental generation. We spell out the details in generation one. Here already we can identify the key points, without being overwhelmed by notation. Recall that we are implicitly conditioning not on the exact trait values of the parents, but on the observed trait values when environmental noise is taken into account, so that we can assume that the distribution of parental trait values has a smooth density.

First, we calculate the conditional mean. We distinguish the case of two distinct parents and a family produced by selfing. Recall that we wrote $A^{i}+D^{i}$ for the component shared by all individuals in the family, with $A^{i}$ and $D^{i}$ defined in Equations (28) and (29).

### Generation one: mean trait value, distinct parents

Since the parents are, by assumption, unrelated, we anticipate that the expected value of the dominance component is zero, and so the expected value of the shared component $A^{i}+D^{i}$ should be the mean value of the parental traits. However, since we are conditioning on knowing the trait values, we do have some information about the allelic types, and we must verify that this does not significantly distort the expectations.

We exploit again the fact that since the parents are unrelated, their trait values (and allelic states at locus *l*) are independent. Thus,

(F1)
$$\begin{array}{rl} & P[Z^{i[1]}=z_{1},Z^{i[2]}=z_{2}\;|\;(\chi_{l}^{i[1],1},\chi_{l}^{i[1],2}\chi_{l}^{i[2],1}\chi_{l}^{i[2],2})\\ & \;=(x,x^{\prime },y,y^{\prime })]\\ & \;=P\left[Z_{-l}^{i[1]}=z_{1}-\frac{1}{\sqrt{M}}(\eta_{l}(x)+\eta_{l}(x^{\prime })+\phi_{l}(x,x^{\prime }))\right]\\ & \;\times P\left[Z_{-l}^{i[2]}=z_{2}-\frac{1}{\sqrt{M}}(\eta_{l}(y)+\eta_{l}(y^{\prime })+\phi_{l}(y,y^{\prime }))\right]. \end{array}$$

We now use Lemma E2 and Taylor’s Theorem to deduce that

$$\begin{array}{rl} & P[(\chi_{l}^{i[1],1},\chi_{l}^{i[1],2})=(x,x^{\prime })\;|\;Z^{i[1]}=z]\\ & \;=P[(\chi_{l}^{i[1],1},\chi_{l}^{i[1],2})=(x,x^{\prime })]\\ & \;\times \left\{1-\frac{1}{\sqrt{M}}(\Psi_{l}(x,x^{\prime })-E[\Psi_{l}])\frac{1}{P[Z^{i[1]}=z]}\frac{d}{dz}P[Z^{i[1]}=z\right]\\ & \;+O\left(\frac{1}{M}\right)\}, \end{array}$$

with a symmetric expression for i[2]. Integrating against this expression and using Equations (21), (22), and (23), we find, in an obvious notation,

(F2)
$$\begin{array}{rl} & E[\eta_{l}(\chi_{l}^{i[1],1})\;|\;i[1]\neq i[2],Z^{i[1]},Z^{i[2]}]\\ & \;=-\frac{1}{\sqrt{M}}\frac{P^{\prime }[Z^{i[1]}]}{P[Z^{i[1]}]}\;Var(\eta_{l}({\hat{\chi }}_{l}))+O\left(\frac{1}{M}\right). \end{array}$$

Note that approximating $P[Z^{i[1]}]$ by a normal density and ignoring the environmental component, the order 1/M terms involves $1/(\sigma_{A}^{2}+\sigma_{D}^{2})$ and $\left(Z^{i[1]}-{\bar{z}}_{0}{)}^{2}/(\sigma_{A}^{2}+\sigma_{D}^{2}\right)$, and is controlled through these quantities and our bounds on $\eta_{l}$ and $\phi_{l}$. In particular, the approximation breaks down if the genetic variance is too small or if the trait of the parent is too extreme. Multiplying by $1/\sqrt{M}$ and summing over loci and parents, we arrive at

(F3)
$$\begin{array}{rl} & E[A^{i}\;|\;i[1]\neq i[2],Z^{i[1]},Z^{i[2]}]\\ & \;=-\left(\frac{P^{\prime }[Z^{i[1]}]}{P[Z^{i[1]}]}+\frac{P^{\prime }[Z^{i[2]}]}{P[Z^{i[2]}]}\right)\frac{1}{M}\sum_{l=1}^{M}Var(\eta_{l}({\hat{\chi }}_{l}))\\ & \;+O\left(\frac{1}{\sqrt{M}}\right). \end{array}$$

Remark F1 Since we already checked that the trait $Z^{i[1]}$ is approximately normally distributed, and the same argument evidently gives that $Z_{-l}^{i[1]}$ is approximately normally distributed for each *l*, the derivation above may seem unnecessarily complex. However, in summing the terms in Equation (F2) over loci, we exploited the fact that we could pull the ratio $P^{\prime }[Z^{i[1]}]/P[Z^{i[1]}]$ outside the sum. Only then did we approximate it by the limiting normal distribution. We could only do this because we expressed everything in terms of the distribution of the whole trait. If we try to approximate the distribution of $Z_{-l}^{i[1]}$ directly by a normal distribution, and then sum, we cannot control the error. We shall use this trick repeatedly in what follows.

Similarly,

$$\begin{array}{rl} & E[\phi_{l}(\chi_{l}^{i[1],1},\chi_{l}^{i[2],1})\;|\;i[1]\neq i[2],Z^{i[1]}=z_{1},Z^{i[2]}=z_{2}]\\ & \;=\int \ldots \int \phi_{l}(x,y)\{1-\frac{1}{\sqrt{M}}(\Psi_{l}(x,x^{\prime })-E[\Psi_{l}])\frac{1}{P[Z^{i[1]}=z_{1}]}\\ & \;\times \frac{d}{dz_{1}}P[Z^{i[1]}=z_{1}]\}\\ & \;\times \left\{1-\frac{1}{\sqrt{M}}(\Psi_{l}(y,y^{\prime })-E[\Psi_{l}])\frac{1}{P[Z^{i[2]}=z_{2}]}\frac{d}{dz_{2}}P[Z^{i[2]}=z_{2}]\right\}\\ & \;\times {\hat{\nu }}_{l}(dx){\hat{\nu }}_{l}(dx^{\prime }){\hat{\nu }}_{l}(dy){\hat{\nu }}_{l}(dy^{\prime })+O\left(\frac{1}{M}\right). \end{array}$$

The terms of order one and $1/\sqrt{M}$ vanish as a result of Equations (20), (21), (22), and (23). Multiplying by $1/\sqrt{M}$ and summing over loci, we find that $E[D^{i}]=O(1/\sqrt{M})$.

Recalling that the trait distribution in the ancestral population is (almost) normally distributed with mean ${\bar{z}}_{0}$, we see that if we ignore environmental effects, so that the variance of the trait distribution in generation zero is $\sigma_{A}^{2}+\sigma_{D}^{2}$, then adding ${\bar{z}}_{0}$ to the right-hand side of Equation (F3), and substituting

$$P[Z^{i[1]}]=\frac{1}{\sqrt{2\pi (\sigma_{A}^{2}+\sigma_{D}^{2})}}\exp\;\left(-\frac{(Z^{i[1]}-{\bar{z}}_{0}{)}^{2}}{2(\sigma_{A}^{2}+\sigma_{D}^{2})}\right),$$

we recover that up to an error of order $1/\sqrt{M}$, the expected trait value among offspring is

$${\bar{z}}_{0}+\frac{\sigma_{A}^{2}}{\sigma_{A}^{2}+\sigma_{D}^{2}}\left(\frac{Z^{i[1]}+Z^{i[2]}}{2}-{\bar{z}}_{0}\right),$$

as predicted by Theorem C1.

Remark F2 The breeder’s equation) Suppose that as a result of environmental noise, the observed trait of each individual in the ancestral population is its genetic trait plus an independent $N(0,\sigma_{E}^{2})$ random variable. Then assuming normality of the ancestral trait distribution, and using Theorem C1, we find that for unrelated parents the mean trait in generation one is

(F4) $${\bar{z}}_{0}+\frac{\sigma_{A}^{2}}{\sigma_{Z}^{2}}\left(\frac{\left(Z^{i[1]}+Z^{i[2]}\right)}{2}-{\bar{z}}_{0}\right),$$

where $\sigma_{Z}^{2}$ is the total variance of the *observed* trait in the ancestral population; that is $\sigma_{Z}^{2}=\sigma_{A}^{2}+\sigma_{D}^{2}+\sigma_{E}^{2}$. Equation (F4) is the *breeder’s equation*.

### Mean trait value, same parent

We now turn to the expected trait value in a family in generation one that is produced by selfing. The calculation for the additive term is unchanged, but now we have a nontrivial contribution from the dominance component. We denote the parent $Z^{i[1]}$. Since $Z^{i[1]}=Z^{i[2]}$, we must calculate $E[\phi_{l}(\chi_{l}^{i[1],1},\chi_{l}^{i[1],1})|Z^{i[1]}]$ and $E[\phi_{l}(\chi_{l}^{i[1],1},\chi_{l}^{i[1],2})|Z^{i[1]}]$.

Our strategy is as before: we express each of these probabilities in terms of the distribution of the trait value minus the contribution from locus *l* and we apply Lemma E2. Thus, once again using that in generation zero, before conditioning, the two alleles at locus *l* in $Z^{i[1]}$ are independent draws from ${\hat{\nu }}_{l}$,

$$\begin{array}{rl} & E[\phi_{l}(\chi_{l}^{i[1],1},\chi_{l}^{i[1],1})\;|\;i[1]=i[2],Z^{i[1]}=z]\\ & \;=\int \int \phi_{l}(x,x)(1-\frac{1}{\sqrt{M}}(\Psi_{l}(x,x^{\prime })-E[\Psi_{l}])\frac{1}{P[Z^{i[1]}=z]}\\ & \;\times \frac{d}{dz}P[Z^{i[1]}=z]){\hat{\nu }}_{l}(dx){\hat{\nu }}_{l}(dx^{\prime })+O\left(\frac{1}{M}\right). \end{array}$$

Using Equations (21), (22), (23), we see that on integration the only nonzero contribution comes from the term $\eta_{l}(x)\phi_{l}(x,x)$ which can be integrated to yield

$$\begin{array}{rl} & E[\phi_{l}(\chi_{l}^{i[1],1},\chi_{l}^{i[1],1})\;|\;i[1]=i[2],Z^{i[1]}]\\ & \;=E[\phi_{l}({\hat{\chi }}_{l},{\hat{\chi }}_{l})]-\frac{1}{\sqrt{M}}\frac{P^{\prime }[Z^{i[1]}]}{P[Z^{i[1]}]}E[\eta_{l}({\hat{\chi }}_{l})\phi_{l}({\hat{\chi }}_{l},{\hat{\chi }}_{l})]+O\left(\frac{1}{M}\right). \end{array}$$

Similarly,

$$\begin{array}{rl} & E[\phi_{l}(\chi_{l}^{i[1],1},\chi_{l}^{i[1],2})\;|\;i[1]=i[2],Z^{i[1]}]\\ & \;=-\frac{1}{\sqrt{M}}\frac{P^{\prime }[Z^{i[1]}]}{P[Z^{i[1]}]}E[\phi_{l}({\hat{\chi }}_{l},{\hat{\chi }}_{2}{)}^{2}]+O\left(\frac{1}{M}\right). \end{array}$$

Multiplying by $1/\sqrt{M}$ and summing over loci, we find that the mean of the term $D^{i}$ in Equation (29), conditional on i[1]=i[2] and on knowing the trait value $Z^{i[1]}$, is

$$\begin{array}{rl} & \frac{1}{2\sqrt{M}}\;\sum_{l=1}^{M}E[\phi_{l}({\hat{\chi }}_{l},{\hat{\chi }}_{l})]-\frac{P^{\prime }[Z^{i[1]}]}{P[Z^{i[1]}]}\left(\frac{1}{2M}\sum_{l=1}^{M}E[\eta_{l}({\hat{\chi }}_{l})\phi_{l}({\hat{\chi }}_{l},{\hat{\chi }}_{l})\right]\\ & \;+\frac{1}{2M}\sum_{l=1}^{M}E[\phi_{l}({\hat{\chi }}_{l},{\hat{\chi }}_{2}{)}^{2}])+O\left(\frac{1}{\sqrt{M}}\right). \end{array}$$

Adding on the additive terms that we calculated before and restating everything in terms of the quantities in Table 1, we obtain that for two identical parents

(F5)
$$\begin{array}{rl} & E[Z^{i}|i[1]=i[2],Z^{i[1]}]\\ & \;={\bar{z}}_{0}+\left(\frac{1}{2}\iota -\frac{P^{\prime }[Z^{i[1]}]}{P[Z^{i[1]}]}\left(\sigma_{A}^{2}+\frac{\sigma_{D}^{2}}{2}+\frac{\sigma_{ADI}}{4}\right)\right)\\ & \;+O\left(\frac{1}{\sqrt{M}}\right). \end{array}$$

Notice that the factor of 1/2 in front of ι is the probability of identity $F^{*}$ of the two genes in the offspring.

Of course, there is no surprise here: $E[(A^{i}+D^{i})|i[1]=i[2]]=\iota /2$ and

(F6)
$$\begin{array}{rl} & Cov(A^{i}+D^{i},Z^{i[1]}|i[1]=i[2])\\ & \;=\frac{1}{M}\sum_{l=1}^{M}E[(\eta_{l}({\hat{\chi }}_{l}^{1})+\eta_{l}({\hat{\chi }}_{2}^{1})+\frac{\phi ({\hat{\chi }}_{l}^{1},{\hat{\chi }}_{l}^{1})+\phi ({\hat{\chi }}_{l}^{2},{\hat{\chi }}_{l}^{2})}{4}\\ & \;+\frac{\phi ({\hat{\chi }}_{l}^{1},{\hat{\chi }}_{l}^{2})}{2})(\eta ({\hat{\chi }}_{l}^{1})+\eta ({\hat{\chi }}_{l}^{2})+\phi ({\hat{\chi }}_{l}^{1},{\hat{\chi }}_{l}^{2}))]\\ & \;=\sigma_{A}^{2}+\frac{\sigma_{D}^{2}}{2}+\frac{\sigma_{ADI}}{4}. \end{array}$$

Thus, up to the error term, Equation (F5) is just

$${\bar{z}}_{0}+E[A^{i}+D^{i}]+Cov(A^{i}+D^{i},Z^{i[1]})\frac{\left(Z^{i[1]}-E[Z^{i[1]}]\right)}{Var(Z^{i[1]})},$$

as we expect from the (approximately) bivariate normal distribution of $\left(A^{i}+D^{i}\right)$ and $Z^{i[1]}$.

### Variance of the shared parental contribution $A^{i}+D^{i}$, generation one

We now turn to the variance of the shared parental contribution. This is where the complications associated with incorporating dominance really start to be felt. In the process of calculating the conditional mean above, we established that conditioning on the parental trait values (and whether or not they are identical) distorts the distribution of the allelic state at a given locus by a factor of order $1/\sqrt{M}$. This distortion is enough to shift the mean trait (as we see in the breeder’s equation), and, as we shall see, the variance of the sum over loci will have a contribution from linkage disequilibrium.

### Conditional variance $\left(A^{i}+D^{i}\right)$, generation one, same parent

First we consider the case in which the parents are the same. We need to calculate the expectation of $(A^{i}+D^{i}{)}^{2}$ conditional upon the parental trait. We begin with the “diagonal” terms, corresponding to a single locus. We take these in three parts. First, proceeding as before,

(F7)
$$\begin{array}{rl} & E[\eta_{l}(\chi_{l}^{i[1],1}{)}^{2}\;|\;i[1]=i[2],Z^{i[1]}=z]\\ & \;=\int \int \eta_{l}{\left(x\right)}^{2}\left(1-\frac{1}{\sqrt{M}}(\Psi_{l}(x,x^{\prime })-E[\Psi_{l}]\right)\\ & \;\times \frac{1}{P[Z^{i[1]}=z]}\frac{d}{dz}P[Z^{i[1]}=z]){\hat{\nu }}_{l}(dx){\hat{\nu }}_{l}(dx^{\prime })+O\left(\frac{1}{M}\right)\\ & \;=E[\eta_{l}({\hat{\chi }}_{l}{)}^{2}]+O\left(\frac{1}{\sqrt{M}}\right). \end{array}$$

Notice that the term arising from the Taylor expansion is already of order $1/\sqrt{M}$, and, since we multiply each of the terms in the sum by 1/M, we have no need to develop the expansion further. Indeed, all terms in the expression for the variance will be multiplied by 1/M and so for the “diagonal” terms in the square of the sum, we only need an expression to leading order.

Remark F3 The error that we are making in discarding the terms arising from the Taylor expansion is $1/\sqrt{M}$ multiplied by a term that depends on $P^{\prime }[Z^{i[1]}]/P[Z^{i[1]}]=-(Z^{i[1]}-E[Z^{i[1]}])/Var(Z^{i[1]})$. As usual, the approximation will be poor if the trait value of the parent is too extreme, or the variance is too small.

As a result, for these terms we can calculate with respect to the distribution in the ancestral population and we find

$$\begin{array}{rl} & \frac{1}{M}E\left[\sum_{l=1}^{M}{\left(\eta_{l}(\chi_{l}^{i[1],1})+\eta_{l}(\chi_{l}^{i[1],2})\right)}^{2}\;|i[1]=i[2],Z^{i[1]}\right]\\ & \;=\frac{2}{M}\sum_{l=1}^{M}E[\eta_{l}({\hat{\chi }}_{l}{)}^{2}]+O\left(\frac{1}{\sqrt{M}}\right)\\ & \;=\sigma_{A}^{2}+O\left(\frac{1}{\sqrt{M}}\right). \end{array}$$

Similarly, recalling that we are still considering the case of identical parents,

116ME[∑l=1M(ϕl(χli[1],1,χli[1],1)+2ϕl(χli[1],1,χli[1],2)+ϕl(χli[1],2,χli[1],2))2|i[1]=i[2],Zi[1]∑l=1M∑l=1M]=116M∑l=1M(2E[ϕl(χ^l,χ^l)2]+4E[ϕl(χ^l1,χ^l2)2])+O(1M)=18(σDI2+ι*)+14σD2+O(1M),

and

12ME[∑l=1M(ηl(χli[1],1)+ηl(χli[1],2))×(ϕl(χli[1],1,χli[1],1)+2ϕl(χli[1],1,χli[1],2)+ϕl(χli[1],2,χli[1],2))|i[1]=i[2],Zi[1]∑l=1M∑l=1M]=12ME[∑l=1M(ηl(χli[1],1)+ηl(χli[1],2))×(ϕl(χli[1],1,χli[1],1)+2ϕl(χli[1],1,χli[1],2)+ϕl(χli[1],2,χli[1],2))∑l=1M]+O(1M)

$$\begin{array}{rl} & \;=\frac{1}{2M}\sum_{l=1}^{M}2E[\eta_{l}({\hat{\chi }}_{l})\phi_{l}({\hat{\chi }}_{l},{\hat{\chi }}_{l})]+O\left(\frac{1}{\sqrt{M}}\right)\\ & \;=\frac{\sigma_{ADI}}{2}+O\left(\frac{1}{\sqrt{M}}\right). \end{array}$$

Combining all these terms we find that if the parents are identical, then the contribution to $E[(A^{i}+D^{i}{)}^{2}|i[1]=i[2],Z^{i[1]}]$ from the “diagonal” terms is

(F8)
$$\sigma_{A}^{2}+\frac{1}{8}(\sigma_{DI}^{2}+\iota^{*})+\frac{1}{4}\sigma_{D}^{2}+\frac{1}{2}\sigma_{ADI}+O\left(\frac{1}{\sqrt{M}}\right).$$

We must now turn to the contribution from correlations across loci. For this, we must compute

(F9)
1ME[∑l≠m(ηl(χli[1],1)+ηl(χli[1],2))(ηm(χmi[1],1)+ηm(χmi[1],2))|i[1]=i[2],Zi[1]∑l≠m]

(F10)
(F10)+12ME[∑l≠m(ηl(χli[1],1)+ηl(χli[1],2))(ϕm(χmi[1],1,χmi[1],1)+ϕm(χmi[1],2,χmi[1],2)+2ϕm(χmi[1],1,χmi[1],2))|i[1]=i[2],Zi[1]∑l≠m](F11)+116ME[∑l≠m(ϕl(χli[1],1,χli[1],1)+ϕl(χli[1],2,χli[1],2)+2ϕl(χli[1],1,χli[1],2))(ϕm(χmi[1],1,χmi[1],1)+ϕm(χmi[1],2,χmi[1],2)+2ϕm(χmi[1],1,χmi[1],2))|i[1]=i[2],Zi[1]∑l≠m].

This time we use Lemma E4.

(F12)
1P[Zi[1]=z]P[Zi[1]=z|(χli[1],1,χli[1],2,χmi[1],1,χmi[1],2)=(x,x′,y,y′)]=1P[Zi[1]=z]P[Z−l−mi[1]=z−1M(ηl(x)+ηl(x′)+ϕl(x,x′)+ηm(y)+ηm(y′)+ϕm(y,y′))1M]=1−1M(Ψl(x,x′)+Ψm(y,y′)−E[Ψl+Ψm])×1P[Zi[1]=z]ddzP[Zi[1]=z]+12M((Ψl(x,x′)+Ψm(y,y′))2−E[(Ψl+Ψm)2])×1P[Zi[1]=z]d2dz2P[Zi[1]=z]−1M(Ψl(x,x′)+Ψm(y,y′)−E[(Ψl+Ψm)])E[Ψl+Ψm]×1P[Zi[1]=z]d2dz2P[Zi[1]=z]+O(1M3/2).

Using that in the ancestral population we are at linkage equilibrium with $x,x^{\prime }$ and $y,y^{\prime }$ sampled independently from ${\hat{\nu }}_{l}$ and ${\hat{\nu }}_{m}$, respectively, multiplying by $\eta_{l}(x)\eta_{m}(y)$ and integrating against $\hat{\nu }(dx)\hat{\nu }(dy)$, the only nonzero term corresponds to the term $\eta_{l}(x)\eta_{m}(y)$ in $(\Psi_{l}(x,x^{\prime })+\Psi_{m}(y,y^{\prime }){)}^{2}$, so that

(F13)
1ME[∑l≠m(ηl(χli[1],1)+ηl(χli[1],2))×(ηm(χmi[1],1)+ηm(χmi[1],2))|i[1]=i[2],Zi[1]∑l≠m]=4M2P″[Zi[1]]P[Zi[1]]∑l≠mE[ηl(χ^l)2]E[ηm(χ^m)2]+O(1M)=P″[Zi[1]]P[Zi[1]](σA2)2+O(1M).

(The factor of 4 corresponds to the four possible ways of choosing the parents at the two loci.) Similarly, to calculate

$$E[\eta_{l}(\chi_{l}^{i[1],1})\phi_{m}(\chi_{m}^{i[1],1},\chi_{m}^{i[1],1})\;|\;i[1]=i[2],Z^{i[1]}]$$

we multiply Equation (F12) by $\eta_{l}(x)\phi_{m}(y,y)$ and integrate. Once again, using Equations (21)–(23), we find that most of the terms vanish, leaving only

(F14)
$$\begin{array}{rl} & -\frac{1}{\sqrt{M}}\frac{P^{\prime }[Z^{i[1]}]}{P[Z^{i[1]}]}E[\eta_{l}({\hat{\chi }}_{l}{)}^{2}]E[\phi_{m}({\hat{\chi }}_{m},{\hat{\chi }}_{m})]\\ & +\frac{1}{2M}\frac{P^{\prime \prime }[Z^{i[1]}]}{P[Z^{i[1]}]}\{E[\eta_{l}({\hat{\chi }}_{l}{)}^{3}]E[\phi_{m}({\hat{\chi }}_{m},{\hat{\chi }}_{m})]\\ & \;+E[\eta_{l}({\hat{\chi }}_{l})\phi_{l}({\hat{\chi }}_{l}^{1},{\hat{\chi }}_{l}^{2}{)}^{2}]E[\phi_{m}({\hat{\chi }}_{m},{\hat{\chi }}_{m})]\\ & \;+2E[\eta_{l}({\hat{\chi }}_{l}{)}^{2}]E[\eta_{m}({\hat{\chi }}_{m})\phi_{m}({\hat{\chi }}_{m},{\hat{\chi }}_{m})]\}. \end{array}$$

Multiplying by 1/(2M) and summing over loci, in the notation of Table 1, the first term yields

$$-\iota \sigma_{A}^{2}\frac{P^{\prime }[Z^{i[1]}]}{P[Z^{i[1]}]}.$$

[There are four terms of this form in Equation (F10) and we have taken account of all of them.] The last term gives

$$\frac{\sigma_{A}^{2}\sigma_{ADI}}{2}\frac{P^{\prime \prime }[Z^{i[1]}]}{P[Z^{i[1]}]}$$

[again counting the contribution from all four terms of this form in Equation (F10)].

Now observe that

$$\frac{1}{M}\sum_{m=1}^{M}E[\phi_{m}({\hat{\chi }}_{m},{\hat{\chi }}_{m})]=\frac{\iota }{\sqrt{M}},$$

so that summing over loci, the contribution from the first two terms multiplying the second derivative will be $O(1/\sqrt{M})$.

Remark F4 Up to this point, it has been possible to neglect the error terms under the assumption that the within-family variance is not too small and we are not too far out into the tails of the distribution of $Z^{i[1]}$; the more extreme the trait of the parent, the worse the approximation will be. Now things change. In order for $E[A^{i}+D^{i}]$ to be finite, we required that the inbreeding depression ι be well defined; here, we see that it also enters into the error terms.

In the same way, we calculate

$$E[\eta_{l}(\chi_{l}^{i[1],1})\phi_{m}(\chi_{m}^{i[1],1},\chi_{m}^{i[1],2})\;|\;i[1]=i[2],Z^{i[1]}]$$

by multipling Equation (F12) by $\eta_{l}(x)\phi_{m}(y,y^{\prime })$ and integrating. The only term to survive integration is

(F15)
$$\frac{1}{2M}\frac{P^{\prime \prime }[Z^{i[1]}]}{P[Z^{i[1]}]}E[2\eta_{l}({\hat{\chi }}_{l}{)}^{2}]E[\phi_{m}({\hat{\chi }}_{m}^{1},{\hat{\chi }}_{m}^{2}{)}^{2}].$$

There are four terms of this form in Equation (F10), each of which is weighted by 1/(2M) and so, summing over loci, we arrive at an overall contribution of $\sigma_{A}^{2}\sigma_{D}^{2}P^{\prime \prime }[Z^{i[1]}]/P[Z^{i[1]}]$. Equations (F14) and (F15) yield that Equation (F10) equals

(F16)
12ME[∑l≠m(ηl(χli[1],1)+ηl(χli[1],2))(ϕm(χmi[1],1,χmi[1],1)+ϕm(χmi[1],2,χmi[1],2)+2ϕm(χmi[1],1,χmi[1],2))|i[1]=i[2],Zi[1]∑l≠m]=−P′[Zi[1]]P[Zi[1]]ισA2+P″[Zi[1]]P[Zi[1]]{σA2σADI2+σA2σD2}+O(1M).

Continuing in this way,

$$E[\phi_{l}(\chi_{l}^{i[1],1},\chi_{l}^{i[1],1})\phi_{m}(\chi_{m}^{i[1],1},\chi_{m}^{i[1],1})\;|\;i[1]=i[2],Z^{i[1]}]$$

is obtained by multiplying Equation (F12) by $\phi_{l}(x,x)\phi_{m}(y,y)$ and integrating. When we sum the “constant” term over loci we will obtain $\iota^{2}/M$ which tends to zero. The remaining nonzero terms are

(F17)
$$\begin{array}{rl} & -\frac{1}{\sqrt{M}}\frac{P^{\prime }[Z^{i[1]}]}{P[Z^{i[1]}]}\{E[\eta_{l}({\hat{\chi }}_{l})\phi_{l}({\hat{\chi }}_{l},{\hat{\chi }}_{l})]E[\phi_{m}({\hat{\chi }}_{m},{\hat{\chi }}_{m})]\\ & \;+E[\phi_{l}({\hat{\chi }}_{l},{\hat{\chi }}_{l})]E[\eta_{m}({\hat{\chi }}_{m})\phi_{m}({\hat{\chi }}_{m},{\hat{\chi }}_{m})]\}\\ & \;+\frac{1}{2M}\frac{P^{\prime \prime }[Z^{i[1]}]}{P[Z^{i[1]}]}\{E[\phi_{l}({\hat{\chi }}_{l},{\hat{\chi }}_{l})\phi_{m}({\hat{\chi }}_{m},{\hat{\chi }}_{m})(\eta_{l}({\hat{\chi }}_{l}{)}^{2}+\eta_{m}({\hat{\chi }}_{m}{)}^{2}\\ & \;+2\eta_{l}({\hat{\chi }}_{l})\eta_{m}({\hat{\chi }}_{m}))]\\ & \;+E[\phi_{l}({\hat{\chi }}_{l}^{1},{\hat{\chi }}_{l}^{1})\phi_{m}({\hat{\chi }}_{m}^{1},{\hat{\chi }}_{m}^{1})(\eta_{l}({\hat{\chi }}_{l}^{2}{)}^{2}+\eta_{m}({\hat{\chi }}_{m}^{2}{)}^{2})]\}. \end{array}$$

The terms in the last line will contribute $O(1/\sqrt{M})$ when we sum, as will the first two terms in the middle line. There are four terms of this form in Equation (F11) and we are multiplying by 1/(16M) and summing over loci, so the top line contributes $-\iota \sigma_{ADI}P^{\prime }[Z^{i[1]}]/(4P[Z^{i[1]}])$, similarly the second line will contribute $\sigma_{ADI}^{2}P^{\prime \prime }[Z^{i[1]}]/(16P[Z^{i[1]}])$.

Now, again using Equation (F12),

$$\begin{array}{rl} & E[\phi_{l}(\chi_{l}^{i[1],1},\chi_{l}^{i[1],1})\phi_{m}(\chi_{m}^{i[1],1},\chi_{m}^{i[1],2})\;|\;i[1]=i[2],Z^{i[1]}]\\ & \;=\int \ldots \int \phi_{l}(x,x)\phi_{m}(y,y^{\prime })\left[1-\frac{1}{\sqrt{M}}(\Psi_{l}(x,x^{\prime })+\Psi_{m}(y,y^{\prime }\right)\\ & \;-E[\Psi_{l}+\Psi_{m}])\frac{P^{\prime }[Z^{i[1]}]}{P[Z^{i[1]}]}\\ & \;+\frac{1}{2M}((\Psi_{l}(x,x^{\prime })+\Psi_{m}(y,y^{\prime }){)}^{2}-E[(\Psi_{l}+\Psi_{m}{)}^{2}])\frac{P^{\prime \prime }[Z^{i[1]}]}{P[Z^{i[1]}]}\\ & \;-\frac{1}{M}(\Psi_{l}(x,x^{\prime })+\Psi_{m}(y,y^{\prime })-E[(\Psi_{l}+\Psi_{m})])E[\Psi_{l}+\Psi_{m}]\\ & \;\times \frac{P^{\prime \prime }[Z^{i[1]}]}{P[Z^{i[1]}]}]{\hat{\nu }}_{l}(dx){\hat{\nu }}_{l}(dx^{\prime }){\hat{\nu }}_{m}(dy){\hat{\nu }}_{m}(dy^{\prime })\\ & \;+O(\frac{1}{M^{3/2}}). \end{array}$$

There are eight terms of this form in Equation (F11), and we are multiplying by 1/(16M) and summing over loci, so the first term will correspond to a contribution of

$$-\frac{P^{\prime }[Z^{i[1]}]}{P[Z^{i[1]}]}\frac{\iota \sigma_{D}^{2}}{2}.$$

As usual, terms multiplying the second derivative that involve the locus *l* only through $\phi_{l}(x,x)$ will contribute $O(1/\sqrt{M})$ to the sum and we find that the nontrivial contributions will be

$$\begin{array}{rl} & -\frac{1}{\sqrt{M}}\frac{P^{\prime }[Z^{i[1]}]}{P[Z^{i[1]}]}E[\phi_{l}({\hat{\chi }}_{l},{\hat{\chi }}_{l})]E[\phi_{m}({\hat{\chi }}_{m}^{1},{\hat{\chi }}_{m}^{2}{)}^{2}]\\ & +\frac{1}{2M}\frac{P^{\prime \prime }[Z^{i[1]}]}{P[Z^{i[1]}]}E[2\eta_{l}({\hat{\chi }}_{l})\phi_{l}({\hat{\chi }}_{l},{\hat{\chi }}_{l})]E[\phi_{m}({\hat{\chi }}_{m}^{1},{\hat{\chi }}_{m}^{2}{)}^{2}]. \end{array}$$

There are eight terms of this form in Equation (F11), so multiplying by 1/(16M) and summing over loci gives

(F18)
$$-\frac{\iota \sigma_{D}^{2}}{2}\frac{P^{\prime }[Z^{i[1]}]}{P[Z^{i[1]}]}+\frac{\sigma_{ADI}\sigma_{D}^{2}}{4}\frac{P^{\prime \prime }[Z^{i[1]}]}{P[Z^{i[1]}]}.$$

Finally, when we scale and sum over loci, the only nontrivial term in our expression for

$$E[\phi_{l}(\chi_{l}^{i[1],1},\chi_{l}^{i[1],2})\phi_{m}(\chi_{m}^{i[1],1},\chi_{m}^{i[1],2})\;|\;i[1]=i[2],Z^{i[1]}]$$

is

$$+\frac{1}{2M}\frac{P^{\prime \prime }[Z^{i[1]}]}{P[Z^{i[1]}]}E[2\phi_{l}({\hat{\chi }}_{l}^{1},{\hat{\chi }}_{l}^{2}{)}^{2}]E[\phi_{m}({\hat{\chi }}_{m}^{1},{\hat{\chi }}_{m}^{2}{)}^{2}].$$

There are four terms of this form, and so multiplying by 1/(16M) and summing gives

(F19)
$$\frac{(\sigma_{D}^{2}{)}^{2}}{4}\frac{P^{\prime \prime }[Z^{i[1]}]}{P[Z^{i[1]}]}.$$

Combining Equations (F17), (F18), and (F19), we find that Equation (F11) is

(F20)
116ME[∑l≠m(ϕl(χli[1],1,χli[1],1)+(ϕl(χli[1],2,χli[1],2)+2ϕl(χli[1],1,χli[1],2))(ϕm(χmi[1],1,χmi[1],1)+(ϕm(χmi[1],2,χmi[1],2)+2ϕm(χmi[1],1,χmi[1],2))|i[1]=i[2],Zi[1]∑l≠m]=−P′[Zi[1],1]P[Zi[1],1](ισADI4+ισD22)+P″[Zi[1],1]P[Zi[1],1](σADI216+σADIσD24+(σD2)24)+O(1M).

Adding Equations (F8), (F13), (F16), and (F20) yields $E[(A^{i}+D^{i}{)}^{2}]$, and subtracting the square of Equation (F5), we obtain

$$\begin{array}{rl} & Var(A^{i}+D^{i}|i[1]=i[2],Z^{i[1]})\\ & \;=\sigma_{A}^{2}+\frac{1}{8}(\sigma_{DI}^{2}+\iota^{*})+\frac{1}{4}\sigma_{D}^{2}+\frac{1}{2}\sigma_{ADI}\\ & \;-\frac{P^{\prime }[Z^{i[1]}]}{P[Z^{i[1]}]}\left\{\iota \sigma_{A}^{2}+\frac{\iota \sigma_{ADI}}{4}+\frac{\iota \sigma_{D}^{2}}{2}\right\}\\ & \;+\frac{P^{\prime \prime }[Z^{i[1]}]}{P[Z^{i[1]}]}\{(\sigma_{A}^{2}{)}^{2}+\frac{\sigma_{A}^{2}\sigma_{ADI}}{2}+\sigma_{A}^{2}\sigma_{D}^{2}\\ & \;+\frac{(\sigma_{D}^{2}{)}^{2}}{4}+\frac{\sigma_{ADI}^{2}}{16}+\frac{\sigma_{D}^{2}\sigma_{ADI}}{4}\}\\ & \;-{\left(\frac{\iota }{2}-\frac{P^{\prime }[Z^{i[1]}]}{P[Z^{i[1]}]}\left(\sigma_{A}^{2}+\frac{\sigma_{D}^{2}}{2}+\frac{\sigma_{ADI}}{4}\right)\right)}^{2}+O\left(\frac{1}{\sqrt{M}}\right). \end{array}$$

Now if we substitute the Gaussian density for $Z^{i[1]}$, observing that

$$\frac{P^{\prime \prime }[Z^{i[1]}]}{P[Z^{i[1]}]}-{\left(\frac{P^{\prime }[Z^{i[1]}]}{P[Z^{i[1]}]}\right)}^{2}=-\frac{1}{\sigma_{A}^{2}+\sigma_{D}^{2}},$$

we see that the variance reduces to

(F21)
$$\begin{array}{rl} & -\frac{\iota^{2}}{4}+\frac{1}{\sigma_{A}^{2}+\sigma_{D}^{2}}{\left(\sigma_{A}^{2}+\frac{\sigma_{D}^{2}}{2}+\frac{\sigma_{ADI}}{4}\right)}^{2}+\sigma_{A}^{2}\\ & +\frac{1}{8}\left(\sigma_{DI}^{2}+\iota^{*}\right)+\frac{1}{4}\sigma_{D}^{2}+\frac{1}{2}\sigma_{ADI}+O\left(\frac{1}{\sqrt{M}}\right). \end{array}$$

Again, that was a lot of work to recover exactly the expression that we expected from conditioning the multivariate normal random variable $\left((A^{i}+D^{i}),Z^{i[1]}\right)$ on its second argument. However, in the process, we have identified where the normal approximation to the conditioned process will break down. The bounds that we have obtained will be poor if the trait value of either parent is too extreme, or if the pedigree is too inbred (as a result of which the variance of trait values will be small and inbreeding depression may be high).

Of course, we have not proved that the conditional distribution of $\left(A^{i}+D^{i}\right)$ converges to a normal, we have just checked that the first two moments are asymptotically what we would expect. We defer the proof of normality until we have calculated the conditional variance of $\left(A^{i}+D^{i}\right)$ in the (much simpler) case of two distinct parents.

### Conditional variance $\left(A^{i}+D^{i}\right)$, generation one, distinct parents

If the parents are distinct, then the expressions are much simpler. First

14ME[∑l=1M(ηl(χli[1],1)+ηl(χli[1],2)+ηl(χli[2],1)+ηl(χli[2],2))2|i[1]≠i[2],Zi[1],Zi[2]∑l=1M]=1M∑l=1ME[ηl(χ^l)2]+O(1M)=σA22+O(1M).

Next

116ME[∑l=1M(ϕl(χli[1],1,χli[2],1)+ϕl(χli[1],1,χli[2],2)+ϕl(χli[1],2,χli[2],1)+ϕl(χli[1],2,χli[2],2))2|i[1]≠i[2],Zi[1],Zi[2]∑l=1M]=14M∑l=1ME[ϕl(χ^l1,χ^l2)2]+O(1M)=14σD2+O(1M).

Finally,

14ME[∑l=1M(ηl(χli[1],1)+ηl(χli1,2)+ηl(χli[2],1)+ηl(χli[2],2))×(ϕl(χli[1],1,χli[2],1)+ϕl(χli[1],1,χli[2],2)+ϕl(χli[1],2,χli[2],1)ϕl(χli[1],2,χli[2],2))|i[1]≠i[2],Zi[1],Zi[2]∑l=1M]=O(1M).

We now turn to the off-diagonal terms. We need to be able to calculate the conditional expectation of

(F22)
[12(ηl(χli[1],1)+ηl(χli[1],2)+ηl(χli[2],1)+ηl(χli[2],2))+14(ϕl(χli[1],1,χli[2],1)+ϕl(χli[1],1,χli[2],2)+ϕl(χli[1],2,χli[2],1)+ϕl(χli[1],2,χli[2],2))12]×[12(ηm(χmi[1],1)+ηm(χmi[1],2)+ηm(χmi[2],1)+ηm(χmi[2],2))+14(ϕm(χmi[1],1,χmi[2],1)+ϕm(χmi[1],1,χmi[2],2)+ϕm(χmi[1],2,χmi[2],1)+ϕm(χmi[1],2,χmi[2],2))12]

given the trait values in the (unrelated) parents i[1] and i[2]. Because the parents are distinct, and they are in generation zero, as in Equation (F1) in our calculation of the conditional mean, we can exploit the fact that the trait values $Z^{i[1]}$ and $Z^{i[2]}$ are independent so that the joint probability that

$$(\chi_{l}^{i[1],1},\chi_{l}^{i[1],2},\chi_{m}^{i[1],1},\chi_{m}^{i[1],2})=(x,x^{\prime },y,y^{\prime }),$$

conditional on $Z^{i[1]},Z^{i[2]}$, is just the same as if we only condition on $Z^{i[1]}$. Recalling Equation (F1), we can calculate the conditional expectation of Equation (F22) using Equation (F12). None of the genes at either locus are identical by descent, and so integrating against the term of order $1/\sqrt{M}$ in the Taylor expansion in Equation (F12) gives zero, but since we are calculating the conditional expectation of $O(M^{2})$ terms, each of which is of order 1/M, we can expect to see a contribution from the term of order 1/M. All the terms involving the dominance components vanish, as do those terms involving only one copy of the additive component at one of the loci. In total we find that the conditional expectation of Equation (F22) is

$$\begin{array}{rl} & \frac{1}{M}E[\eta_{l}({\hat{\chi }}_{l}{)}^{2}]E[\eta_{m}({\hat{\chi }}_{m}{)}^{2}]\\ & \;\times \left\{\frac{P^{\prime \prime }[Z^{i[1]}]}{P[Z^{i[1]}]}+\frac{P^{\prime \prime }[Z^{i[2]}]}{P[Z^{i[2]}]}+2\frac{P^{\prime }[Z^{i[1]}]}{P[Z^{i[1]}]}\frac{P^{\prime }[Z^{i[2]}]}{P[Z^{i[2]}]}\right\}. \end{array}$$

Summing over loci (and noting that we may include the diagonal terms and only incur an error of order 1/M), we find that, in the case of different parents, the variance of the shared terms $A^{i}+D^{i}$, conditional on the trait values of the parent is

$$\begin{array}{rl} & \frac{1}{2}\sigma_{A}^{2}+\frac{1}{4}\sigma_{D}^{2}-{\left(\left(\frac{P^{\prime }(Z^{i[1]})}{P(Z^{i[1]})}+\frac{P^{\prime }(Z^{i[2]})}{P(Z^{i[2]})}\right)\frac{\sigma_{A}^{2}}{2}\right)}^{2}\\ & \;+\left\{\frac{P^{\prime \prime }[Z^{i[1]}]}{P[Z^{i[1]}]}+\frac{P^{\prime \prime }[Z^{i[2]}]}{P[Z^{i[2]}]}+2\frac{P^{\prime }[Z^{i[1]}]}{P[Z^{i[1]}]}\frac{P^{\prime }[Z^{i[2]}]}{P[Z^{i[2]}]}\right\}{\left(\frac{\sigma_{A}^{2}}{2}\right)}^{2}\\ & \;+O\left(\frac{1}{\sqrt{M}}\right). \end{array}$$

Once again we see that if we approximate the distribution of $Z^{i[1]}$ and $Z^{i[2]}$ by that of independent normal random variables with mean ${\bar{z}}_{0}$ and variance $\sigma_{A}^{2}+\sigma_{D}^{2}$, most of these terms cancel and we are left with

$$\frac{\sigma_{A}^{2}}{2}+\frac{\sigma_{D}^{2}}{4}-\frac{\sigma_{A}^{4}}{2(\sigma_{A}^{2}+\sigma_{D}^{2})},$$

exactly as predicted by Theorem C1.

#### The general case

So far we have only dealt with generation one, where expressions are simplified by the fact that $Z^{i[1]}$, $Z^{i[2]}$ are either identical or independent. More generally, we can perform entirely analogous calculations using Lemma E6 in place of Lemma E2. In the interest of sanity, we omit the details.

## Appendix G: Convergence to normal of $\left(A^{i}+D^{i}\right)$ conditional on parental traits

Notation G1 We remind the reader that Notation E1 remains in force. Moreover, since the environmental noise $E^{i}$ is assumed to be shared by all offspring of the couple i[1], i[2], with this convention we can also assume that the distribution of $A^{i}+D^{i}$ has a smooth density.

We have verified that the first two moments of the conditional distribution converge to the limits that we would expect if the limit of $\left(A^{i}+D^{i}\right)$ were multivariate normal, but this is not sufficient. To prove that the conditional distribution is indeed asymptotically normal, we appeal to Proposition D3, or rather Corollary D4. We perform the calculation in the case of identical parents, the case of distinct parents being analogous (and less surprising). For definiteness, we consider only generation one. The same argument will work in any generation, but the calculations become considerably more involved, c.f. Lemma E6.

Recall that $A^{i}+D^{i}=\sum_{l=1}^{M}\Phi (l)/\sqrt{M}$ with Φ defined in Equation (B1). Since we are considering the case of a single parent, $Z^{i[1]}=Z^{i[2]}$. We shall write $\Phi_{l}(\chi_{l}^{1},\chi_{l}^{2})$ when we need to specify the alleles at locus *l* in $Z^{i[1]}$ on which this is evaluated.

Writing $W=\sum_{l=1}^{M}\Phi (l)/\sqrt{M}$ (as a shorthand for $A^{i}+D^{i}$), we write

$$\hat{W}=\frac{1}{\sqrt{M}}\sum_{l=1}^{M}\Phi (l)\;|i[1]=i[2],Z^{i[1]};$$

that is $\hat{W}$ is the random variable *W* in the *i*th individual, conditional on it being produced by selfing and on the parental trait value. This is the quantity that we should like to prove is normally distributed. The first step is to find a suitable exchangeable pair. We write $\hat{\Phi }(l)$ for the conditioned version of Φ(l).

For each l∈{1,…,M}, let ${\hat{\Phi }}^{*}(l)$ be an independent draw from the conditional distribution of $\hat{\Phi }(l)$ given the sum of $\hat{\Phi }(m)$ over all m≠l; that is, in an obvious notation, ${\hat{\Phi }}^{*}(l)$ has the same distribution as

$$\Phi (l)|\;\sum_{m\neq l}\hat{\Phi }(m),i[1]=i[2],Z^{i[1]}.$$

Now let *L* be a uniform random variable on {1,…,M} and define

$${\hat{W}}^{\prime }=\hat{W}-\frac{\left(\hat{\Phi }(L)-{\hat{\Phi }}^{*}(L)\right)}{\sqrt{M}}.$$

Then $\left(\hat{W},{\hat{W}}^{\prime }\right)$ is an exchangeable pair.

Observe that

(G1)
$$\begin{array}{rl} E[\hat{W}-{\hat{W}}^{\prime }|\hat{W}] & =E\left[\frac{1}{\sqrt{M}}\frac{1}{M}\sum_{l=1}^{M}(\hat{\Phi }(l)-{\hat{\Phi }}^{*}(l))\;|\hat{W}\right]\\ & =\frac{1}{M}\hat{W}-\frac{1}{\sqrt{M}}\frac{1}{M}\sum_{l=1}^{M}E[{\hat{\Phi }}^{*}(l)|\hat{W}]\\ & \;:\;=\frac{1}{M}\hat{W}-T(\hat{W}). \end{array}$$

Remark G2 We wish to apply Corollary D4. Our first instinct is to write $E[{\hat{W}}^{\prime }\;|\;\hat{W}]=\hat{W}(1-1/M)+T(\hat{W})$ and take λ=1/M in Equation (D2). This will not suffice, as, with this choice, the first term on the right of Equation (D3) will be too big. As we shall see, the resolution is to take a larger value of λ which captures the dependence of ${\hat{W}}^{\prime }$ on $\hat{W}$.

Before we can apply Corollary D4, we need to investigate *T*. The first step is to establish the distribution of $\chi_{l}^{1},\chi_{l}^{2}$ conditional on i[1]=i[2], $Z^{i[1]}$ (which we shall for the rest of this section abbreviate to *Z*) and $W_{-l}$.

Keeping in mind Notation G1, and recalling that (Z,W) is shorthand for $\left(Z^{i[1]},A^{i}+D^{i}\right)$, we write P[z,w] for the density function of (Z,W) evaluated at (z,w) and $P_{z}[z,w]$, $P_{w}[z,w]$, and so on, for the corresponding partial derivatives. The proof of the following lemma mirrors those of Appendix E.

Lemma G3 The (unconditional) distribution of $\left(Z_{-l},W_{-l}\right)$ can be written as

$$\begin{array}{rl} & P[Z_{-l}=z,W_{-l}=w]\\ & \;=P[z,w]+\frac{1}{\sqrt{M}}E[\Psi_{l}]P_{z}[z,w]+\frac{1}{\sqrt{M}}E[\Phi_{l}]P_{w}[z,w]\\ & \;+\frac{1}{M}(E[\Psi_{l}{]}^{2}-E[\Psi_{l}^{2}])P_{zz}[z,w]\\ & \;+\frac{2}{M}(E[\Phi_{l}]E[\Psi_{l}]-E[\Phi_{l}\Psi_{l}])P_{zw}[z,w]\\ & \;+\frac{1}{M}(E[\Phi_{l}{]}^{2}-E[\Phi_{l}^{2}])P_{ww}[z,w]\\ & \;+\frac{1}{2M}E[\Psi_{l}^{2}]P_{zz}[z,w]+\frac{1}{M}E[\Phi_{l}\Psi_{l}]P_{zw}[z,w]\\ & \;+\frac{1}{2M}E[\Phi_{l}^{2}]P_{ww}[z,w]+O\left(\frac{1}{M^{3/2}}\right). \end{array}$$

Proof of Lemma G3 The key, as usual, is Taylor’s Theorem.

$$\begin{array}{rl} & P[Z_{-l}=z,W_{-l}=w]\\ & \;=\int \int P[\chi_{l}^{1}=x,\chi_{l}^{2}=x^{\prime },Z=z+\frac{1}{\sqrt{M}}\Psi_{l}(x,x^{\prime }),\\ & \;W=w+\frac{1}{\sqrt{M}}\Phi_{l}(x,x^{\prime })]dx\;dx^{\prime }\\ & \;=\int \int P[\chi_{l}^{1}=x,\chi_{l}^{2}=x^{\prime },Z=z,\\ & \;W=w]\;dx\;dx^{\prime }\\ & \;+\frac{1}{\sqrt{M}}\int \int \Psi_{l}(x,x^{\prime })\frac{\partial }{\partial z}P[\chi_{l}^{1}=x,\chi_{l}^{2}=x^{\prime },Z=z,\\ & \;W=w]\;dx\;dx^{\prime }\\ & \;+\frac{1}{\sqrt{M}}\int \int \Phi_{l}(x,x^{\prime })\frac{\partial }{\partial w}P[\chi_{l}^{1}=x,\chi_{l}^{2}=x^{\prime },Z=z,\\ & \;W=w]\;dx\;dx^{\prime }\\ & \;+\frac{1}{2M}\int \int \Psi_{l}^{2}(x,x^{\prime })\frac{\partial^{2}}{\partial z^{2}}P[\chi_{l}^{1}=x,\chi_{l}^{2}=x^{\prime },Z=z,\\ & \;W=w]\;dx\;dx^{\prime }\\ & \;+\frac{1}{M}\int \int \Psi_{l}(x,x^{\prime })\Phi_{l}(x,x^{\prime })\frac{\partial^{2}}{\partial z\partial w}P[\chi_{l}^{1}=x,\chi_{l}^{2}=x^{\prime },Z=z,\\ & \;W=w]\;dx\;dx^{\prime }\\ & \;+\frac{1}{2M}\int \int \Phi_{l}^{2}(x,x^{\prime })\frac{\partial^{2}}{\partial w^{2}}P[\chi_{l}^{1}=x,\chi_{l}^{2}=x^{\prime },Z=z,\\ & \;W=w]\;dx\;dx^{\prime }+O\left(\frac{1}{M^{3/2}}\right). \end{array}$$

Now write

$$\begin{array}{rl} & P[\chi_{l}^{1}=x,\chi_{l}^{2}=x^{\prime },Z=z,W=w]=P[\chi_{l}^{1}=x,\chi_{l}^{2}=x^{\prime }]\\ & \;\times P\left[Z_{-l}=z-\frac{1}{\sqrt{M}}\Psi_{l}(x,x^{\prime }),W_{-l}=w-\frac{1}{\sqrt{M}}\Phi_{l}(x,x^{\prime })\right]. \end{array}$$

Using the notation $P[x,x^{\prime },z,w]\;:\;=P[\chi_{l}^{1}=x,\chi_{l}^{2}=x^{\prime },Z=z,W=w]$, we substitute from above and apply Taylor’s Theorem to obtain,

P[x,x′,z,w]=P[χl1=x,χl2=x′]×∫∫P[y,y′,z−1MΨl(x,x′)+1MΨl(y,y′),w−1MΦl(x,x′)+1MΦl(y,y′)]dydy′=P[χl1=x,χl2=x′]{P[Z=z,W=w]1M∫∫+1M∫∫(Ψl(y,y′)−Ψl(x,x′))∂∂zP[y,y′,z,w]dydy′+1M∫∫(Φl(y,y′)−Φl(x,x′))∂∂wP[y,y′,z,w]dydy′+O(1M)}.

Differentiating with respect to *z* (and assuming sufficient regularity),

$$\begin{array}{rl} & \frac{\partial }{\partial z}P[\chi_{l}^{1}=x,\chi_{l}^{2}=x^{\prime },Z=z,W=w]=P[\chi_{l}^{1}=x,\chi_{l}^{2}=x^{\prime }]\\ & \;\times \{\frac{\partial }{\partial z}P[Z=z,W=w]+O\left(\frac{1}{M}\right)\\ & \;+\frac{1}{\sqrt{M}}\int \int (\Psi_{l}(y,y^{\prime })-\Psi_{l}(x,x^{\prime }))\frac{\partial^{2}}{\partial z^{2}}P[y,y^{\prime },z,w]\;dy\;dy^{\prime }\\ & \;+\frac{1}{\sqrt{M}}\int \int (\Phi_{l}(y,y^{\prime })-\Phi_{l}(x,x^{\prime }))\frac{\partial^{2}}{\partial z\partial w}P[y,y^{\prime },z,w]\;dy\;dy^{\prime }\}, \end{array}$$

and similarly

$$\begin{array}{rl} & \frac{\partial }{\partial w}P[\chi_{l}^{1}=x,\chi_{l}^{2}=x^{\prime },Z=z,W=w]=P[\chi_{l}^{1}=x,\chi_{l}^{2}=x^{\prime }]\\ & \;\times \{\frac{\partial }{\partial w}P[Z=z,W=w]+O\left(\frac{1}{M}\right)\\ & \;+\frac{1}{\sqrt{M}}\int \int (\Psi_{l}(y,y^{\prime })-\Psi_{l}(x,x^{\prime }))\frac{\partial^{2}}{\partial z\partial w}P[y,y^{\prime },z,w]\;dy\;dy^{\prime }\\ & \;+\frac{1}{\sqrt{M}}\int \int (\Phi_{l}(y,y^{\prime })-\Phi_{l}(x,x^{\prime }))\frac{\partial^{2}}{\partial w^{2}}P[y,y^{\prime },z,w]\;dy\;dy^{\prime }\}. \end{array}$$

As in the proof of Lemma E2, we only require the second derivatives to leading order

$$\begin{array}{rl} & \frac{\partial^{2}}{\partial z^{2}}P[\chi_{l}^{1}=x,\chi_{l}^{2}=x^{\prime },Z=z,W=w]\\ & \;=P[\chi_{l}^{1}=x,\chi_{l}^{2}=x^{\prime }]\frac{\partial^{2}}{\partial z^{2}}P[Z=z,W=w]+O\left(\frac{1}{\sqrt{M}}\right), \end{array}$$

with similar expressions for the other second partial derivatives. Substituting back into the first display yields the result. □

Lemma G4 The conditional distribution of $\chi_{l}^{1},\chi_{l}^{2}$ given *Z* and $W_{-l}$ is given by

(G2) $$\begin{array}{rl} & P[\chi_{l}^{1}=x,\chi_{l}^{2}=x^{\prime }|Z=z,W_{-l}=w_{-l}]\\ & \;=P[\chi_{l}^{1}=x,\chi_{l}^{2}=x^{\prime }]\left\{1-\frac{\Psi_{l}(x,x^{\prime })}{\sqrt{M}P[z,w_{-l}]}P_{z}[z,w_{-l}\right]\\ & \;+\frac{E[\Psi_{l}]}{\sqrt{M}P[z,w_{-l}]}P_{z}[z,w_{-l}]\}+O\left(\frac{1}{M}\right). \end{array}$$

Proof of Lemma G4 This is just an application of Bayes’ rule:

(G3) $$\begin{array}{rl} & P[\chi_{l}^{1}=x,\chi_{l}^{2}=x^{\prime }|Z=z,W_{-l}=w_{-l}]\\ & \;=\frac{P[Z=z,W_{-l}=w_{-l}|\chi_{l}^{1}=x,\chi_{l}^{2}=x^{\prime }]}{P[Z=z,W_{-l}=w_{-l}]}P[\chi_{l}^{1}=x,\chi_{l}^{2}=x^{\prime }]\\ & \;=\frac{P[Z_{-l}=z-\frac{\Psi_{l}(x,x^{\prime })}{\sqrt{M}},W_{-l}=w_{-l}]}{P[Z=z,W_{-l}=w_{-l}]}P[\chi_{l}^{1}=x,\chi_{l}^{2}=x^{\prime }]. \end{array}$$

Using Lemma G3 and Taylor’s Theorem,

$$\begin{array}{rl} & P\left[Z_{-l}=z-\frac{\Psi_{l}(x,x^{\prime })}{\sqrt{M}},W_{-l}=w_{-l}\right]\\ & \;=P\left[Z=z-\frac{\Psi_{l}(x,x^{\prime })}{\sqrt{M}},W_{-l}=w_{-l}\right]\\ & \;+\frac{1}{\sqrt{M}}E[\Psi_{l}]P_{z}\left[z-\frac{\Psi_{l}(x,x^{\prime })}{\sqrt{M}},w_{-l}\right]\\ & \;+\frac{1}{\sqrt{M}}E[\Phi_{l}]P_{w}\left[z-\frac{\Psi_{l}(x,x^{\prime })}{\sqrt{M}},w_{-l}\right]\\ & \;=P[Z=z,W=w_{-l}]-\frac{\Psi_{l}(x,x^{\prime })}{\sqrt{M}}P_{z}[z,w_{-l}]\\ & \;+\frac{E[\Psi_{l}]}{\sqrt{M}}P_{z}[z,w_{-l}]+\frac{E[\Phi_{l}]}{\sqrt{M}}P_{w}[z,w_{-l}]+O\left(\frac{1}{M}\right). \end{array}$$

When we integrate this expression with respect to *x* and $x^{\prime }$, to calculate the denominator in Equation (G3), we recover $P_{z}[z,w_{-l}]+\frac{E[\Phi_{l}]}{\sqrt{M}}P_{w}[z,w_{-l}]+O(\frac{1}{M})$ (since the expectation of $\Psi_{l}$ cancels). Expanding the ratio in Equation (G3) in powers of $1/\sqrt{M}$, the terms involving $E[\Phi_{l}]$ cancel, and the result follows.  □

Finally, we are in a position to calculate the quantity $T(\hat{W})$ that was defined in Equation (G1). Recall that ${\hat{\Phi }}_{l}^{*}$ is an independent draw from the conditional distribution of ${\hat{\Phi }}_{l}$ given $W_{-l}$ and *Z*, so using Equation (G2),

(G4)
$$\begin{array}{rl} E[{\hat{\Phi }}_{l}^{*}|\hat{W}] & =E[\Phi_{l}]-(E[\Phi_{l}\Psi_{l}]-E[\Phi_{l}]E[\Psi_{l}])\\ & \;\times \frac{1}{\sqrt{M}}E\left[\frac{1}{P[Z,W_{-l}]}\frac{\partial }{\partial z}P[Z,W_{-l}]\;|W=\hat{W}\right]\\ & \;+O\left(\frac{1}{M}\right). \end{array}$$

Conditioning only on i[1]=i[2], using the calculations in Appendix B and Equation (F6), by an application of Theorem D2 (up to an error of order $1/\sqrt{M}$) the joint distribution of $\left(A^{i}+D^{i},Z^{i[1]}\right)$ is approximately that of a bivariate normal.

We will need that for a bivariate normal distribution with mean vector $\left(\mu_{Z},\mu_{W}\right)$ and covariance matrix

$$\left(\begin{array}{cc} \sigma_{Z}^{2} & Cov(Z,W)\\ Cov(Z,W) & \sigma_{W}^{2} \end{array}\right)$$

the density function takes the form

$$\begin{array}{rl} & p(z,w)=\frac{1}{2\pi \sigma_{Z}\sigma_{W}\sqrt{1-\rho^{2}}}\\ & \;\times \exp\;\{-\frac{1}{2(1-\rho^{2})}(\frac{(z-\mu_{Z}{)}^{2}}{\sigma_{Z}^{2}}-\frac{2\rho (z-\mu_{Z})(w-\mu_{W})}{\sigma_{Z}\sigma_{W}}\\ & \;+\frac{(w-\mu_{W}{)}^{2}}{\sigma_{W}^{2}})\}, \end{array}$$

where $\rho =Cov(Z,W)/(\sigma_{Z}\sigma_{W})$. Differentiating, we find

(G5)
$$\frac{1}{p(z,w)}\frac{\partial }{\partial z}p(z,w)=\frac{1}{\left(1-\rho^{2}\right)}\left\{\frac{\rho (w-\mu_{W})}{\sigma_{Z}\sigma_{W}}-\frac{\left(z-\mu_{Z}\right)}{\sigma_{Z}^{2}}\right\}.$$

Recall the definition of *T* from Equation (G1). Multiplying Equation (G4) by $1/\sqrt{M}$, observing that $E[W_{-l}|W,Z]=W+O(1/\sqrt{M})$ (and since $\Phi_{l}$ is uniformly bounded independent of *l*, the error is bounded independent of *l*), and then averaging out over *l* as in the definition of $T(\hat{W})$, on substituting Equation (G5) and $Cov(Z,W)=\rho \sigma_{Z}\sigma_{W}$, we find

(G6)
$$\begin{array}{rl} & T(\hat{W})\\ & \;=\frac{1}{M}E[W]+\frac{Cov(Z,W)}{M}\left\{\frac{Z-E[Z]}{\sigma_{Z}^{2}(1-\rho^{2})}-\frac{\rho }{1-\rho^{2}}\frac{\hat{W}-E[W]}{\sigma_{Z}\sigma_{W}}\right\}\\ & \;+O(\frac{1}{M^{3/2}})\\ & \;=\frac{1}{M}E[W]+\frac{\rho \sigma_{Z}\sigma_{W}}{M}\left\{\frac{Z-E[Z]}{\sigma_{Z}^{2}(1-\rho^{2})}-\frac{\rho }{1-\rho^{2}}\frac{\hat{W}-E[W]}{\sigma_{Z}\sigma_{W}}\right\}\\ & \;+O\left(\frac{1}{M^{3/2}}\right)\\ & \;=\frac{1}{M}E[W]+\frac{1}{M}\rho \frac{\sigma_{W}}{\sigma_{Z}}\frac{Z-E[Z]}{1-\rho^{2}}-\frac{1}{M}\frac{\rho^{2}}{1-\rho^{2}}(\hat{W}-E[W])\\ & \;+O\left(\frac{1}{M^{3/2}}\right). \end{array}$$

Using the approximation for the conditional distribution of $\left(\chi_{l}^{1},\chi_{l}^{2}\right)$, given *Z* obtained in Appendix E,

$$E[\hat{W}]=E[W|Z]=E[W]+\rho \frac{\sigma_{w}}{\sigma_{z}}(Z-E[Z])+O\left(\frac{1}{\sqrt{M}}\right),$$

so we can rewrite Equation (G6) as

$$T(\hat{W})=\frac{1}{M}\frac{1}{\left(1-\rho^{2}\right)}E[\hat{W}]-\frac{1}{M}\frac{\rho^{2}}{1-\rho^{2}}\hat{W}+O\left(\frac{1}{M^{3/2}}\right).$$

Substituting in Equation (G1),

(G7)
$$E[\hat{W}-{\hat{W}}^{\prime }|\hat{W}]=\frac{1}{M}\frac{1}{1-\rho^{2}}(\hat{W}-E[\hat{W}])+O\left(\frac{1}{M^{3/2}}\right).$$

We are going to apply Corollary D4 to $\left(\hat{W},{\hat{W}}^{\prime }\right)$ with $F=\sigma (\hat{W})$. We set $\lambda =1/(M(1-\rho^{2}))$ and observe from Equation (G7) that we may take a remainder term *R* with E[|R|] of order $1/M^{1/2}$ in Equation (D3). Moreover,

$${\hat{K}}_{2}=\frac{1}{2\lambda }\frac{|\Delta {|}^{3}}{2},$$

and so, since by construction $|\Delta |<C/\sqrt{M}$, $E[{\hat{K}}_{2}]$ is also order at most $1/M^{1/2}$.

Since with these definitions

$${\hat{K}}_{1}=\frac{M(1-\rho^{2})}{2}E[(\hat{W}-{\hat{W}}^{\prime }{)}^{2}|\hat{W}],$$

it remains to control

(G8)
$$E\left[\left|\sigma_{\hat{W}}^{2}-\frac{M(1-\rho^{2})}{2}E[(\hat{W}-{\hat{W}}^{\prime }{)}^{2}|\hat{W}]\right|\right].$$

Again using the results of Appendix E,

$$\sigma_{\hat{W}}^{2}=(1-\rho^{2})\sigma_{W}^{2}+O\left(\frac{1}{\sqrt{M}}\right),$$

(the first term being the conditional variance if the random variables were distributed exactly as a bivariate normal), whereas

$$\begin{array}{rl} E[E[(\hat{W}-{\hat{W}}^{\prime }{)}^{2}|\hat{W}]] & =E[(\hat{W}-{\hat{W}}^{\prime }{)}^{2}]\\ & =E\left[\frac{1}{M^{2}}\sum_{l=1}^{M}({\hat{\Phi }}_{l}-{\hat{\Phi }}_{l}^{*}{)}^{2}\right]\\ & =2\frac{1}{M}\sigma_{W}^{2}+O\left(\frac{1}{M^{3/2}}\right). \end{array}$$

(Note that we see the unconditioned $\sigma_{W}^{2}$ in this second expression since it involves only diagonal terms.)

To control Equation (G8), observing that, by the Cauchy–Schwarz inequality,

$$\begin{array}{rl} & E[|E[M(\hat{W}-{\hat{W}}^{\prime }{)}^{2}]-E[M(\hat{W}-{\hat{W}}^{\prime }{)}^{2}|\hat{W}]|]\\ & \;\leq Var(E[M(\hat{W}-\hat{W}{]}^{\prime }{)}^{2}|\hat{W}]{)}^{1/2}, \end{array}$$

it suffices to control

$$Var(E[M(\hat{W}-{\hat{W}}^{\prime }{)}^{2}|\hat{W}]).$$

In particular, we should like to show that this expression is of order O(1/M).

Now we use the standard decomposition of conditional expectations: for two random variables *X* and *F*,

$$\begin{array}{rl} Var(X) & =E[E[X^{2}\;|\;F]-(E[X\;|\;F]{)}^{2}+(E[X\;|\;F]{)}^{2}]-E[E[X\;|\;F]{]}^{2}\\ & =E[Var(X\;|\;F)]+Var(E[X\;|\;F]). \end{array}$$

So

Var(E[X|F])=Var(X)−E[Var(X|F)].

For us, $X=M(\hat{W}-{\hat{W}}^{\prime }{)}^{2}=(\Phi_{L}-\Phi_{L}^{*}{)}^{2}$, and $F=\hat{W}$, so

$$Var(X)=\frac{1}{M}\sum_{l=1}^{M}E[(\Phi_{l}-\Phi_{l}^{*}{)}^{4}]-{\left(\frac{1}{M}\sum_{l=1}^{M}E[(\Phi_{l}-\Phi_{l}^{*}{)}^{2}]\right)}^{2},$$

and we seek

$$\begin{array}{rl} & \frac{1}{M}\sum_{l=1}^{M}E[(\Phi_{l}-\Phi_{l}^{*}{)}^{4}]-{\left(\frac{1}{M}\sum_{l=1}^{M}E[(\Phi_{l}-\Phi_{l}^{*}{)}^{2}]\right)}^{2}\\ & \;-\frac{1}{M}\sum_{l=1}^{M}E[E[(\Phi_{l}-\Phi_{l}^{*}{)}^{4}]|\hat{W}]+E\left[{\left(\frac{1}{M}\sum_{l=1}^{M}E[(\Phi_{l}-\Phi_{l}^{*}{)}^{2}|\hat{W}]\right)}^{2}\right], \end{array}$$

where the expectation is with respect to the distribution of $\hat{W}$. By the tower property, the terms involving $(\Phi_{l}-\Phi_{l}^{*}{)}^{4}$ cancel, leaving

(G9)
$$\begin{array}{rl} & \frac{1}{M^{2}}\sum_{l=1}^{M}\sum_{m=1}^{M}\left\{E[E[(\Phi_{l}-\Phi_{l}^{*}{)}^{2}|\hat{W}]E[(\Phi_{m}-\Phi_{m}^{*}{)}^{2}|\hat{W}]\right]\\ & \;-E[(\Phi_{l}-\Phi_{l}^{*}{)}^{2}]E[(\Phi_{m}-\Phi_{m}^{*}{)}^{2}]\}. \end{array}$$

Expanding $E[(\Phi_{l}-\Phi_{l}^{*}{)}^{2}|\hat{W}]E[(\Phi_{m}-\Phi_{m}^{*}{)}^{2}|\hat{W}]$ in an entirely analogous way to Equation (G4), when we take expectations, using the tower property of conditional expectations, the part of the product that is an affine function of $\hat{W}$ will cancel in Equation (G9), leaving quadratic (and higher order) terms, each of which is of order O(1/M) in the summand. Overall then Equation (G9) is O(1/M), and applying Corollary D4, the proof that $A^{i}+D^{i}$ is normal with an error of order $1/\sqrt{M}$ is complete.

### The residuals, generation one

Proving that $R_{A}^{i}+R_{D}^{i}$ is normal is much simpler. Since Mendelian inheritance is independent across loci, we are able to use Theorem D2 in much the same way as in generation zero. A combination of Lemma E2 and Bayes’ rule suffices to show that the variance is not affected by conditioning on parental trait values, after which the proof proceeds essentially as in the additive case and so is omitted.

## Appendix H: Generation *t*, accumulation of information

If we wanted to prove a strict analog of the results of Barton *et al.* (2017) in the additive case, then we would want to condition not just on the trait values of the parents, but on the trait values of an arbitrary collection of individuals in the pedigree. Such a proof can follow essentially the same lines as above, although the calculations are considerably longer to write out. The only thing that must be checked is that we do not accumulate too much information from knowing those trait values; it is this that controls for how long the infinitesimal approximation will remain accurate. This requires more care than the additive case of Barton *et al.* (2017), so we present the argument here.

Recall that we write P(t) for the pedigree up to and including generation *t* and Z(t) for the corresponding vector of trait values of all individuals in P(t). We would like to understand the distribution of the allelic types $\chi_{l}^{1}(j^{*}),\chi_{l}^{2}(j^{*})$ at locus *l* of an individual $j^{*}$ in generation *t*, conditional on knowing the trait values of all individuals in the pedigree up to generation t−1. That is, we would like to estimate

(H1)
$$\begin{array}{rl} & P[(\chi_{l}^{1}(j^{*}),\chi_{l}^{2}(j^{*}))=(x,x^{\prime })\;|\;P(t),Z(t-1)=(z^{j}{)}_{\;j\in P(t-1)}]\\ & \;=\frac{P[Z(t-1)=(z^{j}{)}_{\;j\in P(t-1)}\;|\;(\chi_{l}^{1}(j^{*}),\chi_{l}^{2}(j^{*}))=(x,x^{\prime }),P(t)]}{P[Z(t-1)=(z^{j}{)}_{\;j\in P(t-1)}\;|\;P(t)]}\\ & \;\times P[(\chi_{l}^{1}(j^{*}),\chi_{l}^{2}(j^{*}))=(x,x^{\prime })\;|\;P(t)]. \end{array}$$

To estimate the numerator in the fraction, we partition over the possible patterns of identity at locus *l* in the pedigree, conditional on that pedigree; that is we condition on the values of the Bernoulli random variables that determine Mendelian inheritance at locus *l* across the pedigree. We denote this $M_{l}(t)$ and abuse notation by writing $\left(M_{l}^{1}(j),M_{l}^{2}(j)\right)$ for the allelic states at locus *l* in individual j∈P(t−1) conditional on $M_{l}(t)$. More precisely, if $\chi_{l}^{1}(j^{*})=x$ and $\chi_{l}^{2}(j^{*})=x^{\prime }$, $\left(M_{l}^{1}(j),M_{l}^{2}(j))=(y,y^{\prime }),(y,x^{\prime }),(x,y^{\prime }),(x,x^{\prime }\right)$ according to whether *j* is identical by descent with the chosen individual $j^{*}$ on neither chromosome, one chromosome or both chromosomes. We use $E_{M_{l}}$ when we wish to emphasize that we are taking the expectation with respect to this quantity. We proceed as in Lemma E6:

$$\begin{array}{rl} & P[Z(t-1)=(z^{j}{)}_{\;j\in P(t-1)}\;|\;(\chi_{l}^{1}(j^{*}),\chi_{l}^{2}(j^{*}))=(x,x^{\prime }),\\ & \;P(t),M_{l}(t)]\\ & \;=P\left[Z_{-l}^{j}=z^{j}-\frac{1}{\sqrt{M}}\Psi_{l}(M_{l}^{1}(j),M_{l}^{2}(j)),\;\forall j\in P(t-1)\;|\;P(t)\right]\\ & \;=E[P\left[Z^{j}=z^{j}-\frac{1}{\sqrt{M}}\Psi_{l}(M_{l}^{1}(j),M_{l}^{2}(j)\right)\\ & \;+\frac{1}{\sqrt{M}}\Psi_{l}(\chi_{l}^{1}(j),\chi_{l}^{2}(j)),\;\forall j\in P(t-1)\;|\;P(t)]], \end{array}$$

where in the last line the expectation is taken with respect to the unconditional law of the random family $\left\{(\chi_{l}^{1}(j),\chi_{l}^{2}(j)),\;j\in P(t-1)\right\}$.

Substituting in Equation (H1), in an obvious notation,

P[(χl1(j*),χl2(j*))=(x,x′)|P(t),Z(t−1)=z]=P[(χl1(j*),χl2(j*))=(x,x′)|P(t)]×(1−∑j∈P(t−1)1M{E[Ψl(Ml1(j),Ml2(j))|P(t)]−E[Ψl(χl1(j),χl2(j))|P(t)]}PZj[z]P[z]∑j∈P(t−1))+O(1M).

In particular, the summand will vanish if *j* and $j^{*}$ are not identical by descent in at least one copy at locus *l*, since then the allelic states at locus *l* in individuals *j* and $j^{*}$ are independent. Furthermore, the more distant the relationship between *j* and $j^{*}$ (that is, the smaller the probability of their being identical by descent), the less information we glean about the allelic states in $j^{*}$ from observing the trait value of individual *j*, resulting in a small contribution of the *j*th term to the difference between the conditional and unconditional laws of $\left(\chi_{l}^{1}(j^{*}),\chi_{l}^{2}(j^{*})\right)$. The infinitesimal model can be expected to break down for an individual if we know that one of its close relatives had a particularly extreme trait value, or if the pedigree is particularly inbred (so that there is little variation between offspring).

## Appendix I: Supplementary material and codes

The following supplementary material can be found in the public repository (Barton 2023):

- The *Mathematica* notebook Algorithm for calculating identities.nb, comprising a set of codes to compute the identity coefficients of Appendix A.
- The *Mathematica* notebook Infinitesimal with dominance.nb, accompanying and complementing the simulations and figures presented in the paper.
- The different datasets from which the numerical examples in this paper can be reproduced.
